# Accuracy and precision of continuous, non‐invasive blood pressure devices: a systematic review and meta‐analyses

**DOI:** 10.1111/anae.70250

**Published:** 2026-06-15

**Authors:** Stefanie Lischker, Gunther J. Pestel, Irene Schmidtmann, Eva‐Verena Griemert

**Affiliations:** ^1^ Department of Anaesthesiology University Medical Center of the Johannes Gutenberg‐University Mainz Germany; ^2^ Institute of Medical Biostatistics, Epidemiology and Informatics (IMBEI) University Medical Center of the Johannes Gutenberg‐University Mainz Germany

**Keywords:** blood pressure, continuous blood pressure measurement, meta‐analysis, non‐invasive

## Abstract

**Introduction:**

Reliable continuous non‐invasive blood pressure monitoring technology should support clinicians to maintain blood pressure within appropriate limits peri‐operatively. Previous studies have shown conflicting results regarding measurement accuracy.

**Methods:**

This systematic review and six meta‐analyses evaluated the accuracy and precision of non‐invasive continuous arterial blood pressure measurement technologies (volume clamp technology, applanation tonometry and photoplethysmography) compared with invasive arterial measurement in adults. We analysed pooled bias (mean difference between non‐invasive and invasive measurement), standard deviation and limits of agreement. In each meta‐analysis, mean arterial pressure (MAP), systolic blood pressure (SBP) and diastolic blood pressure (DBP) were analysed separately. Acceptability was defined according to Association for the Advancement of Medical Instrumentation® (AAMI) criteria.

**Results:**

Sixty‐five studies were included: two meta‐analyses for volume clamp technology (ClearSight™, CNAP™); three meta‐analyses for applanation tonometry (T‐Line™, Vasotrac™ and N‐CAT™); and one meta‐analysis for photoplethysmography technology. For volume clamp technologies, none met AAMI criteria for bias and SD in relation to MAP, SBP and DBP. T‐Line, Vasotrac and N‐CAT met the AAMI criteria for bias for MAP (2.53 mmHg, 0.36 mmHg, ‐0.37 mmHg), SBP (‐0.07 mmHg, 1.20 mmHg, ‐3.68 mmHg) and DBP (3.09 mmHg, 1.09 mmHg, 0.84 mmHg), respectively. In terms of standard deviation, only N‐CAT (MAP 6.97 mmHg, SBP 7.14, mmHg, DBP 6.55 mmHg) met AAMI criteria for all blood pressure parameters. Photoplethysmography technology met AAMI criteria for bias (MAP ‐0.09 mmHg, SBP 0.49 mmHg, DBP ‐0.26 mmHg) and standard deviation (MAP 6.86 mmHg, DBP 6.38 mmHg).

**Discussion:**

Overall, the AAMI criteria for bias and SD were not fulfilled for most of the devices tested and wide limits of agreement are of concern. One applanation tonometry device (N‐CAT) shows promise. Clinicians should be aware of the lack of interchangeability with invasive blood pressure management.

## Plain Language Summary

**What we did:**

Researchers looked at 65 studies that tested different non‐invasive machines used to measure blood pressure continuously. They compared these machines with the standard invasive method (putting a tube into an artery), which is usually more accurate. The technologies studied included volume clamp devices, applanation tonometry devices and photoplethysmography devices. The researchers checked how close the non‐invasive readings were to the invasive readings.

**Why did we do it:**

Doctors need accurate blood pressure readings during and after surgery so they can keep patients safe. New non‐invasive machines are easier and safer to use, but it is important to know whether they are accurate enough to replace invasive monitoring. The researchers wanted to find out which devices worked best.

**What we found:**

Most of the non‐invasive devices were not accurate enough to fully match the standard invasive method. Some devices gave readings that were too different from the invasive readings, which could make treatment decisions harder. One device called N‐CAT performed better than most others and showed promising results. Overall, the study found that doctors should be careful when using these non‐invasive devices because they may not always give the same results as invasive blood pressure monitoring.

## Introduction

Rapid intra‐operative changes in blood pressure may have profound implications for patient outcomes. Intra‐operative hypertension increases the risk of myocardial injury [1] and haemorrhage. Conversely, intra‐operative hypotension is also associated with myocardial injury [1, 2] as well as myocardial infarction [2, 3, 4], acute kidney injury [5, 6, 7] and higher risk of cerebrovascular events up to 30 days post‐surgery [8]. Severity of injury and mortality increase with duration of hypotension [5, 9]. Thus, early detection or even prediction of a potentially relevant change in blood pressure could significantly improve patient outcomes.

Traditionally, blood pressure is measured intermittently (every 3–5 min) in routine clinical care [10], typically using cuff‐based devices or oscillometric monitors. However, oscillometric blood pressure measurements tend both to overestimate blood pressure in hypotension and to underestimate blood pressure in hypertension compared with invasive intra‐arterial measurement [11]. Blood pressure changes due to anaesthesia, vasodilatation or application of vasoactive drugs may remain undetected, thus delaying treatment to avoid organ damage [10, 12]. Nearly twice as many hypotension episodes are detected with continuous invasive blood pressure monitoring compared with oscillometric measurements [13]. Therefore, continuous blood pressure monitoring intra‐operatively supports proactive and personalised care. Invasive blood pressure measurement by arterial cannulation is considered the clinical gold standard of care [14, 15] but carries inherent risks [16, 17, 18, 19]. To reduce these, several devices using various technological approaches are available. Volume clamp technology (also known as the vascular unloading technique) [20] is based on the Penáz principle [21] using a photoplethysmography sensor to detect changes in finger artery pulsation [22]. Applanation tonometry applies a defined force to the wall of a superficial artery. A force sensor measures the arterial deformation induced by blood flow over a defined number of heartbeats [23]. Photoplethysmography technology is an optical measurement technology based on pulse oximetry that can be used to determine pulse wave velocity, from which blood pressure parameters are estimated [24].

The concordance between non‐invasive continuous blood pressure measurement techniques and invasive arterial pressure measurement has been evaluated in previous studies [14, 15]. However, focusing on a differentiation of the measurement results according to the respective measurement technologies has not been done.

## Methods

A systematic review and six meta‐analyses were performed comparing continuous non‐invasive blood pressure measurement technologies with invasive radial arterial pressure measurements in adult patients undergoing surgery or receiving care in the ICU.

Considering each measurement technique separately, the primary goal was to examine accuracy and precision of continuous non‐invasive mean arterial pressure (MAP), systolic blood pressure (SBP) and diastolic blood pressure (DBP). While the ability to follow trends is relevant, this study focused on validity and reliability of measurements as expressed by accuracy and precision. These were defined following Association for the Advancement of Medical Instrumentation (AAMI) criteria, which have been used most commonly in previous studies [14, 25, 26, 27, 28]. Mean bias (e.g. mean difference, accuracy) and its standard deviation (e.g. SD, precision) for MAP, SBP and DBP were considered acceptable if they were < 5 mmHg (bias) and < 8 mmHg (SD) [29, 30]. Hence, an acceptable difference between upper and lower limits of agreement was 32 mmHg. All analyses adhered to PRISMA guidelines (Fig. 1) [31].

**Figure 1:**
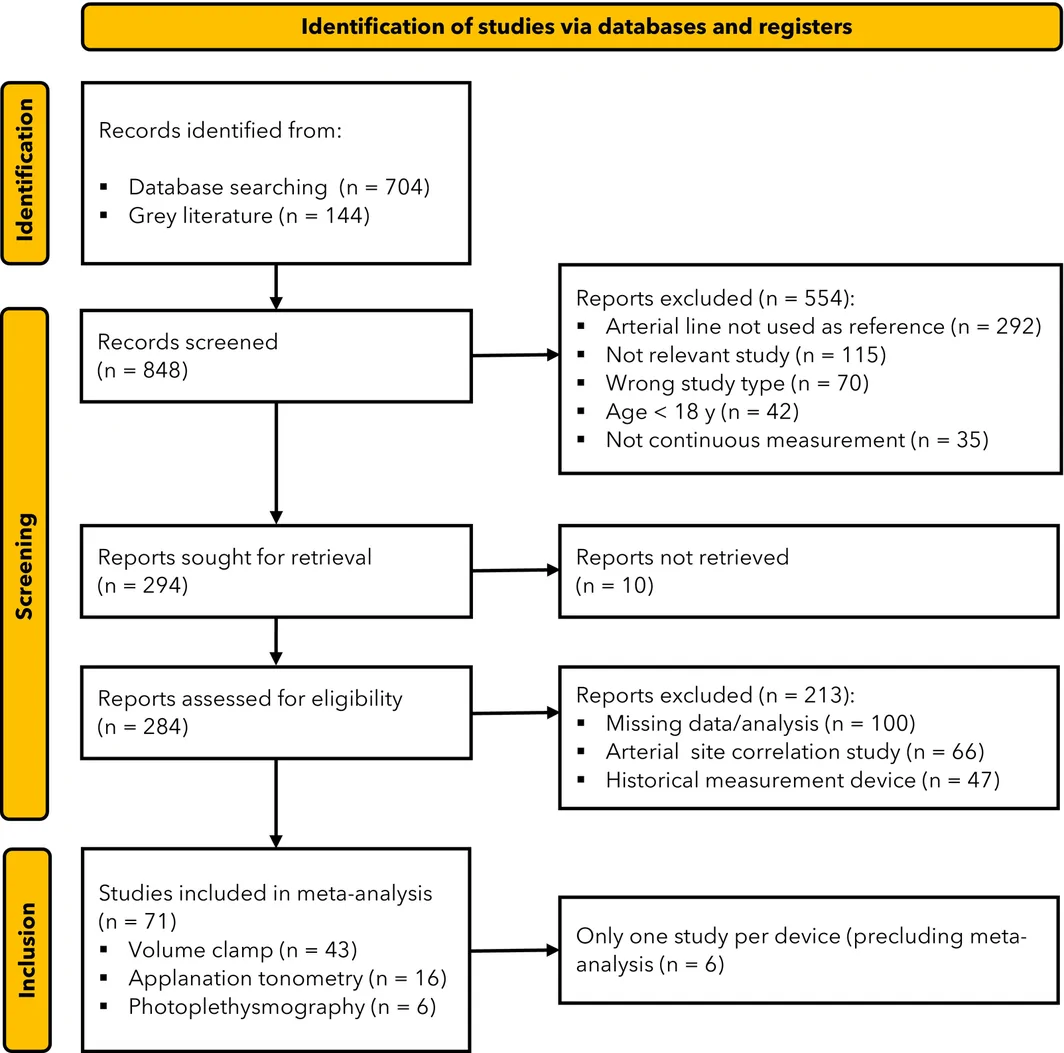
Study flow diagram.

Electronic searches were performed in: (PubMed; Web of Science; Cochrane Central Register of Controlled Trials); and LIVIVO. Databases were screened between April 2023 and September 2024. The details of the search strategy using single‐word search and the PICO tool (patient, intervention, comparison, outcome) [32] are available in online Supporting Information Appendix S1. In addition, the reference lists of several studies were reviewed and additional studies included if the criteria described below were met. Manufacturer websites were also searched for relevant studies.

Articles were included if they met the following criteria: full text articles of studies comparing continuous non‐invasive blood pressure measurement with direct blood pressure measurement in the radial artery; patients in the operating theatre or ICU; patients aged ≥ 18 y; English or German language; and cohort studies (prospective and retrospective) using a statistical analysis according to Bland and Altman [33] extracting either bias and SD of the difference between method, or bias and 95% limits of agreement.

Femoral and central intra‐arterial blood pressure are significantly different from radial intra‐arterial pressure [34]. Since most studies are based on the comparison with radial blood pressure measurement due to its greater availability in clinical practice, we chose this reference method. Chosen outcome parameters were: bias; SD; and the upper and lower limits of agreement (defined as bias ± 2 SD) for MAP, SBP and DBP between the different non‐invasive blood pressure devices and the invasive arterial blood pressure measurement technology.

We performed a separate evaluation for each measurement device that had multiple studies comparing the respective measurement technique with invasive radial blood pressure measurement. Volume clamp technology: one finger cuff ClearSight™ (Edwards Lifescience, Irvine, CA, USA) formerly known as Nexfin™ (Nexfin HD, BMEYE, Amsterdam, the Netherlands); and two finger cuffs (CNAP™, CNSystem Medizintechnik AG, Graz, Austria). Three systems of applanation tonometry: T‐Line™ (Tensys Medical Inc, San Diego, CA, USA); Vasotrac™ (Medwave, Arden Hills, MN, USA); and N‐CAT™ (Nellcor, Hayward, CA, USA). Photoplethysmography technology: OptiBP™ (Biospectal Inc., Lausanne, Switzerland [35]); Aktiia OBPM technology (Aktiia SA, Neuchâtel, Switzerland [36]); and NICAP technology (Zhejiang Union Medical Equipment Co., Ltd., Hangzhou, China). This evaluation approach led to six different meta‐analyses. As Nexfin uses an updated implementation of the Finapres™ (Finapres Medical Systems B.V, Enschede, Netherlands) method [37, 38], Finapres was not included in the meta‐analyses.

For every meta‐analysis, the pooled results of MAP, SBP and DBP were analysed separately. References were managed using Citavi 6.0 Reference Management and Knowledge Organization (Swiss Academic Software, Wädenswil, Switzerland).

The characteristics of the individual studies and extracted outcomes from studies were transferred to a standard Microsoft Excel (Microsoft Corp., Redmond, WA, USA) spreadsheet (online Supporting Information Tables S1–S5).

At least two authors (EG, SL and/or IS) independently assessed the risk of bias throughout the process using the revised Quality Assessment of Diagnostic Accuracy Studies (QUADAS‐2) tool [39, 40]. We analysed each study included in a meta‐analysis according to the categories ‘patient selection’, ‘index test’, ‘reference standard’ and ‘flow and timing’ to classify it as low risk, unclear risk or high risk. Based on QUADAS‐C, two further signalling questions were added to address the proper application of the Bland–Altman method: ‘multiple measurements per patient taken into account correctly’; and ‘check for heterogeneity of variance and dependence of bias on average value of measurement performed’ (online Supporting Information Table S6). The overall risk of bias was considered low only if all categories showed a low risk of bias. Conflicting results or discrepancies were resolved by a third author and by discussing the conflicts.

Each meta‐analysis consisted of data analyses from one of the six different measurement techniques, resulting in six meta‐analyses. The data analyses were based on the calculation of pooled mean difference (bias), SD and limits of agreement between non‐invasive measurements (index test) and invasive arterial measurements (reference test), which were evaluated separately for MAP, SBP and DBP, respectively. For comparability, we standardised bias by referring to non‐invasive monitoring device minus invasive device, using invasive monitoring as the baseline comparator group.

In order to illustrate the heterogeneity and inconsistency between included studies [41], we applied τ^2^ [42]. For every meta‐analysis of the respective measurement technology, we calculated pooled estimates of bias and limits of agreement using the method suggested by Tipton and Shuster [43] for multiple measurements per patient with a robust variance estimate. This produced valid inference for limits of agreement even if the model was mis‐specified in the original analysis. The analysis was performed using R 4.5.1 [44]. The functions provided by Tipton and Shuster [43] were used to implement the method for the analysis presented here. Sensitivity analyses included funnel plots, Baujat plots, influence diagnostics using the influence function of the metafor package and cluster‐robust estimation of confidence intervals using the robust function of the metafor package. Where influential studies were detected according to the default criteria of the influence function, we flagged these studies in online Supporting Information Figures S1–S5 with ‘*’. We obtained graphs and performed sensitivity analyses using the metafor package 4.8‐0 [44].

## Results

Figure 1 shows the search process according to PRISMA guidelines [31, 45]. In total, 704 studies from database searching and 144 studies were identified through additional searches, of which 71 met all eligibility criteria. Six were not eligible for meta‐analysis as only one study per device existed. The remaining 65 were included in the meta‐analyses, separated by their respective technology. We made no distinctions between study settings (e.g. operating theatre vs. ICU) as only a few studies in the ICU‐setting were available for meta‐analyses. All studies were published between 1993 and 2022.

Forty‐three studies addressed volume clamp technology: ClearSight [20, 25, 28, 38, 46, 47, 48, 49, 50, 51, 52, 53, 54, 55, 56, 57, 58, 59, 60, 61, 62, 63, 64, 65, 66, 67]; and CNAP [16, 26, 27, 68, 69, 70, 71, 72, 73, 74, 75, 76, 77, 78, 79, 80]. Sixteen studies addressed applanation tonometry: T‐Line [81, 82, 83, 84, 85, 86, 87, 88, 89]; Vasotrac [90, 91, 92, 93]; and N‐CAT [94, 95, 96]. Six studies used photoplethysmography technology [24, 35, 36, 97, 98, 99]. Six studies [100, 101, 102, 103, 104, 105] could not be included in the meta‐analyses, as to our knowledge, only one study per device exists. Information regarding these studies is shown in online Supporting Information Tables S1–S5.

A summary of the pooled values of the individual meta‐analyses as well as results of heterogeneity‐analysis (τ^2^) are shown in Table 1 with a concise summary in Table 2. A more detailed description of the included studies is available in online Supporting Information Tables S1–S5 and S7.

**Table 1:** Summary of the pooled values of the individual meta‐analyses. Bias, standard deviation (SD), variance between studies (τ)^2^ and limits of agreement are shown in mmHg.

Measurement technology	Blood pressure parameter	Included studies	Measurements	Patients	Bias (SD)	*τ* ^2^	Limits of agreement
Volume clamp technology							
ClearSight	MAP	32	229,182	1072	**3.40** (8.96)	12.72	‐15.89–22.69
	SBP	28	230,894	907	**‐1.14** (13.23)	47.65	‐30.99–28.71
	DBP	27	230,685	884	5.76 (8.29)	19.10	‐12.98–24.51
CNAP	MAP	17	1,104,234	849	**3.89** (9.65)	9.53	‐16.37–24.15
	SBP	16	1,103,107	839	**‐1.95** (14.20)	12.18	‐31.19–27.29
	DBP	16	1,104,085	839	6.34 (9.48)	12.32	‐13.87–26.55
Applanation technology							
T‐Line	MAP	9	273,204	215	**2.53 (6.60)**	1.57	‐10.92–15.97
	SBP	8	245,304	201	**‐0.07** (9.15)	3.86	‐18.65–18.78
	DBP	8	245,304	201	**3.09 (7.17)**	0.00	‐11.25–17.44
Vasotrac	MAP	4	64,654	131	**0.36 (5.97)**	0.89	‐11.73–12.44
	SBP	3	64,347	116	**1.20** (10.22)	10.95	‐20.28–22.68
	DBP	3	64,347	116	**1.09 (7.01)**	3.00	‐13.35–15.53
N‐CAT	MAP	4	89,558	48	**‐0.37 (6.97)**	2.99	‐14.73–13.98
	SBP	3	1400	32	**‐3.68 (7.14)**	2.04	‐18.23–10.88
	DBP	3	1400	32	**0.84 (6.55)**	8.17	‐13.45–15.13
Photoplethysmography							
	MAP	5	15,631	117	**‐0.09 (6.86)**	0.00	‐13.80–13.68
	SBP	7	16,273	159	**0.49** (9.63)	0.00	‐18.78–19.76
	DBP	8	16,435	179	**‐0.26 (6.38)**	0.00	‐13.02–12.50

MAP, mean arterial pressure; SBP, systolic blood‐pressure; DBP, diastolic blood‐pressure. Analyses where Advancement of Medical Instrumentation criteria were met are highlighted in bold.

**Table 2:** Summary of the pooled values of the individual meta‐analyses. Bias and limits of agreement are measured in mmHg.

	Mean arterial pressure			Systolic blood pressure			Diastolic blood pressure		
	n	Bias (SD)	Limits of agreement	n	Bias (SD)	Limits of agreement	n	Bias (SD)	Limits of agreement
Volume clamp technology									
ClearSight	907	**3.40** (8.96)	‐15.89–22.69	907	**‐1.14** (13.23)	‐30.99–28.71	884	5.76 (8.29)	‐12.98–24.52
CNAP	849	**3.89** (9.65)	‐16.37–24.15	839	**‐1.95** (14.20)	‐31.19–27.29	839	6.34 (9.48)	‐13.87–26.55
Applanation tonometry									
T‐Line	215	**2.53** (**6.60**)	‐10.92–15.97	201	**‐0.07** (9.15)	‐18.65–18.78	201	**3.09 (7.17)**	‐11.25–17.44
Vasotrac	131	**0.36** (**5.97**)	‐11.73–12.44	116	**1.20** (10.22)	‐20.28–22.68	116	**1.09 (7.01)**	‐13.35–15.53
N‐CAT	48	**‐0.37** (**6.97**)	‐14.73–13.98	32	**‐3.68** (**7.14**)	‐18.23–10.88	32	**0.84 (6.55)**	‐13.45–15.13
Photoplethysmography									
	117	**‐0.09** (**6.86**)	‐13.80–13.63	159	**0.49** (9.63)	‐18.78–19.76	179	**‐0.26 (6.38)**	‐13.02–12.50

SD, standard deviation. Analyses where Advancement of Medical Instrumentation criteria were met are highlighted in bold.

Thirty‐two studies using volume clamp technique with one finger cuff (ClearSight) were evaluated for MAP (907 patients, 230,894 measurements) and SBP (907 patients, 230,894 measurements), respectively; 27 were evaluated for DBP (884 patients, 230,685 measurements). A pooled bias (SD) of 3.4 (8.96) mmHg and limits of agreement of ‐15.89–22.69 mmHg were shown for MAP; ‐1.14 (13.23) mmHg and limits of agreement of ‐30.99–28.71 mmHg for SBP; and 5.76 (8.29) mmHg and limits of agreement of ‐12.98–24.52 mmHg for DBP (online Supporting Information Figure S1A–C).

Seventeen studies using volume clamp technique with two finger cuffs (CNAP) were evaluated for MAP (849 patients, 1,104,234 measurements); 16 including 839 patients were evaluated for SBP (1,103,107 measurements) and DBP (1,104,085 measurements), respectively. A pooled bias (SD) of 3.89 (9.65) mmHg and limits of agreement of ‐16.37–24.15 mmHg were shown for MAP; ‐1.95 (14.20) mmHg and limits of agreement of ‐31.19–27.29 mmHg for SBP; and 6.34 (9.48) mmHg and limits of agreement of ‐13.87–26.55 mmHg for DBP (online Supporting Information Figure S2A–C).

Nine studies using arterial applanation tonometry (T‐Line) included 215 patients (273,204 measurements) for evaluation of MAP; eight were evaluated for SBP and DBP (201 patients, 245,304 measurements). A pooled bias (SD) of 2.53 (6.60) mmHg and limits of agreement of ‐10.92–15.97 mmHg were shown for MAP; ‐0.07 (9.15) mmHg and limits of agreement of ‐18.65–18.78 mmHg for SBP; and 3.09 (7.17) mmHg and limits of agreement of ‐11.25–17.44 for DBP (online Supporting Information Figure S3A–C).

Four studies using applanation tonometry (Vasotrac) were evaluated for MAP (131 patients, 64,654 measurements); three were evaluated for SBP and DBP (116 patients, 64,347 measurements). A pooled bias (SD) of 0.36 (5.97) mmHg and limits of agreement of ‐11.73–12.44 mmHg were shown for MAP; 1.20 (10.22) mmHg and limits of agreement of ‐20.28–22.68 mmHg for SBP; and 1.09 (7.01) mmHg and limits of agreement of ‐13.35–15.53 mmHg for DBP (Figure S4A–C).

Four studies using arterial applanation tonometry (N‐CAT) were evaluated for MAP (48 patients, 89,558 measurements); three were evaluated for SBP and DBP (32 patients, 1400 measurements). A pooled bias (SD) of ‐0.37 (6.97) mmHg and limits of agreement of ‐14.73–13.98 mmHg were shown for MAP; ‐3.68 (7.14) mmHg and limits of agreement of ‐18.23–10.88 mmHg for SBP; and 0.84 (6.55) mmHg and limits of agreement of ‐13.45–15.13 mmHg for DBP (online Supporting Information Figure S4D–F), respectively.

Six studies using photoplethysmography, using non‐commercially available measurement techniques [24, 97, 98] and commercially available measuring devices (OptiBP [35], Aktiia OBPM [36] and NICAP technology [99]) were included for meta‐analyses. Five studies were evaluated for MAP (117 patients, 15,631 measurements), seven for SBP (159 patients, 16,273 measurements) and eight for DBP (179 patients, 16,435 measurements). A pooled bias (SD) of ‐0.09 (6.86) mmHg and limits of agreement of ‐13.80–13.63 mmHg were shown for MAP; 0.49 (9.63) mmHg and limits of agreement of ‐18.78–19.76 mmHg for SBP; and ‐0.26 (6.38) mmHg and limits of agreement of ‐13.02–12.50 mmHg for DBP (online Supporting Information Figure S5A–C), respectively. A graphical summary of the pooled results is shown in Figure 2.

**Figure 2:**
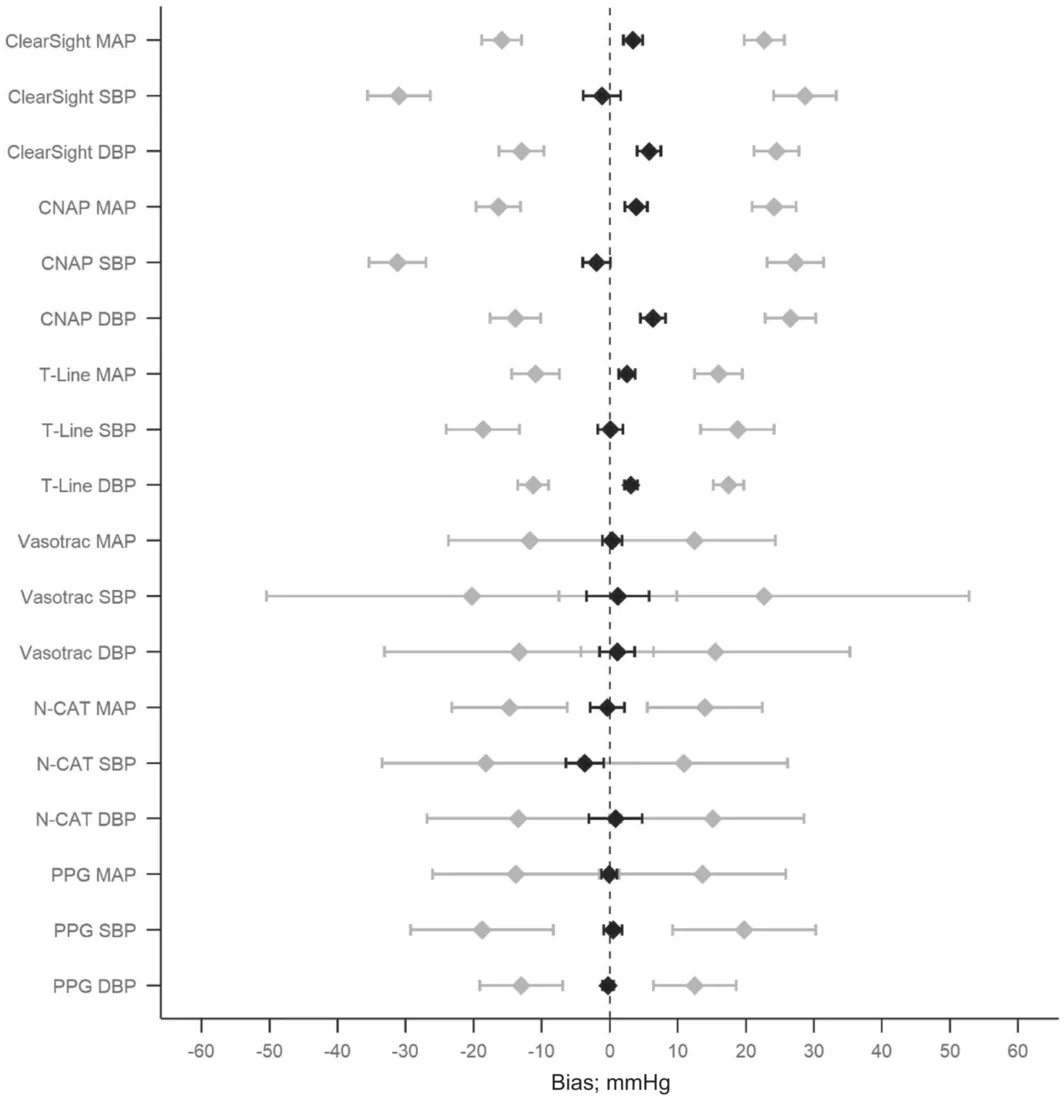
Pooled results. Black datapoints, mean (95%CI) bias; grey, mean (95%CI) of the lower and upper limits of agreement. MAP, mean arterial pressure; SBP, systolic blood pressure; DBP, diastolic blood pressure; PPG, photoplethysmography.

Influence diagnostics did not detect influential studies in six analyses (CNAP, MAP and SBP; T‐Line, MAP and DBP; photoplethysmography, MAP and DBP). In the other analyses there were 1–3 studies flagged as influential. If influential studies were detected, we performed a sensitivity analysis on bias excluding the influential studies. The absolute difference in pooled bias between the full model and the reduced model was generally < 1.2 mmHg; for Vasotrac DBP the difference was 1.31 mmHg and for N‐CAT SBP it was 1.82 mmHg. Taken together, there were minor quantitative differences between the full and reduced models that do not affect interpretation of the results (online Supporting Information Table S8).

To assess possible clustering of studies of the same working group, we performed a robust analysis for the bias with first author (as a proxy for working group) as cluster indicator. Robust analysis was not possible for meta‐analyses which only included a small number of studies, in particular, when most studies included in one meta‐analysis were published by the same working group. When robust estimates were obtained, the length of the confidence intervals for bias increased by 4% to 44% for most analyses in comparison with the length of the confidence intervals obtained using the standard DerSimoian Laird method, indicating limited clustering. However, for the measurements of SBP and DBP with Vasotrac, where only three studies were included in the meta‐analyses, the length of the confidence intervals more than doubled, pointing to more substantial clustering. No formal comparison was feasible for N‐CAT as these rely on only three studies by the same authors, two of which were in the same paper. Comparison was neither feasible for MAP measurement with photoplethysmography technology because again there were only three different authors reporting five studies in three papers. Details are given in the online Supporting Information Table S9.

Bias assessment according to modified QUADAS‐2 showed the following prevalence of low‐risk studies: 4/28 studies for volume clamp technology with one finger cuff (ClearSight); 2/15 studies for volume clamp technology with two finger cuffs (CNAP); 1/16 studies for applanation tonometry; and 0/6 for studies using photoplethysmography technology. The categorisation of each study as high, low and unclear is shown in online Supporting Information Table S6.

The remaining studies showed an unclear or increased risk of bias due to: missing information (especially on the study design); definition of the initial measurement criteria; treatment of multiple measurements per patient; verification of the assumptions for Bland–Altman analysis; or inconsistent conclusions.

## Discussion

We performed six separate systematic meta‐analyses comparing non‐invasive, continuous blood pressure measurement technologies with invasive radial arterial blood pressure measurement in adult patients in the operating theatre or ICU. Most technologies failed to meet the AAMI criteria for bias and SD and had wide limits of agreement. Only the N‐CAT applanation tonometer showed acceptable accuracy.

A previous systematic review and meta‐analysis of continuous non‐invasive blood pressure measurement devices published in 2014 [14] reported that accuracy and precision of these devices compared with invasive pressure monitoring could not be considered acceptable according to AAMI criteria [29, 30]. Another systematic review and meta‐analysis reported wide limits of agreement regarding MAP, SBP and DBP vs. arterial‐line pressure monitoring [15]. In a subgroup analysis of arterial applanation tonometry, the T‐Line system failed to meet AAMI criteria for DBP measurement (mean bias 6.31 mmHg). However, the bias of volume clamp technology with one finger cuff (ClearSight) with respect to DBP measurements showed a better mean bias (3.52 mmHg) in contrast to our results [15]. This difference might be due to a smaller number of studies included in their analysis. Regarding volume clamp technology, the pooled data of a recent meta‐analysis illustrated that invasive blood pressure measurement was not interchangeable with finger cuff technologies [106]. For volume clamp technology with one finger cuff (ClearSight), mean bias for MAP was 4.92 mmHg, 0.0 mmHg for SBP and 7.79 mmHg for DBP. With two finger cuffs (CNAP), mean bias for MAP was 3.74 mmHg, ‐2.67 mmHg for SBP and 4.81 mmHg for DBP [106]. Moreover, the authors criticised the high heterogeneity of the studies included.

All previous meta‐analyses, along with the studies included in the meta‐analyses presented here, applied the AAMI guidelines consistently [29, 30] with an acceptable bias of ≤ 5 mmHg and an SD ≤ 8 mmHg. Based on this approach, we considered the difference between the upper and lower limits of agreement acceptable if it did not exceed 32 mmHg. However, this guideline was developed for intermittent, non‐invasive blood pressure measurement [29, 30].

A new guideline which addresses recommendations for continuous blood pressure devices according to ISO 81060‐3:2022 was released in 2022 [107]. Following this guideline with a wider acceptable zone (bias of ≤6 mmHg and SD ≤10 mmHg), ClearSight would fulfil the criteria for MAP and DBP as well as CNAP for MAP. Nonetheless, to ensure comparability with the included studies and previous meta‐analyses, the AAMI criteria from 2018 [29] and 2020 [30] were used in this study, as recommended previously [108, 109]; this may be considered a limitation of the study. Besides publication and language bias, population selection bias can also be considered a further limitation, as only studies with invasive reference blood pressure measurement in the radial artery were included.

In conclusion, these meta‐analyses evaluate accuracy and precision of various continuous, non‐invasive arterial blood pressure measurement technologies, compared with invasive radial arterial blood pressure measurement. There was considerable heterogeneity between the studies included in the meta‐analyses, both with respect to bias and limits of agreement. Heterogeneity was particularly pronounced between the studies addressing ClearSight and CNAP. Given the wide ranges between the lower and upper limits of agreement, the use of all non‐invasive continuous blood pressure measurement devices should only be performed in suitable patients and with consideration of possible wide limits of agreement. In a personalised approach of haemodynamic management and individualised care, treatment decisions based solely on non‐invasive continuous blood pressure values should be made with great caution. Clinicians should be aware of the lack of interchangeability with invasive blood pressure management if they choose to use these devices. Obvious limitations of these devices should be kept in mind while considering potential benefits vs. costs. New emerging technologies for continuous non‐invasive arterial blood pressure measurement warrant further studies in a variety of clinical settings and populations. An international consensus on essential criteria would strengthen the comparability and interpretation of studies.

## Supporting information


**Appendix S1.** Search process according to PRISMA guidelines.

**Appendix S2.** Statistical code.

**Figure S1.** Forest plot: volume clamp technology (ClearSight).
**Figure S2.** Forest plot: volume clamp technology (CNAP).
**Figure S3.** Forest plot: applanation tonometry (T‐Line).
**Figure S4.** Forest plot: applanation tonometry (Vasotrac and N‐CAT).
**Figure S5.** Forest plot: photoplethysmography technology.

**Tables S1–S5.** Overview of all included studies.
**Table S6.** Risk of bias assessment.
**Table S7.** Meta‐analyses summary table.
**Table S8.** Summary parameters for meta‐analysis focusing on bias difference.
**Table S9.** Comparison of summary parameters for bias focusing on ratio of confidence interval length.
