# Supplementary material for: Accuracy and precision of continuous, non‐invasive blood pressure devices: a systematic review and meta‐analyses
Source: Anaesthesia. 2026 Jun 15;81(11):1581–92. doi: 10.1111/anae.70250 (PMC13659784; doi:10.1111/anae.70250)
Supplement: Supplementary file 1 [file ANAE-81-1581-s001.pdf]

Figure S1: Results presented as forest plot: Volume clamp technology (ClearSight™)

A

Study (ClearSight MAP)	Weight	$n_{subjects}$	$n_{measurements}$	Bias [95% CI]
Balzer, 2016	2.4%	20	100	1.00 [-4.70, 6.70]
Berkelmans, 2018 T1	3.5%	31	4650	0.00 [-2.82, 2.82]
Berkelmans, 2018 T2	3.4%	10	1500	1.00 [-2.10, 4.10]
Broch, 2013, T1	3.2%	50	278	10.90 [7.30, 14.50]
Broch, 2013, T2	* 3.3%	50	236	13.50 [10.37, 16.63]
Bugarini, 2021	2.0%	24	1903	-0.81 [-7.96, 6.34]
Chachula, 2020, T1	1.3%	10	63	1.60 [-8.32, 11.52]
Chachula, 2020, T2	1.4%	10	41	2.80 [-6.68, 12.28]
Chachula, 2020, T3	1.4%	10	104	2.60 [-7.13, 12.33]
Eley, 2021	3.1%	30	2229	-5.20 [-9.10, -1.30]
Fassaert, 2021	3.6%	48	36206	4.00 [1.79, 6.21]
Fischer, 2012	3.7%	44	220	4.60 [2.68, 6.52]
Frank, 2020	3.4%	52	1404	5.47 [2.46, 8.48]
Hansen, 2022	3.5%	56	801	-1.43 [-4.05, 1.19]
Heusdens, 2016	3.7%	24	3782	3.50 [1.42, 5.58]
Hofhuizen, 2014	3.4%	20	54	4.20 [1.19, 7.21]
Kho, 2022 T1	3.8%	33	22308	4.50 [2.93, 6.07]
Kho, 2022 T2	3.7%	33	22308	-1.10 [-3.15, 0.95]
Lee, 2021	3.1%	26	8408	1.05 [-2.77, 4.87]
Lu, 2021	3.3%	20	2243	-0.30 [-3.54, 2.94]
Martina, 2012	3.8%	50	9000	2.20 [0.43, 3.97]
Misugi, 2022	3.2%	21	1277	2.70 [-0.72, 6.12]
Noto, 2019	3.6%	30	2672	6.80 [4.40, 9.20]
Omenås, 2020	3.6%	23	209	4.30 [1.92, 6.68]
Rogge, 2019	3.6%	35	95963	-1.10 [-3.55, 1.35]
Sakai, 2018	1.9%	10	210	9.26 [2.06, 16.46]
Schramm, 2017	* 2.5%	35	280	22.67 [17.30, 28.04]
Schumann, 2020	3.6%	90	538	-1.00 [-3.27, 1.27]
Tanioku, 2020	3.2%	18	3068	3.90 [0.30, 7.50]
Vos, 2014	3.8%	110	758	2.00 [0.32, 3.68]
Wilde, 2016	3.6%	19	57	0.82 [-1.44, 3.08]
Yokose, 2016	3.5%	30	6312	7.90 [5.14, 10.66]
Random-Effects Model				3.40 [2.00, 4.80]

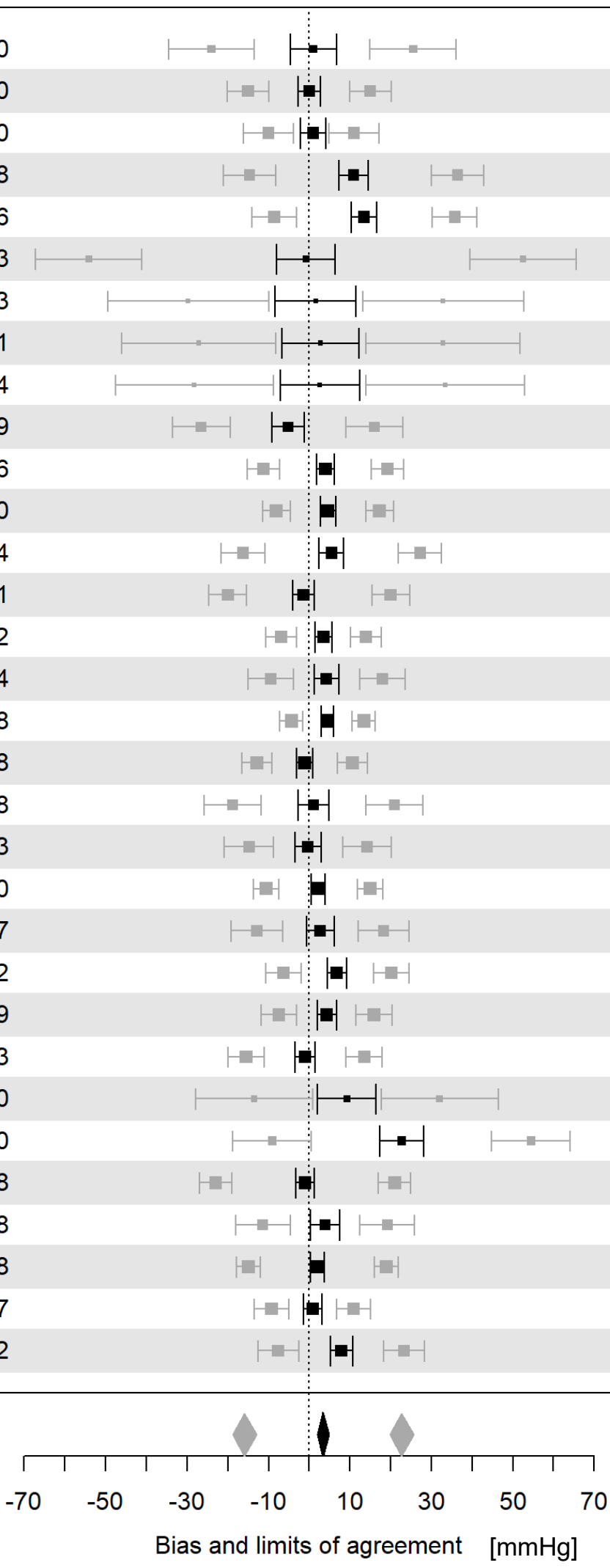

Heterogeneity: $Tau^2 = 12.72$; $Chi^2 = 233.85$, df = 31 (P < 0.01); $I^2 = 87\%$

n number; *MAP* mean arterial pressure; *CI* confidence interval

B

Study (ClearSight SBP)	Weight	$n_{subjects}$	$n_{measurements}$	Bias [95% CI]
Balzer, 2016	3.2%	20	100	-5.00 [-12.01, 2.01]
Berkelmans, 2018 T1	3.7%	31	4650	-4.00 [-8.22, 0.22]
Berkelmans, 2018 T2	3.4%	10	1500	-6.00 [-12.20, 0.20]
Broch, 2013, T1	3.6%	50	278	11.40 [6.66, 16.14]
Broch, 2013, T2	3.6%	50	236	15.10 [10.14, 20.06]
Bugarini, 2021	2.9%	24	1903	10.22 [1.63, 18.81]
Eley, 2021	3.6%	30	2229	-14.30 [-19.35, -9.25]
Fassaert, 2021	3.8%	48	36206	-9.58 [-13.43, -5.73]
Fischer, 2012	3.7%	44	220	-5.70 [-9.87, -1.53]
Frank, 2020	* 3.7%	52	1404	18.05 [13.49, 22.61]
Heusdens, 2016	3.7%	24	3782	-3.30 [-7.62, 1.02]
Hofhuizen, 2014	3.6%	20	54	2.70 [-2.16, 7.56]
Kho, 2022 T1	3.9%	33	22308	-2.70 [-5.87, 0.47]
Kho, 2022 T2	3.8%	33	22308	-1.90 [-5.52, 1.72]
Lee, 2021	3.4%	26	8408	-5.18 [-11.45, 1.09]
Lu, 2021	3.7%	20	2243	-9.80 [-14.23, -5.37]
Martina, 2012	4.0%	50	9000	-0.50 [-2.36, 1.36]
Misugi, 2022	3.5%	21	1277	-0.80 [-6.53, 4.93]
Noto, 2019	3.8%	30	2672	3.00 [-0.47, 6.47]
Omenås, 2020	3.6%	23	209	-12.20 [-16.97, -7.43]
Rogge, 2019	3.8%	35	95963	-6.80 [-10.21, -3.39]
Sakai, 2018	2.7%	10	210	2.99 [-6.80, 12.78]
Schramm, 2017	3.4%	35	280	14.14 [7.82, 20.46]
Schumann, 2020	3.9%	90	538	-7.00 [-9.89, -4.11]
Tanioku, 2020	3.0%	18	3068	-13.20 [-21.28, -5.12]
Weiss, 2014	3.5%	31	3479	3.80 [-1.88, 9.48]
Wilde, 2016	3.8%	19	57	-3.30 [-6.82, 0.22]
Yokose, 2016	3.7%	30	6312	0.70 [-3.83, 5.23]
Random-Effects Model				-1.14 [-3.88, 1.59]

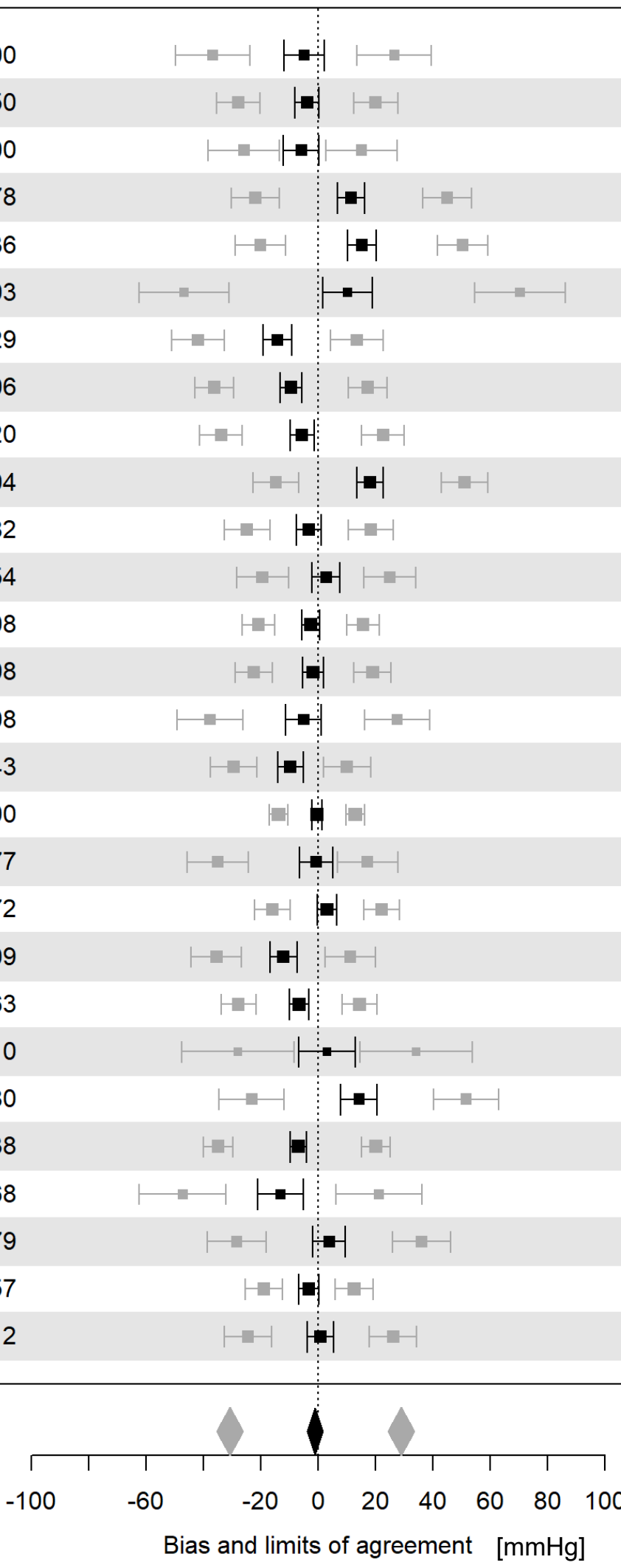

Heterogeneity: $Tau^2 = 47.65$; $Chi^2 = 305.45$, df = 27 ($P < 0.01$); $I^2 = 91\%$

n number; *SBP* systolic blood pressure; *CI* confidence interval

C

Study (ClearSight DBP)	Weight	$n_{subjects}$	$n_{measurements}$	Bias [95% CI]
Balzer, 2016	3.1%	20	100	5.00 [-0.26, 10.26]
Berkelmans, 2018 T1	3.9%	31	4650	1.00 [-1.46, 3.46]
Berkelmans, 2018 T2	3.6%	10	1500	4.00 [0.28, 7.72]
Broch, 2013, T1	3.6%	50	278	13.00 [9.26, 16.74]
Broch, 2013, T2	3.7%	50	236	13.70 [10.35, 17.05]
Bugarini, 2021	2.9%	24	1903	-5.38 [-11.24, 0.48]
Eley, 2021	3.5%	30	2229	-2.60 [-6.46, 1.26]
Fassaert, 2021	4.0%	48	36206	7.92 [5.88, 9.96]
Fischer, 2012	4.0%	44	220	8.90 [6.88, 10.92]
Frank, 2020	3.8%	52	1404	1.09 [-1.94, 4.12]
Heusdens, 2016	4.0%	24	3782	6.10 [3.82, 8.38]
Hofhuizen, 2014	3.8%	20	54	4.90 [1.92, 7.88]
Kho, 2022 T1	4.1%	33	22308	6.40 [4.93, 7.87]
Kho, 2022 T2	4.0%	33	22308	-0.70 [-2.75, 1.35]
Lee, 2021	3.7%	26	8408	5.16 [1.78, 8.54]
Lu, 2021	3.7%	20	2243	5.90 [2.48, 9.32]
Martina, 2012	4.1%	50	9000	2.80 [1.03, 4.57]
Misugi, 2022	3.8%	21	1277	6.30 [3.26, 9.34]
Noto, 2019	4.0%	30	2672	9.00 [7.07, 10.93]
Rogge, 2019	4.0%	35	95963	-0.80 [-3.09, 1.49]
Sakai, 2018	2.6%	10	210	12.03 [5.31, 18.75]
Schramm, 2017	* 3.2%	35	280	24.69 [19.71, 29.67]
Schumann, 2020	4.0%	90	538	0.00 [-2.27, 2.27]
Tanioku, 2020	3.7%	18	3068	9.10 [5.73, 12.47]
Weiss, 2014	3.6%	31	3479	8.80 [5.09, 12.51]
Wilde, 2016	4.0%	19	57	2.42 [0.53, 4.31]
Yokose, 2016	3.9%	30	6312	10.10 [7.61, 12.59]
Random-Effects Model				5.76 [4.00, 7.53]

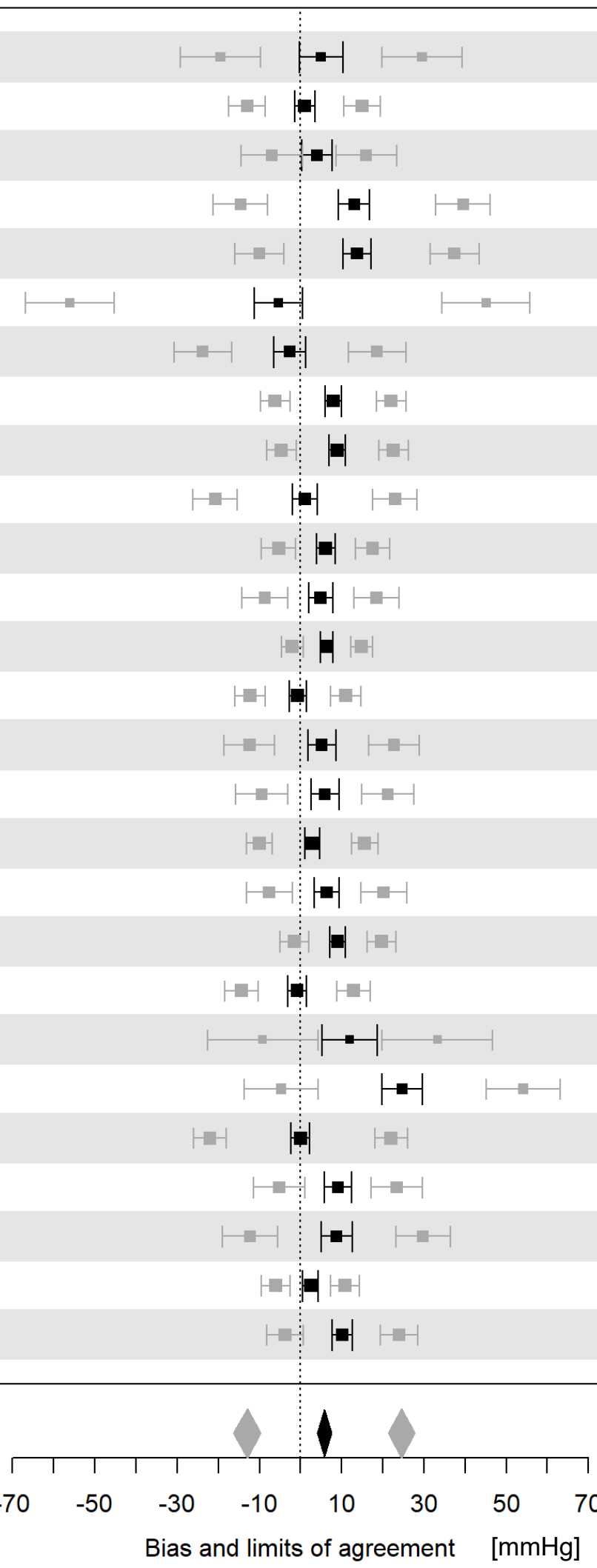

Heterogeneity: $Tau^2 = 19.10$; $Chi^2 = 307.03$, df = 26 ($P < 0.01$); $I^2 = 92\%$

n number; *DBP* diastolic blood pressure; *CI* confidence interval

Figure S2: Results presented as forest plot: Volume clamp technology (CNAP™)

A

**Study (CNAP MAP)**	Weight	$n_{subjects}$	$n_{measurements}$	**Bias [95% CI]**
Araz, 2015	5.8%	40	5433	-0.40 [-3.62, 2.82]
Biais, 2019	5.2%	25	1452	-1.80 [-5.84, 2.24]
Flick, 2021	5.9%	49	276907	9.00 [5.86, 12.14]
Gayat, 2013	5.5%	52	5174	8.00 [4.40, 11.60]
Hahn, 2012, T1	6.1%	50	249508	2.90 [-0.04, 5.84]
Hahn, 2012, T2	6.3%	50	237562	3.10 [0.48, 5.72]
Huhle, 2016	4.1%	10	100	-2.80 [-8.32, 2.72]
Ilies, 2012, T1	6.5%	85	4093	10.21 [7.81, 12.61]
Ilies, 2012, T2	7.1%	85	9353	4.34 [2.89, 5.79]
Ilies, 2015	7.1%	104	11222	6.10 [4.64, 7.56]
Jeleazcov, 2010	6.4%	78	156000	1.60 [-0.84, 4.04]
Kumar, 2015	7.1%	60	1200	-0.02 [-1.46, 1.42]
Rogge, 2018	5.6%	29	6864	7.90 [4.41, 11.39]
Sackl-Pietsch, 2008	4.0%	15	75485	6.65 [0.97, 12.33]
Schramm, 2010	4.1%	29	48691	-0.80 [-6.26, 4.66]
Smolle, 2015	6.7%	40	7200	4.60 [2.52, 6.68]
Wang, 2019	6.5%	48	7990	4.50 [2.10, 6.90]
Random-Effects Model				3.89 [2.23, 5.55]

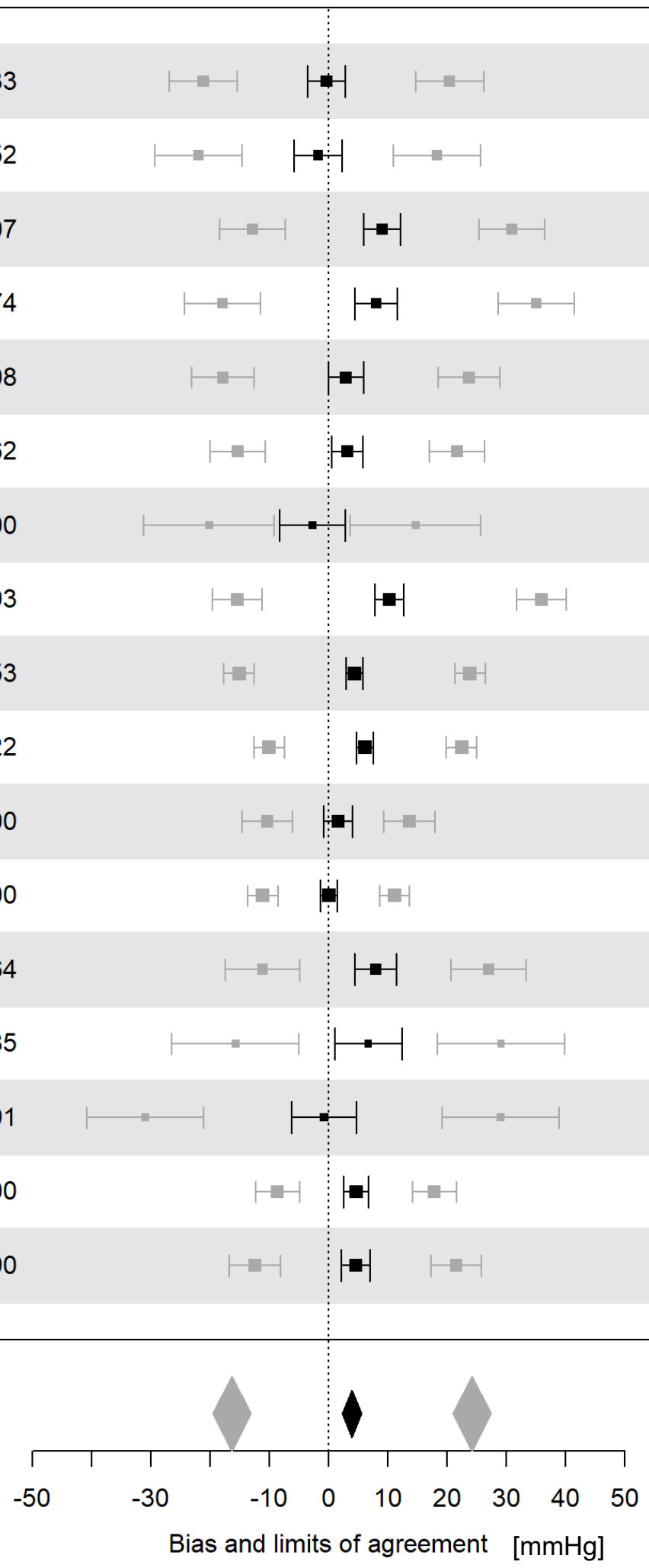

Heterogeneity: $Tau^2 = 9.53$; $Chi^2 = 113.08$, $df = 16$ ($P < 0.01$); $I^2 = 86\%$

n number; *MAP* mean arterial pressure; *CI* confidence interval

B

Study (CNAP SBP)	Weight	$n_{subjects}$	$n_{measurements}$	Bias [95% CI]
Araz, 2015	5.7%	40	5433	0.30 [-4.66, 5.26]
Biais, 2019	5.7%	25	1452	7.20 [2.22, 12.18]
Flick, 2021	6.3%	49	276907	2.50 [-1.70, 6.70]
Gayat, 2013	5.0%	52	5174	2.00 [-3.84, 7.84]
Hahn, 2012, T1	6.1%	50	249508	-3.40 [-7.83, 1.03]
Hahn, 2012, T2	6.7%	50	237562	-0.90 [-4.56, 2.76]
Ilies, 2012, T1	6.9%	85	4093	3.31 [-0.20, 6.82]
Ilies, 2012, T2	7.9%	85	9353	-4.17 [-6.30, -2.04]
Ilies, 2015	7.8%	104	11222	-4.30 [-6.53, -2.07]
Jeleazcov, 2010	7.2%	78	156000	-6.70 [-9.78, -3.62]
Kumar, 2015	7.6%	60	1200	-5.98 [-8.60, -3.36]
Rogge, 2018	5.1%	29	6864	4.80 [-0.95, 10.55]
Sackl-Pietsch, 2008	4.2%	15	75485	-2.96 [-9.95, 4.03]
Schramm, 2010	4.5%	29	47664	-11.00 [-17.55, -4.45]
Smolle, 2015	7.2%	40	7200	-3.20 [-6.33, -0.07]
Wang, 2019	6.1%	48	7990	-6.30 [-10.80, -1.80]
Random-Effects Model				-1.95 [-3.97, 0.06]

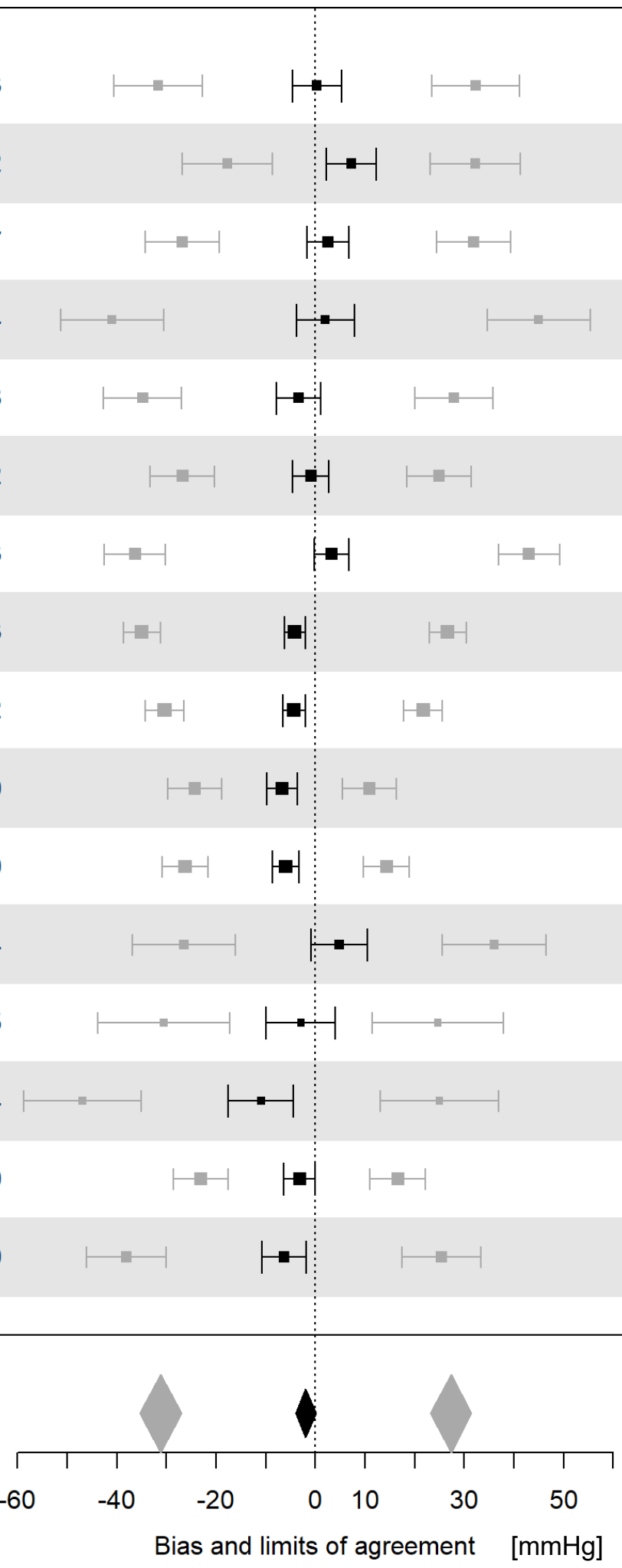

Heterogeneity: $Tau^2 = 12.18$; $Chi^2 = 68.70$, df = 15 ($P < 0.01$); $I^2 = 78\%$

n number; *SBP* systolic blood pressure; *CI* confidence interval

C

Study (CNAP DBP)	Weight	$n_{subjects}$	$n_{measurements}$	Bias [95% CI]
Araz, 2015	6.1%	40	5433	0.40 [-2.88, 3.68]
Biais, 2019	* 5.6%	25	1452	-7.50 [-11.46, -3.54]
Flick, 2021	6.2%	49	276907	10.10 [7.08, 13.12]
Gayat, 2013	6.2%	52	5174	11.00 [7.87, 14.13]
Hahn, 2012	6.3%	50	249508	4.40 [1.41, 7.39]
Hahn, 2012	6.7%	50	237562	2.80 [0.42, 5.18]
Ilies, 2012, T1	6.7%	85	4093	10.78 [8.51, 13.05]
Ilies, 2012, T2	7.2%	85	9353	5.81 [4.53, 7.09]
Ilies, 2015	7.1%	104	11222	9.40 [7.86, 10.94]
Jeleazcov, 2010	6.6%	78	156000	5.60 [3.07, 8.13]
Kumar, 2015	7.1%	60	1200	3.72 [2.18, 5.26]
Rogge, 2018	5.7%	29	6864	9.50 [5.75, 13.25]
Sackl-Pietsch, 2008	4.5%	15	75485	12.36 [6.84, 17.88]
Schramm, 2010	4.3%	29	48642	6.00 [0.18, 11.82]
Smolle, 2015	6.8%	40	7200	7.00 [4.92, 9.08]
Wang, 2019	6.8%	48	7990	9.90 [7.78, 12.02]
Random-Effects Model				6.34 [4.46, 8.22]

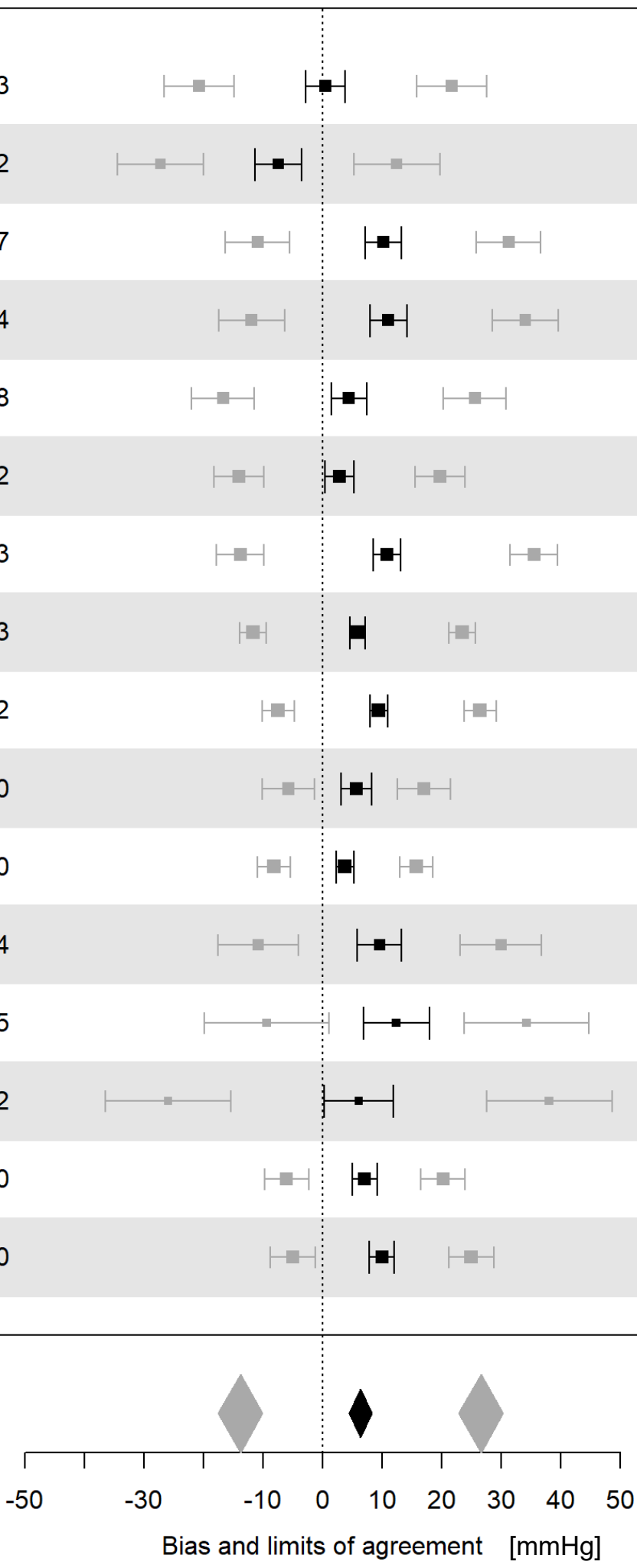

Heterogeneity: $Tau^2 = 12.32$; $Chi^2 = 144.06$, df = 15 ($P < 0.01$); $I^2 = 90\%$

n number; *DBP* diastolic blood pressure; *CI* confidence interval

Figure S3: Results presented as forest plot: Applanation Tonometry (T-Line^{TM})

A

**Study (T-Line MAP)**	Weight	$n_{subjects}$	$n_{measurements}$	**Bias [95% CI]**
Dueck, 2012	10.7%	19	4747	2.30 [-0.35, 4.95]
Greiwe, 2016	7.9%	28	201907	3.97 [0.55, 7.39]
Greiwe, 2018	2.3%	14	27900	16.40 [8.95, 23.85]
Janelle, 2006	13.5%	25	17009	1.70 [-0.38, 3.78]
Langwieser, 2015	13.1%	30	7304	2.00 [-0.15, 4.15]
Lin, 2017	15.6%	23	4381	1.80 [0.08, 3.52]
Meidert, 2014	11.9%	24	2993	2.00 [-0.40, 4.40]
Sun, 2017	12.2%	30	1538	3.10 [0.77, 5.43]
Szmuk, 2008	12.8%	22	5425	1.60 [-0.61, 3.81]
Random-Effects Model				2.53 [1.34, 3.71]

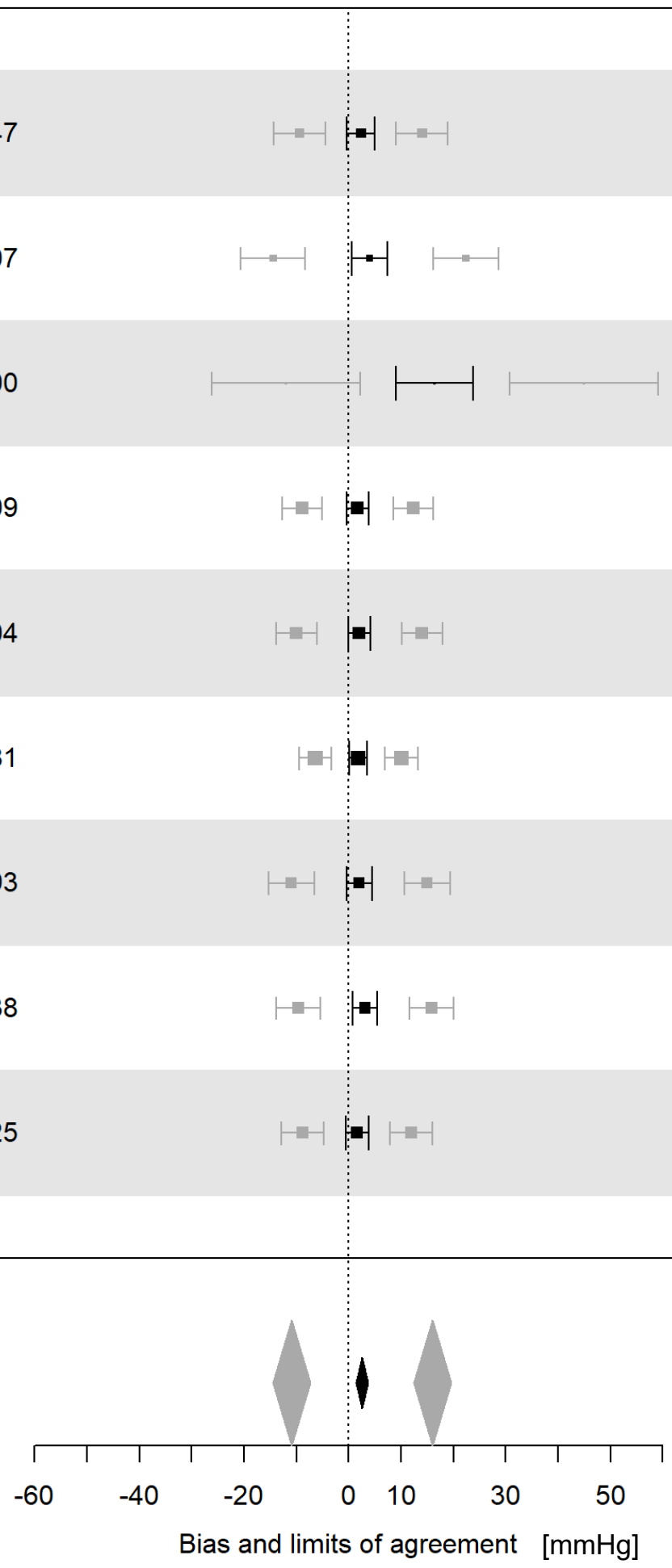

n number; *MAP* mean arterial pressure; *CI* confidence interval

B

Study (T-Line SBP)	Weight	$n_{subjects}$	$n_{measurements}$	Bias [95% CI]
Dueck, 2012	12.6%	19	4747	2.30 [-1.21, 5.81]
Greiwe, 2016	9.2%	28	201907	3.45 [-1.26, 8.16]
Janelle, 2006	15.2%	25	17009	1.70 [-1.04, 4.44]
Langwieser, 2015	* 11.2%	30	7304	-6.00 [-9.94, -2.06]
Lin, 2017	16.5%	23	4381	1.30 [-1.10, 3.70]
Meidert, 2014	6.7%	24	2993	-3.00 [-9.00, 3.00]
Sun, 2017	15.3%	30	1538	-0.90 [-3.62, 1.82]
Szmuk, 2008	13.2%	22	5425	0.00 [-3.30, 3.30]
Random-Effects Model				0.07 [-1.78, 1.91]

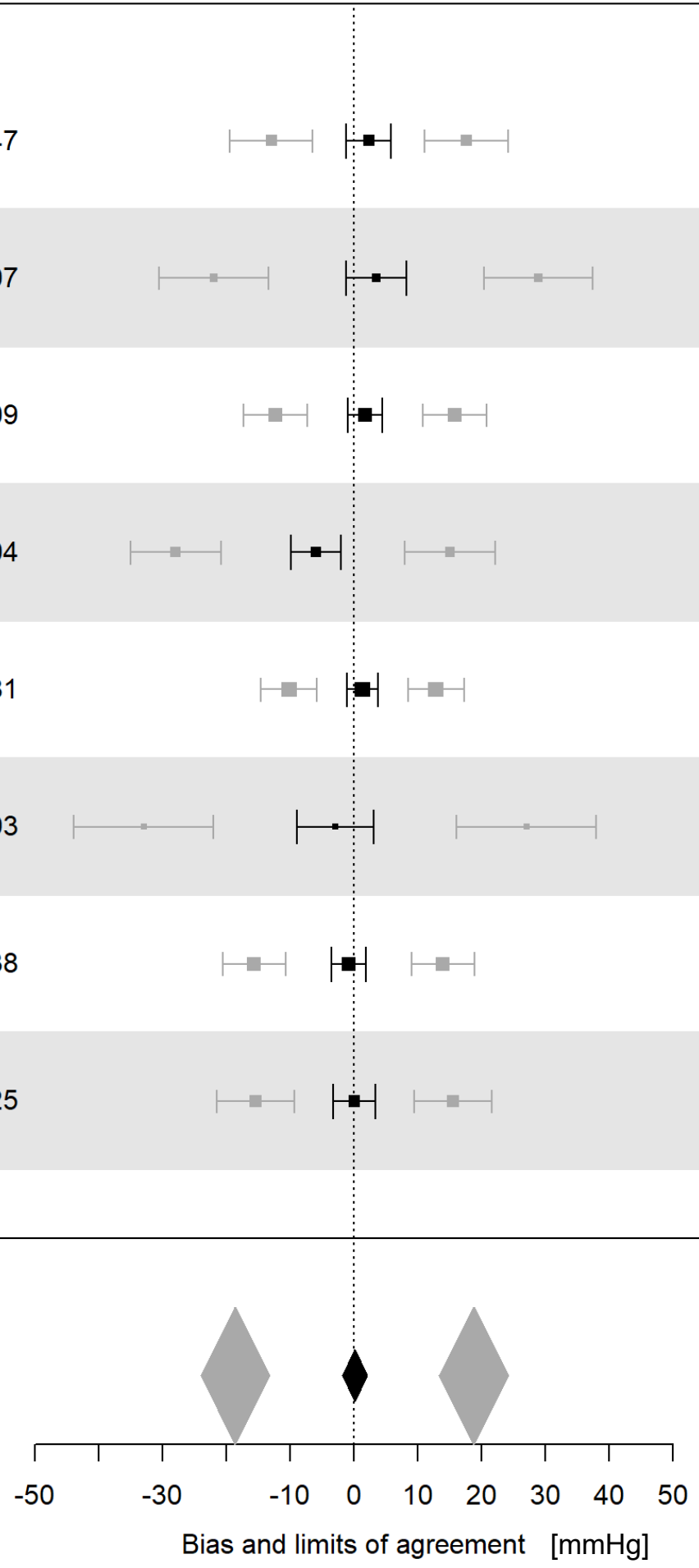

n number; *SBP* systolic blood pressure; *CI* confidence interval

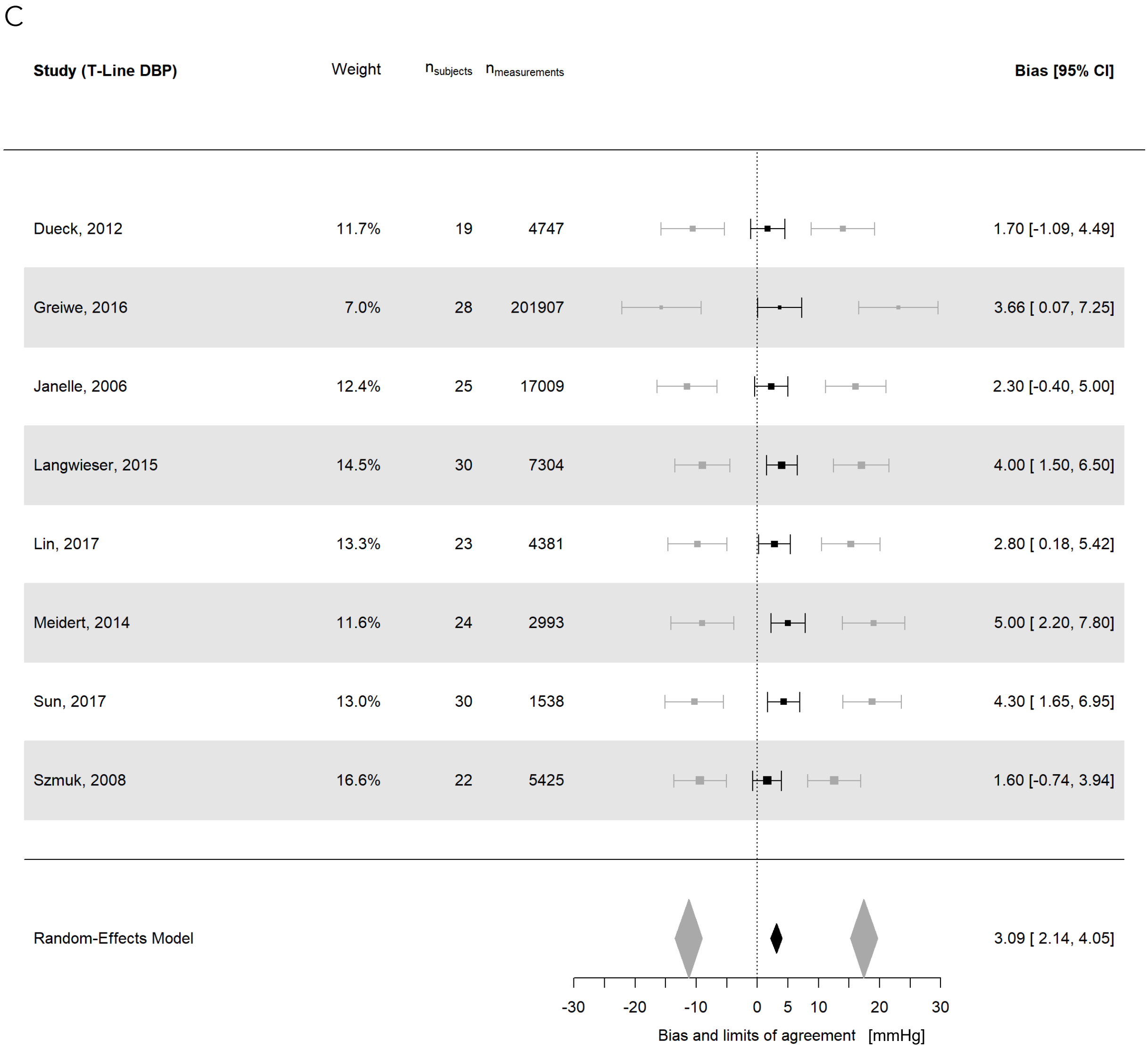

n number; *DBP* diastolic blood pressure; *CI* confidence interval

Figure S4: Results presented as forest plot: Applanation Tonometry (Vasotrac™, and N-CAT™)

A

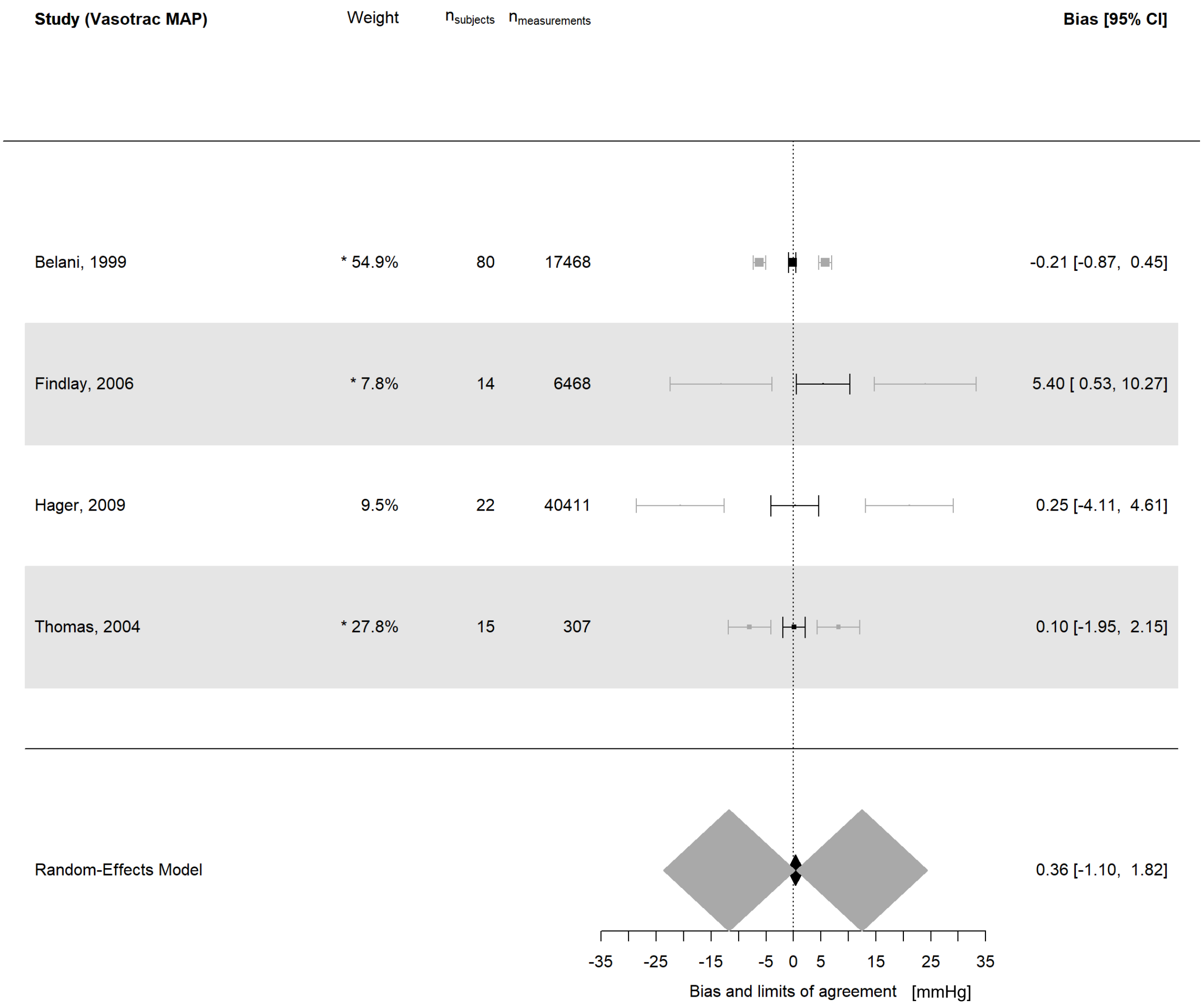

n number; *MAP* mean arterial pressure; *CI* confidence interval

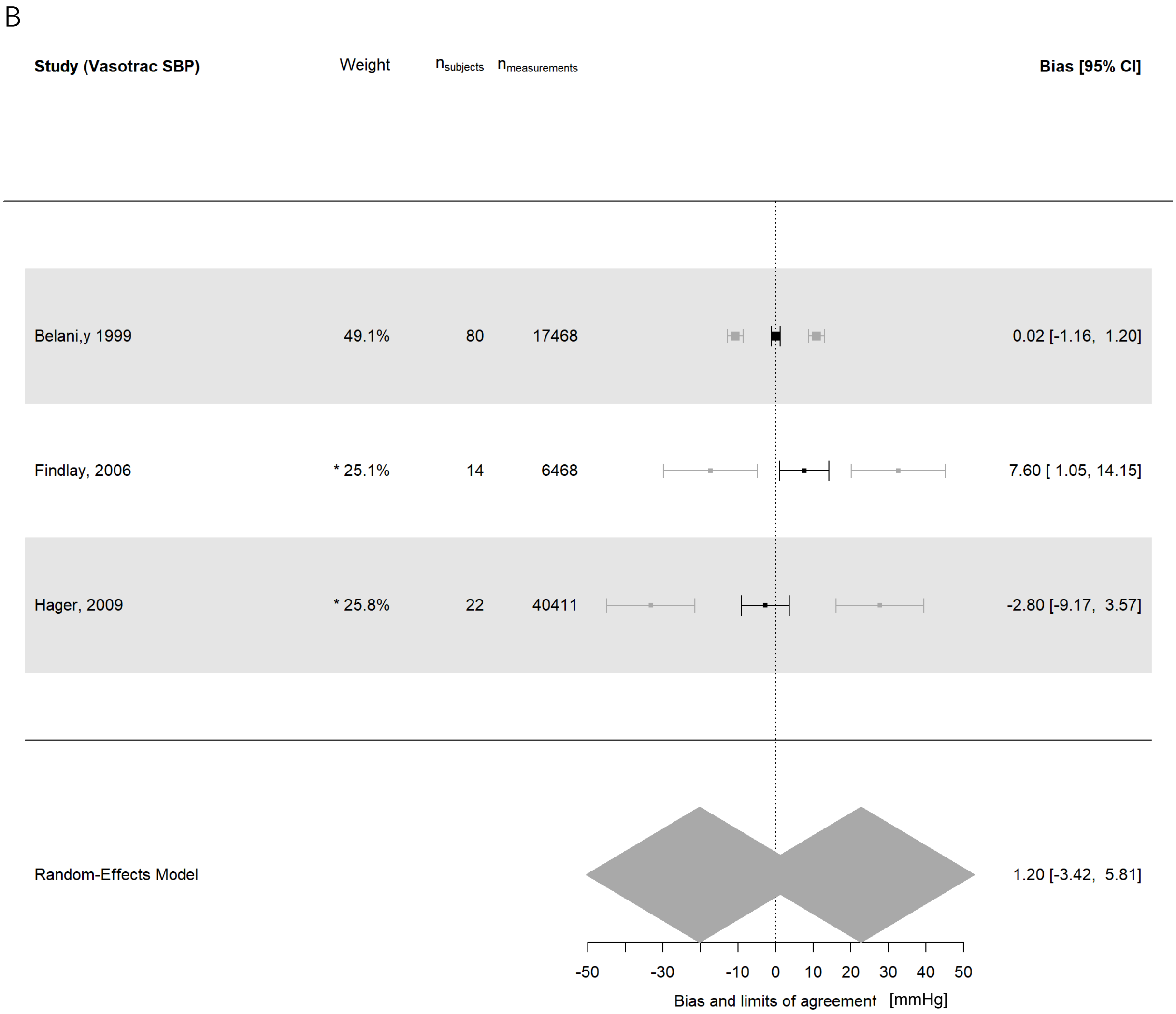

n number; *SBP* systolic blood pressure; *CI* confidence interval

C

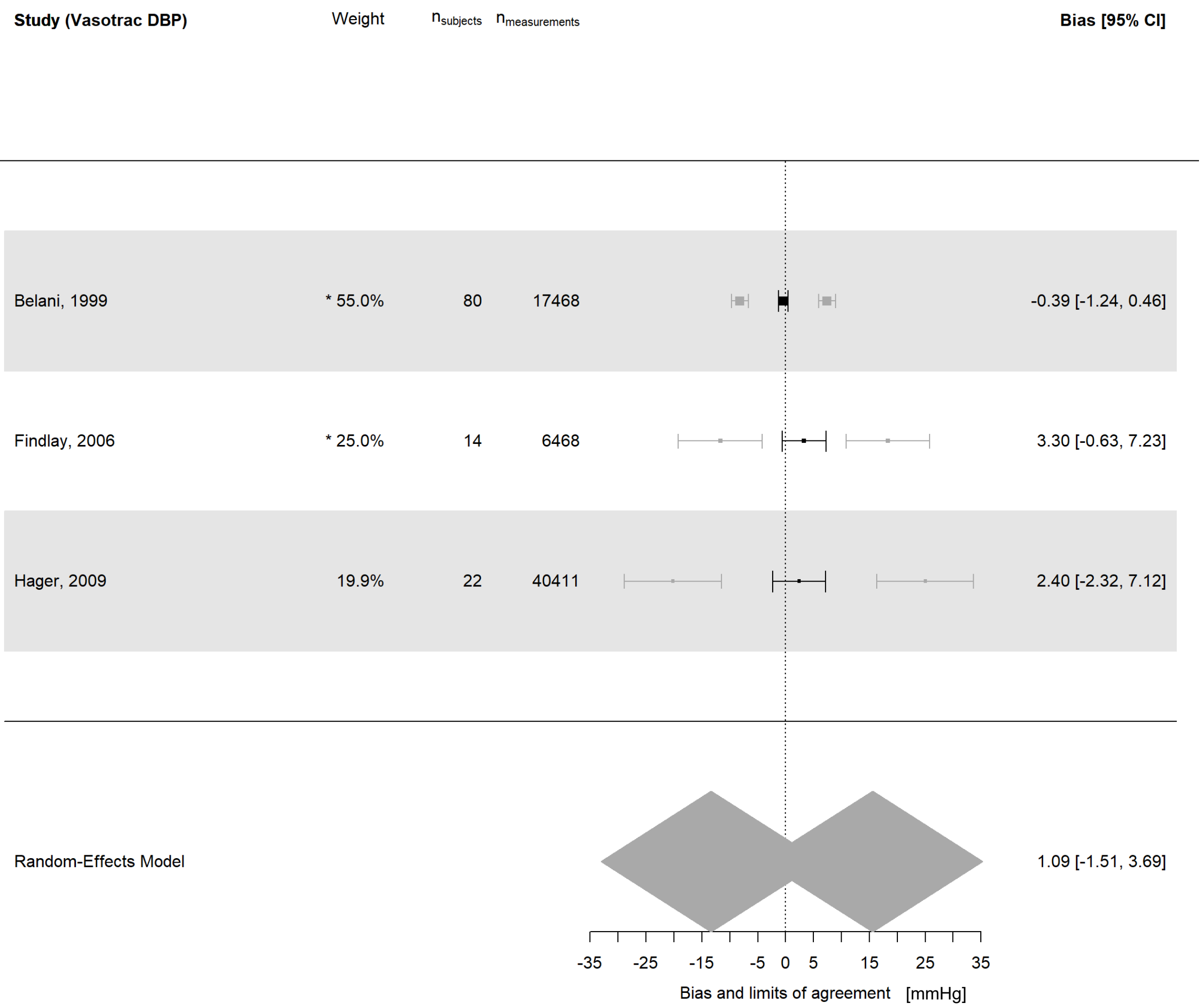

n number; *DBP* diastolic blood pressure; *CI* confidence interval

D

Study (N-CAT MAP)	Weight	$n_{subjects}$	$n_{measurements}$	Bias [95% CI]
Searle, 1993	34.6%	10	300	0.33 [-2.27, 2.93]
Searle, 1995, T1	* 25.4%	11	550	-4.13 [-7.79, -0.47]
Searle, 1995, T2	20.7%	11	550	1.50 [-2.87, 5.87]
Siegel, 1994	19.3%	16	88158	1.30 [-3.31, 5.91]
Random-Effects Model				-0.37 [-2.89, 2.14]

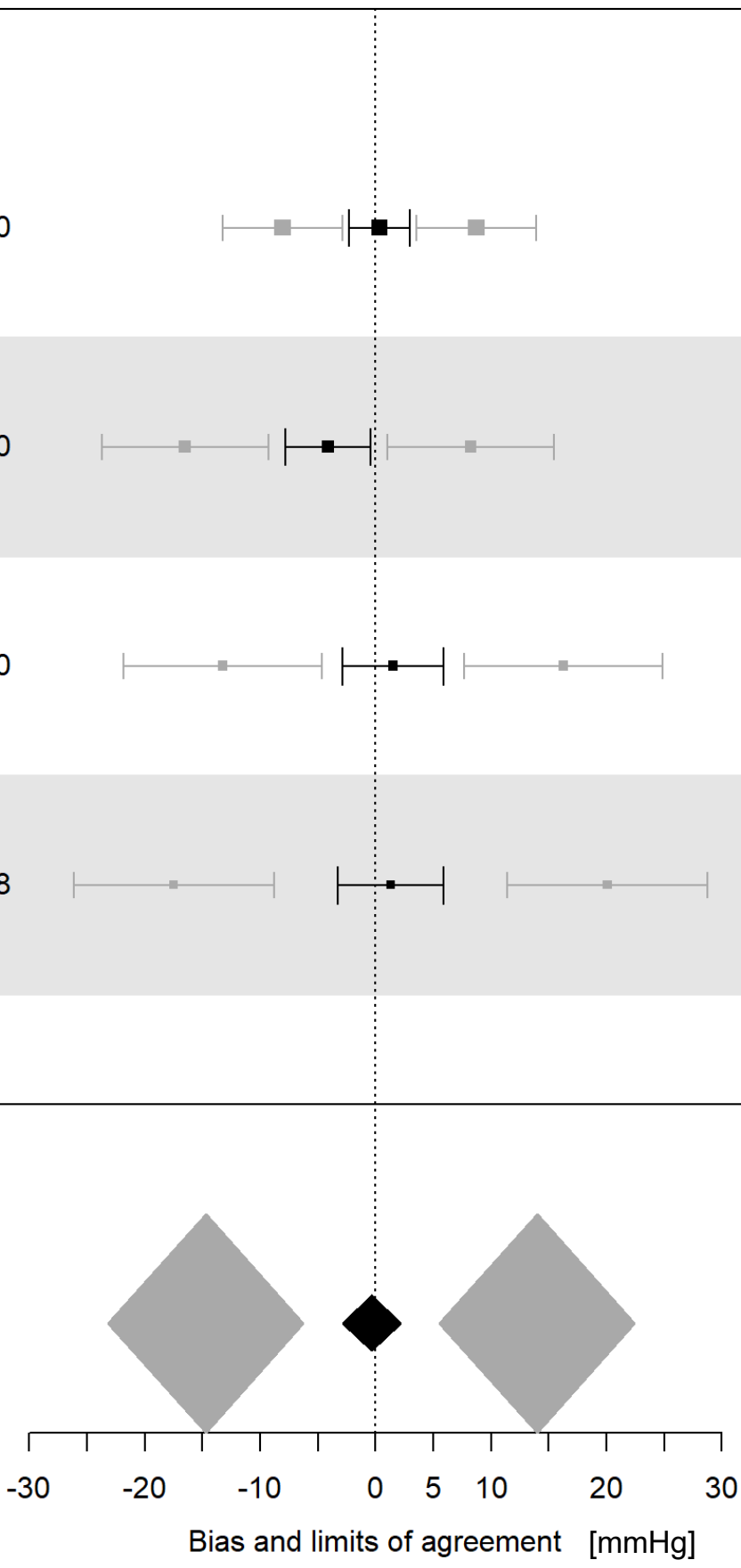

n number; *MAP* mean arterial pressure; *CI* confidence interval

E

Study (N-CAT SBP)	Weight	$n_{subjects}$	$n_{measurements}$	Bias [95% CI]
Searle, 1995, T1	33.9%	11	550	-5.33 [-9.21, -1.45]
Searle, 1995, T2	17.6%	11	550	-5.91 [-11.92, 0.10]
Searle,1993	* 48.5%	10	300	-1.71 [-4.56, 1.14]
Random-Effects Model				-3.68 [-6.46, -0.89]

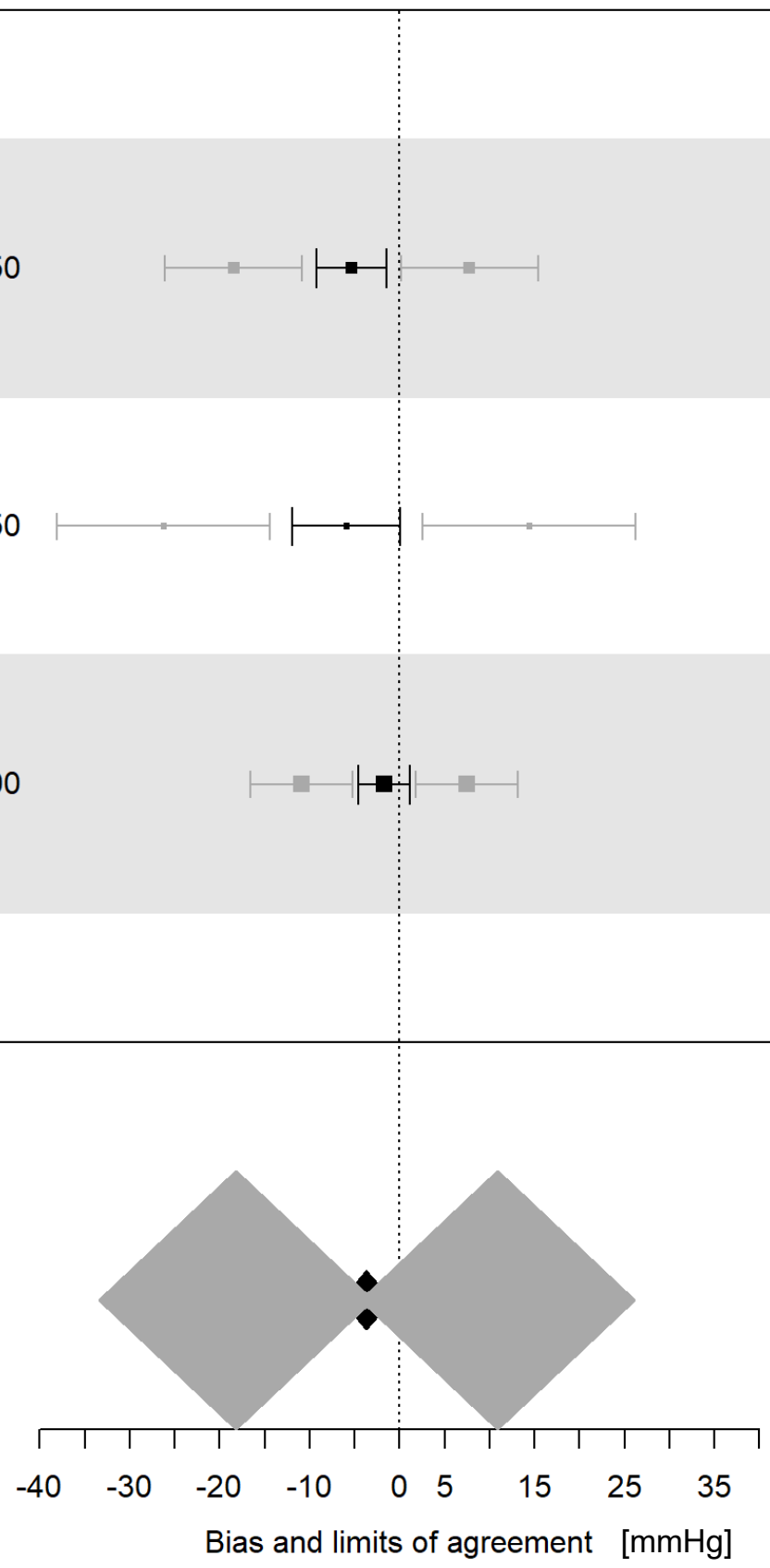

n number; *SBP* systolic blood pressure; *CI* confidence interval

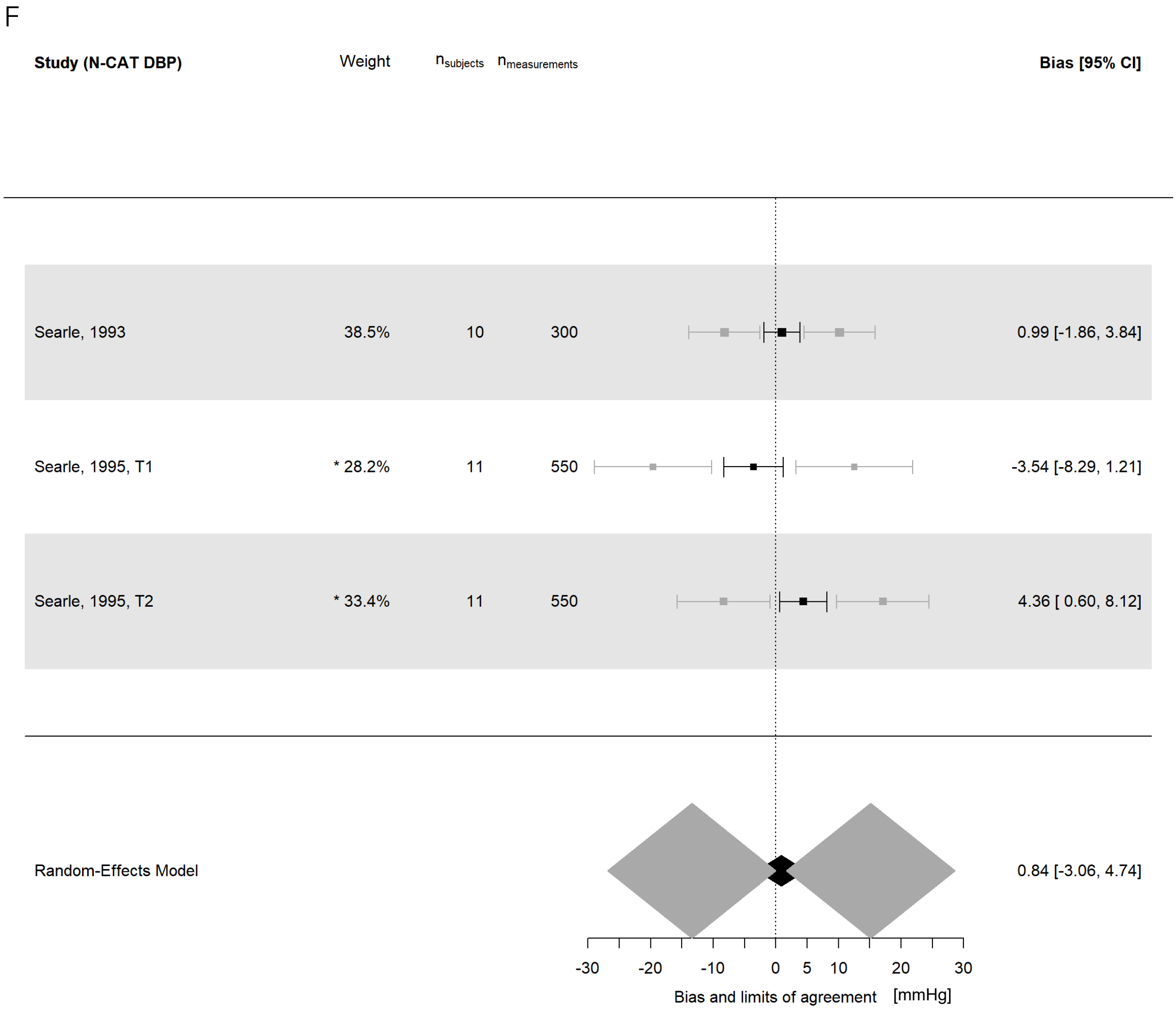

n number; *DBP* diastolic blood pressure; *CI* confidence interval

Figure S5: Results presented as forest plot: PPG-Technology

A

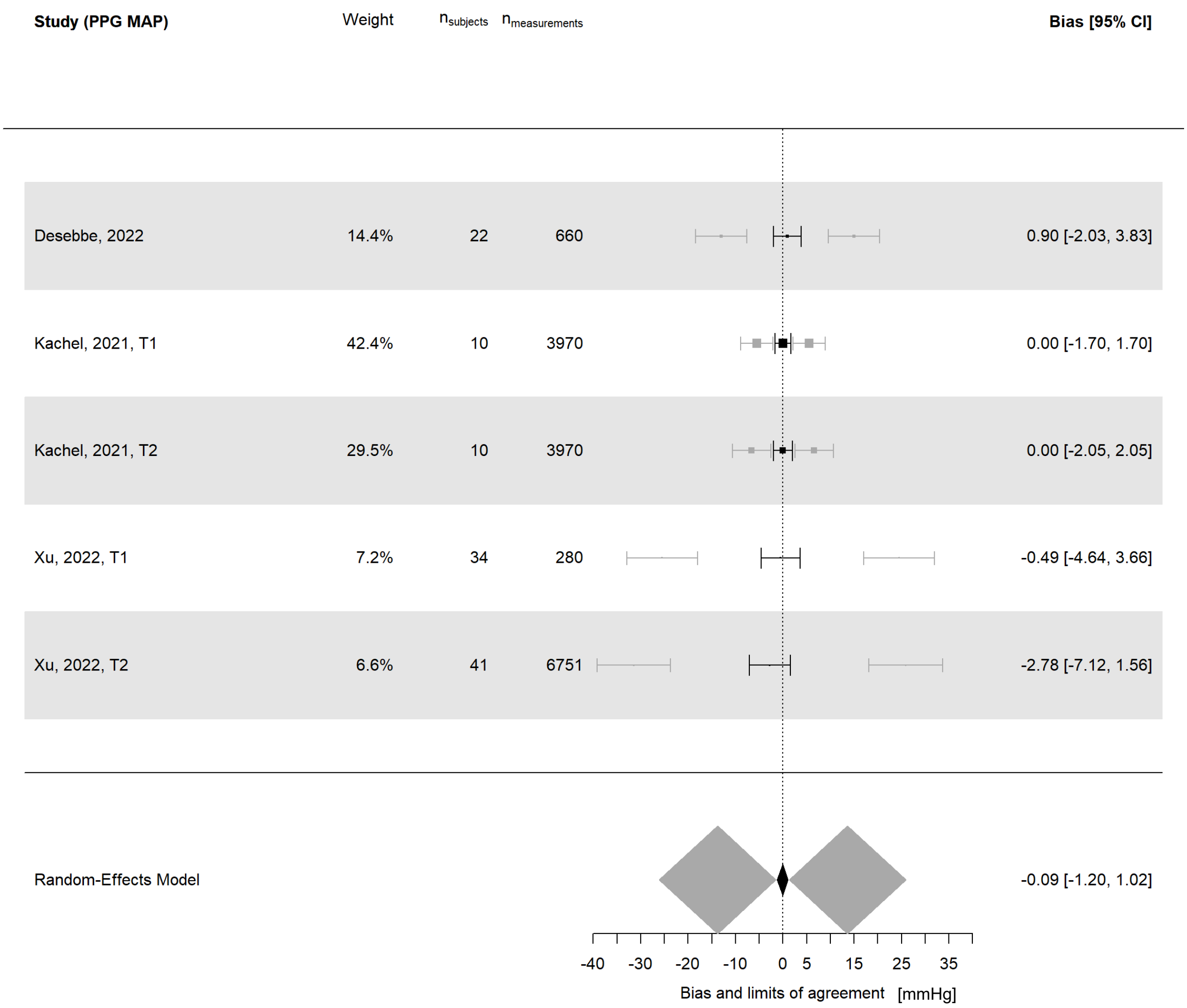

n number; *MAP* mean arterial pressure; *CI* confidence interval

B

Study (PPG SBP)	Weight	$n_{subjects}$	$n_{measurements}$	Bias [95% CI]
Chen, 2012	* 31.7%	26	260	2.16 [-0.23, 4.55]
Desebbe, 2022	5.3%	22	660	0.20 [-5.65, 6.05]
Kachel, 2021, T1	18.5%	10	3998	0.00 [-3.13, 3.13]
Kachel, 2021, T2	19.3%	10	3998	0.00 [-3.07, 3.07]
Pellaton, 2019	15.0%	16	326	0.00 [-3.48, 3.48]
Xu, 2022, T1	4.8%	34	280	-1.72 [-7.87, 4.43]
Xu, 2022, T2	5.4%	41	6751	-2.19 [-7.96, 3.58]
Random-Effects Model				0.49 [-0.85, 1.84]

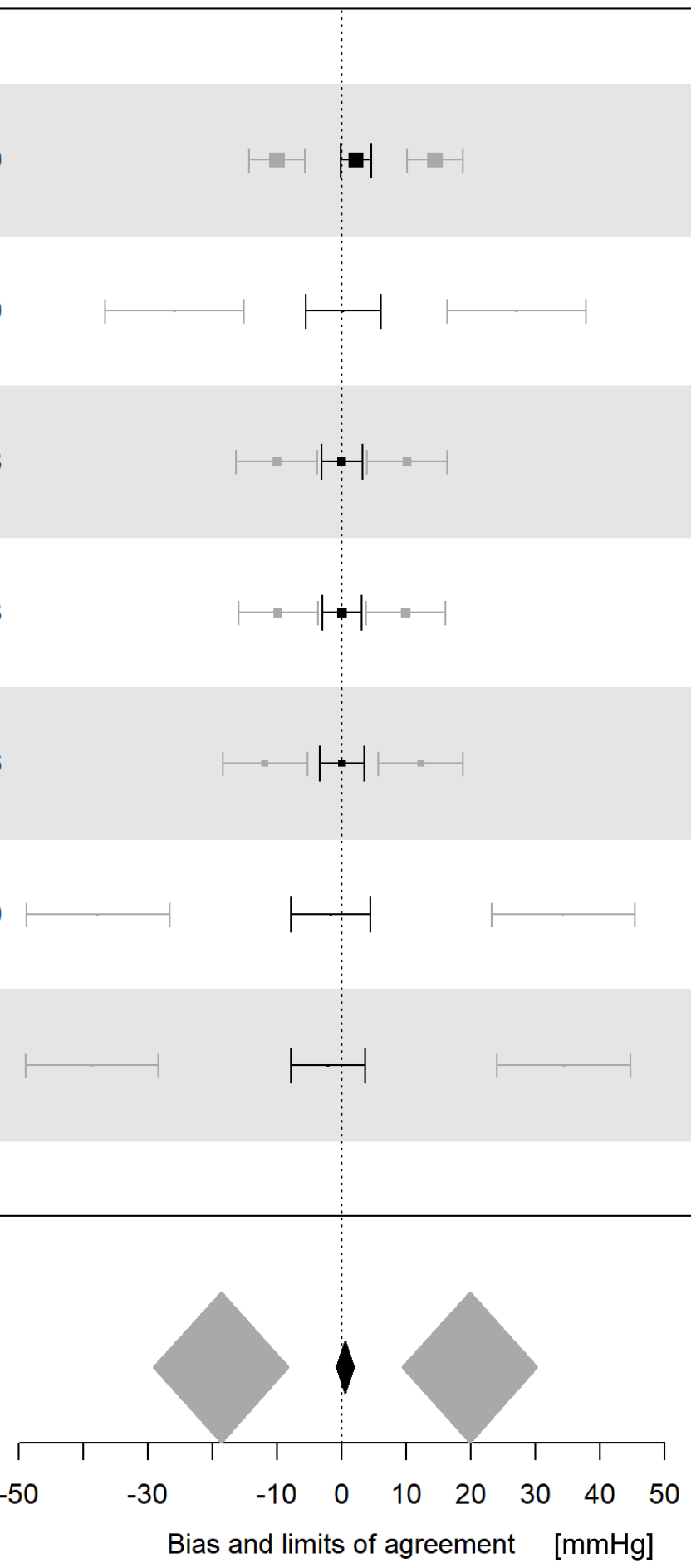

n number; *SBP* systolic blood pressure; *CI* confidence interval

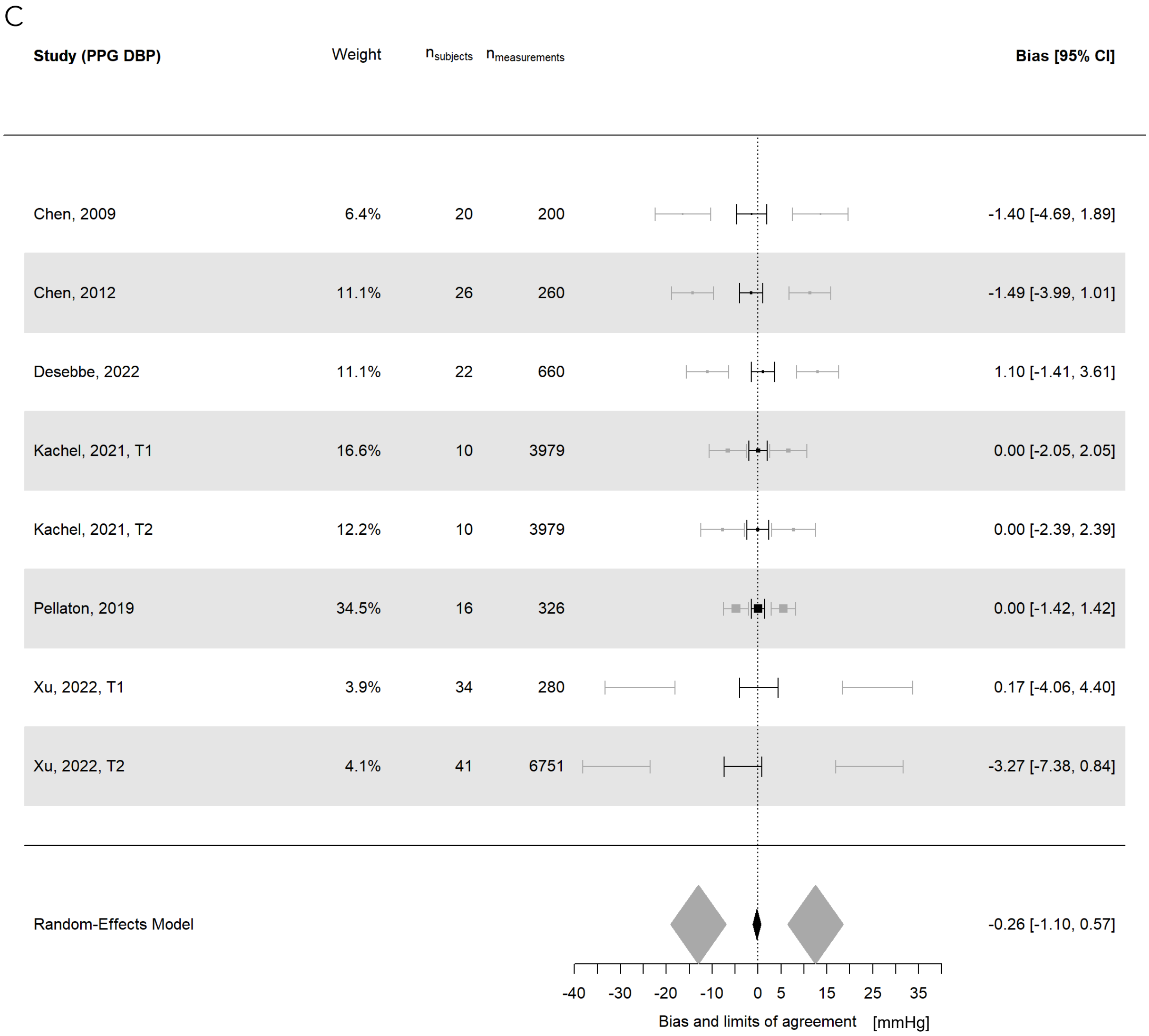

n number; *DBP* diastolic blood pressure; *CI* confidence interval
