# Supplementary material for: Accuracy and precision of continuous, non‐invasive blood pressure devices: a systematic review and meta‐analyses
Source: Anaesthesia. 2026 Jun 15;81(11):1581–92. doi: 10.1111/anae.70250 (PMC13659784; doi:10.1111/anae.70250)
Supplement: Supplementary file 2 [file ANAE-81-1581-s003.pdf]

Table S1: Overview of all included studies - Volume Clamp (Nexfin®)

literature	reference	n (measurements)	n (patients)	bias	SD	LLOA	ULOA	PE	setting	clinical data and patient characteristics	brand	manufacturer
	**Volume clamp (Nexfin®)**											
Balzer, 2016	MAP	100	20	-1	13	-25,5	24	27	orthopaedic surgery	elective orthopaedic surgery (total hip or knee replacement under general anaesthesia); age 68±10 years	Nexfin® monitor	Edwards LifeSciences Corporation, Irvine, CA, USA
Broch, 2013, T1	MAP	278	50	10,9	13	-14,6	36,5	34	cardiac surgery	elective heart surgery; mean age 69 ± 10 (30-87) years; 31m, 19f; before CPB	Nexfin™	BMEYE B.V., Amsterdam, Netherlands
Broch, 2013, T2	MAP	236	50	13,5	11,3	-8,6	35,7	29	cardiac surgery	elective heart surgery; mean age 69 ± 10 (30-87) years; 31m, 19f; after CPB	Nexfin™	BMEYE B.V., Amsterdam, Netherlands
Fischer, 2012	MAP	220	44	-4,6	6,5	-17,3	8,1	16,6	ICU after cardiac sugery	intensive care unit after conventional cardiac surgery; mean age 68 (22-85) years; 29m, 15f	Nexfin™	BMEYE B.V., Amsterdam, The Netherlands
Heusdens, 2016	MAP	3782	24	3,5	5,2	-6,9	13,9	10	vascular surgery	carotid endarterectomy under general anaesthesia, mean age 71 (51-84) years; 18m, 7f	Nexfin™	Edwards Lifesciences, Irvine, CA, USA
Hofhuizen, 2014	MAP	54	20	4,2	13,7	-9,5	18	18	ICU after cardiac sugery	sedated and ventilated post-cardiac surgery patients, mean age 67 (50-81) years, 17 male, 2 female*	Nexfin™	Edwards Lifesciences, Irvine, CA
Martina, 2012	MAP	9000	50	2,2	6,4	-10,6	15	17,9	cardiothoracic surgery	cardiothoracic surgery patients; mean age 63 ± 8 (34-83) years; 40m, 10f	Nexfin™	BMEYE B.V., Amsterdam, The Netherlands
Noto, 2019	MAP	2672	30	-6,8	6,7	-20,1	6,3	17	vascular surgery	regional anaesthesia during endarterectomy of the carotids in awake patients; mean age 67 (63-72) years; 21m, 9 f	Clearsight™	Edwards Lifesciences, Irvine, California, USA
Rogge, 2019	MAP	95963	35	1,1	7,4	-13,5	15,5	18,3	bariatric surgery	laparoscopic bariatric surgery; mean age 53 (41-59) years; 25m, 10f	Clearsight™ system	Edwards Lifesciences Corp, Irvine, CA
Sakai, 2018	MAP	210	10	-9,26	11,61	-32,02	13,5		urologic sugery (robotic)	robotic-assisted laparoscopic prostatectomy; mean age 65 (53-73) years	Clearsight™ system	BMEYE, Amsterdam, The Netherlands
Schramm, 2017	MAP	280	35	22,67	16,22	-9,12	54,46	35,1	neurosurgery	neurosurgical procedures in sitting position; mean age: 49 ± 14 years; 15m, 20f	Nexfin™ HD	BMeye (Amsterdam, The Netherlands)
Vos, 2014	MAP	758	110	2	9	-15	19	22	general surgery	elective general surgery under general anaesthesia, mean age 59 (22-84) years	Nexfin™	Edwards Lifesciences, Irvine, CA, USA
Wilde, 2016	MAP	57	19	0,82	5,03	-9,29	10,93		abdominal surgery	upper abdominal surgery; mean age 60.4 (± 6.9) years; 16m, 3f	Nexfin™	BMEYE Amsterdam, the Netherlands
Yokose, 2016	MAP	6312	30	7,9	7,73	-7,6	23,3	15,8	abdominal surgery	abdominal surgery (elective major hepatectomy, pancreatoduodenectomy, or both); mean age 71 (76-76) years; 18m, 12f	ClearSight™	Edwards Lifesciences, Irvine, CA, USA
Chachula, 2020, T1	MAP	63	10	-1,6	16	-32,9	29,7		orthopaedic surgery	Shoulder surgery in the **beach chair position;** mean age 68.5 (54.3–81.3) years; 5f, 5m	ClearSight	Edwards Lifesciences, Irvine, CA, USA
Chachula, 2020, T2	MAP	41	10	-2,8	15,3	-32,8	27,1		orthopaedic surgery	Shoulder surgery in the **supine position;** mean age 68.5 (54.3–81.3) years; 5f, 5m	ClearSight	Edwards Lifesciences, Irvine, CA, USA
Chachula, 2020, T3	MAP	104	10	-2,6	15,7	-33,4	28,2		orthopaedic surgery	Shoulder surgery **combined (supine and beach chair position) ;** mean age 68.5 (54.3–81.3) years; 5f, 5m	ClearSight	Edwards Lifesciences, Irvine, CA, USA

Eley, 2021	MAP	2229	30	5,2	10,9	-16	26,5		bariatric surgery	Participants with a body mass index (BMI) ≥45 kg/m2, laparoscopic bariatric surgery; mean age 45 ± 11.7 years; 26f, 4m	ClearSight™ EV1000 Clinical Platform	Edwards Lifesciences Corp, Irvine, CA
Fassaert, 2021	MAP	36206	48	4	7,8	-11,25	19,25		vascular surgery	after carotid endarteretomy; mean age 71,5 years (50-93); 12 f, 36 m	ClearSight system	Edwards Lifesciences, Irvine, CA
Frank, 2020	MAP	1404	52	-5,47	11,08	-27,2	16,26	25,95	cardiothoracic surgery	inducation of anesthesia undergoing major cardiac surgery; mean age 71 (35-84) years; 15 f, 37 m	ClearSight™ system, ClearSight™ EV1000 system	Edwards Lifesciences, Irvine, CA
Hansen, 2022	MAP	801	56	1,43	10	-20	20	22	bariatric surgery	patients undergoing laparoscopic bariatric surgery; mean age 46.5 (12.1) years; 44f, 16m	Nexfin® system	BMEYE, Amsterdam, The Netherlands, now licensed as Clearsight® system by Edwards Lifesciences, Irvine, CA, USA
Lee, 2021	MAP	8408	26	1,05	9,95	-18,85	20,95	25,23	thoracic surgery	one-lung ventilation; mean age 61.9±13.9 years; 5f, 21m	ClearSight	Edwards Lifesciences, Irvine, CA, USA
Lu, 2021	MAP	2243	20	0,3	7,4	-14	14		transcatheter aortic valve repacement	elective transcatheter aortic valve replacement; mean age 81±9 years; 7f, 13m	ClearSight™device, EV1000 Platform	Edwards Lifesciences Corp, Irvine, CA
Misugi, 2022	MAP	1277	21	-2,7	8	-18,3	12,8		caesarean delivery	woman at high risks of post-partum hemorrhage during cesarean section; mean age 34 (20-42) years;	ClearSight system	Edwards Lifesciences, Irvine, CA
Omenås, 2020	MAP	209	23	-4,3	5,83	-15,9	7,4		caesarean delivery	caesarean delivery; mean age 35.10 (3.2) years	ccNexfin monitor, version 1.9.0.1001	NexFin Systems, BMEYE, Amsterdan, NL
Schumann, 2021	MAP	538	90	-1	11	-23	21		bariatric surgery	bariatric surgery; mean age 47 ± 13 years; 64f, 26m	ccNexfin	BMEYE B.V., The Netherlands—now ClearSight (Edwards Lifesciences, USA)
Tanioku, 2020	MAP	3068	18	-3,9	7,8	-19,2	11,4		cardiovascular surgery	inducation of anesthesia for cardiovascular surgery; mean age 70 (64–79) years; 7f, 11m	ClearSight	Edwards Lifesciences, Irvine, CA, USA
Bugarini, 2021	MAP	1903	24	0,81	17,86	-52,52	54,14		neuroendovascular interventions	perioperative monitoring during neuroendovascular interventions; mean age 58,8 (13-79) years; 14f, 10m	ClearSight system	Edwards Lifesciences, Irvine, CA
Berkelmans, 2018 T1	MAP	4650	31	0	8	-15	15	18	intensive or medium care	patients with atrial fibrillation admitted to the intensive or medium care unit; mean age 74 (9) years, 11f, 19 m	Nexfin	BMEYE, Amsterdam, The Netherlands
Berkelmans, 2018 T2	MAP	1500	10	1	5	-10	11	13	intensive or medium care	patients with sinus rhythm admitted to the intensive or medium care unit; mean age 64 (17) years; 4f, 6m	Nexfin	BMEYE, Amsterdam, The Netherlands
Kho, 2022 T1	MAP	22308	33	-4,5	4,6	-13,4	4,4	6,9	non-cardiac surgery	Adult patients scheduled for non-cardiac surgery, **brachial artery BP (BPBra) (the original algorithm from ClearSight);** mean age 65 (11) years; 17f, 16m	ClearSight	Edwards Lifesciences, Irvine, CA, USA
Kho, 2022 T2	MAP	22308	33	1,1	6	-10,6	12,8	9	non-cardiac surgery	Adult patients scheduled for non-cardiac surgery, **radial artery BP (BPRad) (the novel algorithm);** mean age 65 (11) years; 17f, 16m	ClearSight	Edwards Lifesciences, Irvine, CA, USA
		**229182**	**1072**									

	Volume clamp (Nexfin®)											
Balzer, 2016	SBP	100	20	5	16	-26,5	36,9	25	orthopaedic surgery	elective orthopaedic surgery (total hip or knee replacement under general anaesthesia); age 68±10 years	Nexfin® monitor	Edwards LifeSciences Corporation, Irvine, CA, USA
Broch, 2013, T1	SBP	278	50	11,4	17,1	-22	44,9	29	cardiac surgery	elective heart surgery; mean age 69 ± 10 (30-87) years; 31m, 19f; before CPB	Nexfin™	BMEYE B.V., Amsterdam, Netherlands
Broch, 2013, T2	SBP	236	50	15,1	17,9	-20,2	50,3	30	cardiac surgery	elective heart surgery; mean age 69 ± 10 (30-87) years; 31m, 19f; after CPB	Nexfin™	BMEYE B.V., Amsterdam, Netherlands
Fischer, 2012	SBP	220	44	5,7	14,13	-22,5	34	25,6	ICU after cardiac sugery	intensive care unit after conventional cardiac surgery; mean age 68 (22-85) years; 29m, 15f	Nexfin™	BMEYE B.V., Amsterdam, The Netherlands
Heusdens, 2016	SBP	3782	24	-3,3	10,8	-24,9	18,3	13,5	vascular surgery	carotid endarterectomy under general anaesthesia, mean age 71 (51-84) years; 18m, 7f	Nexfin™	Edwards Lifesciences, Irvine, CA, USA
Hofhuizen, 2014	SBP	54	20	2,7	11,1	-19,5	24,9	20	ICU after cardiac sugery	sedated and ventilated post-cardiac surgery patients	Nexfin™	Edwards Lifesciences, Irvine, CA
Martina, 2012	SBP	9000	50	-0,5	6,7	-13,9	12,9	13,5	cardiothoracic surgery	cardiothoracic surgery patients; mean age 63 ± 8 (34-83) years; 40m, 10f	Nexfin™	BMEYE B.V., Amsterdam, The
Noto, 2019	SBP	2672	30	-3	9,7	-22,1	16	16,2	vascular surgery	regional anaesthesia during endarterectomy of the carotids in awake patients; mean age 67 (63-72) years; 21m, 9 f	Clearsight™	Edwards Lifesciences, Irvine, California, USA
Rogge, 2019	SBP	95963	35	6,8	10,3	-14,4	27,9	18,3	bariatric surgery	laparoscopic bariatric surgery; mean age 53 (41-59) years; 25m, 10f	Clearsight™ system	Edwards Lifesciences Corp, Irvine, CA
Sakai, 2018	SBP	210	10	-2,99	15,8	-34,04	28,06		urologic sugery (robotic)	robotic-assisted laparoscopic prostatectomy; mean age 65 (53-73) years	Clearsight™ system	BMEYE, Amsterdam, The Netherlands
Schramm, 2017	SBP	280	35	14,14	19,08	-23,26	51,55	28,8	neurosurgery	neurosurgical procedures in sitting position; mean age: 49 ± 14 years; 15m, 20f	Nexfin™ HD	BMeye (Amsterdam, The Netherlands)
Weiss, 2014	SBP	3479	31	-3,8	16,13	-36	28,5	34	general surgery	elective surgery under general anaesthesia; meag age 63 (24-89) years; 14m, 17f	Nexfin™	BMEYE B.V., Amsterdam, Netherlands
Wilde, 2016	SBP	57	19	-3,3	7,83	-19,04	12,44		abdominal surgery	upper abdominal surgery ; mean age 60.4 (± 6.9) years; 16m, 3f	Nexfin™	BMEYE Amsterdam, the Netherlands
Yokose, 2016	SBP	6312	30	0,7	12,65	-24,6	26	17,9	abdominal surgery	abdominal surgery (elective major hepatectomy, pancreatoduodenectomy, or both); mean age 71 (76-76) years; 18m, 12f	ClearSight™	Edwards Lifesciences, Irvine, CA, USA
Eley, 2021	SBP	2229	30	14,3	14,1	-13,4	42		bariatric surgery	Participants with a body mass index (BMI) ≥45 kg/m2, laparoscopic bariatric surgery; mean age 45 ± 11.7 years; 26f, 4m	ClearSight™ EV1000 Clinical Platform	Edwards Lifesciences Corp, Irvine, CA
Fassaert, 2021	SBP	36206	48	-9,58	13,6	-36,31	17,15		vascular surgery	after carotid endarteretomy; mean age 71,5 years (50-93); 12 f, 36 m	ClearSight system	Edwards Lifesciences, Irvine, CA
Frank, 2020	SBP	1404	52	-18,05	16,78	-50,94	14,84	26,77	cardiothoracic surgery	inducation of anesthesia undergoing major cardiac surgery; mean age 71 (35-84) years; 15 f, 37 m	ClearSight™ system, ClearSight™ EV1000 system monitor	Edwards Lifesciences, Irvine, CA
Lee, 2021	SBP	8408	26	-5,18	16,32	-37,81	27,45	31,01	thoracic surgery	one-lung ventilation; mean age 61.9±13.9 years; 5f, 21m	ClearSight	Edwards Lifesciences, Irvine, CA, USA

Lu, 2021	SBP	2243	20	9,8	10,1	-10	30		transcatheter aortic valve repacement	elective transcatheter aortic valve replacement; mean age 81±9 years; 7f, 13m	ClearSight™device, EV1000 Platform	Edwards Lifesciences Corp, Irvine, CA
Misugi, 2022	SBP	1277	21	0,8	13,4	-17,1	35,1		caesarean delivery	woman at high risks of post-partum hemorrhage during cesarean section; mean age 34 (20-42) years;	ClearSight system	Edwards Lifesciences, Irvine, CA
Omenås, 2020	SBP	209	23	12,2	11,68	-11,1	35,6		caesarean delivery	caesarean delivery; mean age 35.10 (3.2) years	ccNexfin monitor, version 1.9.0.1001	NexFin Systems, BMEYE, Amsterdan, NL
Schumann, 2021	SBP	538	90	-7	14	-35	20		bariatric surgery	bariatric surgery; mean age 47 ± 13 years; 64f, 26m	ccNexfin	BMEYE B.V., The Netherlands—now ClearSight (Edwards Lifesciences, USA)
Tanioku, 2020	SBP	3068	18	13,2	17,5	-21,1	47,4		cardiothoracic surgery	inducation of anesthesia for cardiovascular surgery; mean age 70 (64–79) years; 7f, 11m	ClearSight	Edwards Lifesciences, Irvine, CA, USA
Bugarini, 2021	SBP	1903	24	5,38	14,64	-45,12	56,08		neuroendovascular interventions	perioperative monitoring during neuroendovascular interventions; mean age 58,8 (13-79) years; 14f, 10m	ClearSight system	Edwards Lifesciences, Irvine, CA
Berkelmans, 2018 T1	SBP	4650	31	-4	12	-28	20	21	intensive or medium care	patients with atrial fibrillation admitted to the intensive or medium care unit; mean age 74 (9) years, 11f, 19 m	Nexfin	BMEYE, Amsterdam, The Netherlands
Berkelmans, 2018 T2	SBP	1500	10	-6	10	-26	15	18	intensive or medium care	patients with sinus rhythm admitted to the intensive or medium care unit; mean age 64 (17) years; 4f, 6m	Nexfin	BMEYE, Amsterdam, The Netherlands
Kho, 2022 T1	SBP	22308	33	2,7	9,3	-15,6	20,9	8,8	non-cardiac surgery	Adult patients scheduled for non-cardiac surgery, **brachial artery BP (BPBra) (the original algorithm from ClearSight);** mean age 65 (11) years; 17f, 16m	ClearSight	Edwards Lifesciences, Irvine, CA, USA
Kho, 2022 T2	SBP	22308	33	1,9	10,6	-18,9	22,6	10,4	non-cardiac surgery	Adult patients scheduled for non-cardiac surgery, **radial artery BP (BPRad) (the novel algorithm);** mean age 65 (11) years; 17f, 16m	ClearSight	Edwards Lifesciences, Irvine, CA, USA
		**230894**	**907**									
	**Volume clamp (Nexfin®)**											
Balzer, 2016	DBP	100	20	-5	12	-29,6	19,5	33	orthopaedic surgery	elective orthopaedic surgery (total hip or knee replacement under general anaesthesia); age 68±10 years	Nexfin® monitor	Edwards LifeSciences Corporation, Irvine, CA, USA
Broch, 2013, T1	DBP	278	50	13	13,5	-14,6	39,5	47	cardiac surgery	elective heart surgery; mean age 69 ± 10 (30-87) years; 31m, 19f; before CPB	Nexfin™	BMEYE B.V., Amsterdam,
Broch, 2013, T2	DBP	236	50	13,7	12,1	-10	37,4	41	cardiac surgery	elective heart surgery; mean age 69 ± 10 (30-87) years; 31m, 19f; after CPB	Nexfin™	BMEYE B.V., Amsterdam, Netherlands
Fischer, 2012	DBP	220	44	-8,9	6,83	-22,6	4,7	22,01	ICU after cardiac sugery	intensive care unit after conventional cardiac surgery; mean age 68 (22-85) years; 29m, 15f	Nexfin™	BMEYE B.V., Amsterdam, The Netherlands
Heusdens, 2016	DBP	3782	24	6,1	5,7	-5,3	17,5	15,3	vascular surgery	carotid endarterectomy under general anaesthesia, mean age 71 (51-84) years; 18m, 7f	Nexfin™	Edwards Lifesciences, Irvine, CA, USA
Hofhuizen, 2014	DBP	54	20	4,9	6,8	-8,7	18,5	22,8	ICU after cardiac sugery	sedated and ventilated post-cardiac surgery patients	Nexfin™	Edwards Lifesciences, Irvine, CA
Martina, 2012	DBP	9000	50	2,8	6,4	-10	15,6	22,3	cardiothoracic surgery	cardiothoracic surgery patients; mean age 63 ± 8 (34-83) years; 40m, 10f	Nexfin™	BMEYE B.V., Amsterdam, The Netherlands

Noto, 2019	DBP	2672	30	-9	5,4	-19,7	1,5	18,9	vascular surgery	regional anaesthesia during endarterectomy of the carotids in awake patients; mean age 67 (63-72) years; 21m, 9 f	Clearsight™	Edwards Lifesciences, Irvine, California, USA
Rogge, 2019	DBP	95963	35	0,8	6,9	-12,9	14,4	21,5	bariatric surgery	laparoscopic bariatric surgery; mean age 53 (41-59) years; 25m, 10f	Clearsight™ System	Edwards Lifesciences Corp, Irvine, CA
Sakai, 2018	DBP	210	10	-12,03	10,84	-33,27	9,21		urologic sugery (robotic)	robotic-assisted laparoscopic prostatectomy; mean age 65 (53-73) years	Clearsight™ System	BMEYE, Amsterdam, The Netherlands
Schramm, 2017	DBP	280	35	24,69	15,03	-4,76	54,15	44	neurosurgery	neurosurgical procedures in sitting position; mean age: 49 ± 14 years; 15m, 20f	Nexfin™ HD	BMeye (Amsterdam, The Netherlands)
Weiss, 2014	DBP	3479	31	-8,8	10,53	-29,8	12,3	37	general surgery	elective surgery under general anaesthesia; meag age 63 (24-89) years; 14m, 17f	Nexfin™	BMEYE B.V., Amsterdam, Netherlands
Wilde, 2016	DBP	57	19	2,42	4,2	-6,02	10,86		abdominal surgery	upper abdominal surgery; mean age 60.4 (± 6.9) years; 16m, 3f	Nexfin™	BMEYE Amsterdam, the Netherlands
Yokose, 2016	DBP	6312	30	10,1	6,95	-3,8	24	19,3	abdominal surgery	abdominal surgery (elective major hepatectomy, pancreatoduodenectomy, or both); mean age 71 (76-76) years; 18m, 12f	ClearSight™	Edwards Lifesciences, Irvine, CA, USA
Eley, 2021	DBP	2229	30	2,6	10,8	-18,6	23,8		bariatric surgery	Participants with a body mass index (BMI) ≥45 kg/m2, laparoscopic bariatric surgery; mean age 45 ± 11.7 years; 26f, 4m	ClearSight™ EV1000 Clinical Platform	Edwards Lifesciences Corp, Irvine, CA
Fassaert, 2021	DBP	36206	48	7,92	7,2	-6,17	22,01		vascular surgery	after carotid endarteretomy; mean age 71,5 years (50-93); 12 f, 36 m	ClearSight system	Edwards Lifesciences, Irvine, CA
Frank, 2020	DBP	1404	52	-1,09	11,15	-22,94	20,76	34,16	cardiothoracic surgery	inducation of anesthesia undergoing major cardiac surgery; mean age 71 (35-84) years; 15 f, 37 m	ClearSight™ system, ClearSight™ EV1000 system monitor	Edwards Lifesciences, Irvine, CA
Lee, 2021	DBP	8408	26	5,16	8,78	-12,41	22,72	27,12	thoracic surgery	one-lung ventilation; mean age 61.9±13.9 years; 5f, 21m	ClearSight	Edwards Lifesciences, Irvine, CA, USA
Lu, 2021	DBP	2243	20	-5,9	7,8	-21	9		transcatheter aortic valve repacement	elective transcatheter aortic valve replacement; mean age 81±9 years; 7f, 13m	ClearSight™device, EV1000 Platform	Edwards Lifesciences Corp, Irvine, CA
Misugi, 2022	DBP	1277	21	-6,3	7,1	-20,2	7,6		caesarean delivery	woman at high risks of post-partum hemorrhage during cesarean section; mean age 34 (20-42) years;	ClearSight system	Edwards Lifesciences, Irvine, CA
Schumann, 2021	DBP	538	90	0	11	-22	22		bariatric surgery	bariatric surgery; mean age 47 ± 13 years; 64f, 26m	ccNexfin	BMEYE B.V., The Netherlands—now ClearSight (Edwards Lifesciences, USA)
Tanioku, 2020	DBP	3068	18	-9,1	7,3	-23,4	5,2		cardiothoracic surgery	inducation of anesthesia for cardiovascular surgery; mean age 70 (64–79) years; 7f, 11m	ClearSight	Edwards Lifesciences, Irvine, CA, USA
Bugarini, 2021	DBP	1903	24	5,38	14,64	-45,12	56,08		neuroendovascular interventions	perioperative monitoring during neuroendovascular interventions; mean age 58,8 (13-79) years; 14f, 10m	ClearSight system	Edwards Lifesciences, Irvine, CA
Berkelmans, 2018 T1	DBP	4650	31	1	7	-13	15	22	intensive or medium care unit	patients with atrial fibrillation admitted to the intensive or medium care unit; mean age 74 (9) years, 11f, 19 m	Nexfin	BMEYE, Amsterdam, The Netherlands
Berkelmans, 2018 T2	DBP	1500	10	4	6	-7	16	19	intensive or medium care	patients with sinus rhythm admitted to the intensive or medium care unit; mean age 64 (17) years; 4f, 6m	Nexfin	BMEYE, Amsterdam, The Netherlands

Kho, 2022 T1	DBP	22308	33	-6,4	4,3	-14,8	2	8,2	non-cardiac surgery	Adult patients scheduled for non-cardiac surgery, **brachial artery BP (BPBra) (the original algorithm from ClearSight);** mean age 65 (11) years; 17f, 16m	ClearSight	Edwards Lifesciences, Irvine, CA, USA
Kho, 2022 T2	DBP	22308	33	0,7	6	-11	12,3	11,5	non-cardiac surgery	Adult patients scheduled for non-cardiac surgery, **radial artery BP (BPRad) (the novel algorithm);** mean age 65 (11) years; 17f, 16m	ClearSight	Edwards Lifesciences, Irvine, CA, USA
		**230685**	**884**									

Table S2: Overview of all included studies - Volume Clamp (CNAP®)

literature	reference	n (measurements)	n (patients)	bias	SD	LLOA	ULOA	PE	setting	clinical data and patient characteristics	brand	manufacturer
	**Volume clamp (CNAP®)**											
Araz, 2015	MAP	5433	40	0,4	*10,40*	-20,4	21,2		abdominal surgery	hepatic lobectomy for liver transplant in living-liver donors; mean age 34.9 ± 7.9 (24-53) years; 19m, 21f	Infinity CNAP™	Draeger Medical Systems, Drager Medical GmbH, Lübeck, Germany
Biais, 2019	MAP	1452	25	-1,8	10,3	-22	18,3	91	vascular surgery	major vascular surgery; age 69 ± 11 years, 14m, 11f	Infinity CNAP™ SmartPod	Dräger Medical AG & Co. KG, Lübeck, Germany
Gayat, 2013	MAP	5174	52	-8	*13,25*	-35	18	32	general surgery	surgery under general anaesthesia; mean age 67 (59-76) years; 33m	CNAP®	CNSystems Medizintechnik AG, Graz, Austria
Hahn, 2012, T1	MAP	249508	50	-2,9	10,6	-23,7	17,9	*28,3*	general surgery	elective surgery under general anaesthesia; mean age 65,1 (33-87); 27m, 23f; Software V3.0	CNAP™ Monitor 500	CNSystems Medizintechnik AG, Graz, Austria
Hahn, 2012, T2	MAP	237562	50	-3,1	9,45	-21,6	15,4	*25,4*	general surgery	elective surgery under general anaesthesia; mean age 62.9 (30-96); 30m, 20f; Software V3.5	CNAP™ Monitor 500	CNSystems Medizintechnik AG, Graz, Austria
Huhle, 2016	MAP	100	10	-2,8	8,9	-21	21		neurosurgery	neurosurgical intervention during general and total invasive anaesthesia (TIVA); mean age 63±13 years; 6m, 4f	CNAP™ Monitor 500	CNSystems Medizintechnik AG, Graz, Austria
Ilies, 2012, T1	MAP	4093	85	-10,21	11,3	-35,87	15,45	24,7	general surgery	surgery under general anaesthesia; mean age 65 (31-85) years; 52m, 33f; induction of anaesthesia	CNAP™ Monitor 500	CNSystems Medizintechnik AG, Graz, Austria
Ilies, 2012, T2	MAP	9353	85	-4,34	6,8	-23,83	15,16	15,8	general surgery	surgery under general anaesthesia; mean age 65 (31-85) years; 52m, 33f; maintenance of anaesthesia	CNAP™ Monitor 500	CNSystems Medizintechnik AG, Graz, Austria
Ilies, 2015	MAP	11222	104	-6,1	7,6	-22,4	10,1	18,4	cardiosurgical ICU	cardiosurgical ICU with ventilated patients; mean age 68,8 years; 20m, 84f	CNAP™ Monitor 500 (version 3.5)	CNSystems Medizintechnik AG, Graz, Austria
Jeleazcov, 2010	MAP	156000	78	-1,6	11	-13,6	10,4		abdominal surgery	elective abdominal surgery, cardio- oder neurosurgery; mean age 60 (27-85) years; 44m, 34f	CNAP®	CNSystems Medizintechnik AG, Graz, Austria
Kumar, 2015	MAP	1200	60	0,02	5,68	-11,17	11,12		cardiac surgery	induction of general anaesthesia in cardiac surgery; 36m, 24f	Infinity® (CNAP™) SmartPod®	Draeger Medical Systems, Drager Medical GmbH, Lübeck, Germany
Rogge, 2018	MAP	6864	29	7,9	9,6	-11,2	27		bariatric surgery	laprascopic bariatric surgery; mean age 43 (24-67) years; 9m, 22f	CNAP™ system	CNSystems Medizintechnik AG, Graz, Austria
Sackl-Pietsch, 2008	MAP	75485	15	6,65	11,23	-15,78	29,09		cardiac/vascular/orthopaedic surgery	cardiac, vascular or orthopaedic surgery; mean age 71 (33-82) years; 8m, 7f	CNAP™ Monitor 500i	CNSystems Medizintechnik AG, Graz, Austria

Schramm, 2010	MAP	48691	29	-0,8	15	-31	29	37,3	cardiac surgery	elective transfemoral aortic valve implantation; mean age 82 ± 5 years; 12m, 17f	CNAP, software version 9.94	CNSystems Medizintechnik AG, Graz, Austria
Smolle, 2015	MAP	7200	40	4,6	6,7	-8,7	17,8	6,8	ICU	ICU: sedated patients on vasopressor therapy; mean age 66 (56-72) years; 27m, 13f	CNAP®	CNSystems Medizintechnik AG, Graz, Austria
Wang, 2019	MAP	7990	48	4,5	8,5	-12,5	21,5		general surgery	surgery under general anaesthesia; mean age 48 (19-68) years; 26m, 22f	CNAP™ Monitor 500	CNSystems Medizintechnik AG, Graz, Austria
Flick, 2021	MAP	276907	49	9	11,2	-12,9	30,9		neurosugery	neurosurgery; mean age 55 (19 to 80) years;	CNAP™ Monitor 501	CNSystems Medizintechnik AG, Graz, Austria
		**1104234**	**849**									
	**Volume clamp (CNAP®)**											
Araz, 2015	SBP	5433	40	-0,3	*16,00*	-32,3	31,7		abdominal surgery	hepatic lobectomy for liver transplant in living-liver donors; mean age 34.9 ± 7.9 (24-53) years; 19m, 21f	Infinity CNAP™	Draeger Medical Systems, Drager Medical GmbH, Lübeck, Germany
Biais, 2019	SBP	1452	25	7,2	12,7	-17,7	32,2	69	vascular surgery	major vascular surgery; age 69 ± 11 years, 14m, 11f	Infinity CNAP™ SmartPod	Dräger Medical AG & Co. KG, Lübeck, Germany
Gayat, 2013	SBP	5174	52	-2	*11,50*	-45	41	37	general surgery	surgery under general anaesthesia; mean age 67 (59-76) years; 33m	CNAP®	CNSystems Medizintechnik AG, Graz, Austria
Hahn, 2012, T1	SBP	249508	50	3,4	16	-27,9	34,8	*30,8*	general surgery	elective surgery under general anaesthesia; mean age 65,1 (33-87); 27m, 23f; Software V3.0	CNAP™ Monitor 500	CNSystems Medizintechnik AG, Graz, Austria
Hahn, 2012, T2	SBP	237562	50	0,9	13,2	-24,9	26,8	*25,6*	general surgery	elective surgery under general anaesthesia; mean age 62.9 (30-96); 30m, 20f; Software V3.5	CNAP™ Monitor 500	CNSystems Medizintechnik AG, Graz, Austria
Ilies, 2012, T1	SBP	4093	85	-3,31	16,5	-43,01	36,39	25,5	general surgery	surgery under general anaesthesia; mean age 65 (31-85) years; 52m, 33f; induction of anaesthesia	CNAP™ Monitor 500	CNSystems Medizintechnik AG, Graz, Austria
Ilies, 2012, T2	SBP	9353	85	4,17	10	-26,63	34,98	16,6	general surgery	surgery under general anaesthesia; mean age 65 (31-85) years; 52m, 33f; maintenance of anaesthesia	CNAP™ Monitor 500	CNSystems Medizintechnik AG, Graz, Austria
Ilies, 2015	SBP	11222	104	4,3	11,6	-21,7	30,4	22,8	cardiosurgical ICU	cardiosurgical ICU with ventilated patients; mean age 68,8 years; 20m, 84f	CNAP™ Monitor 500 (version 3.5)	CNSystems Medizintechnik AG, Graz, Austria
Jeleazcov, 2010	SBP	156000	78	6,7	13,9	-10,9	24,3		abdominal surgery	elective abdominal surgery, cardio- oder neurosurgery; mean age 60 (27-85) years; 44m, 34f	CNAP®	CNSystems Medizintechnik AG, Graz, Austria

Kumar, 2015	SBP	1200	60	5,98	10,36	-14,33	26,29		cardiac surgery	induction of general anaesthesia in cardiac surgery; 36m, 24f	Infinity® (CNAP™) SmartPod®	Draeger Medical Systems, Drager Medical GmbH, Lübeck, Germany
Rogge, 2018	SBP	6864	29	4,8	15,8	-26,5	36		bariatric surgery	laprascopic bariatric surgery; mean age 43 (24-67) years; 9m, 22f	CNAP™ system	CNSystems Medizintechnik AG, Graz, Austria
Sackl-Pietsch, 2008	SBP	75485	15	-2,96	13,81	*-30,58*	*24,66*		cardiac/vascular/ orthopaedic surgery	cardiac, vascular or orthopaedic surgery; mean age 71 (33-82) years; 8m, 7f	CNAP™ Monitor 500i	CNSystems Medizintechnik AG, Graz, Austria
Schramm, 2010	SBP	47664	29	-11	18	-47	25	*29,5*	cardiac surgery	elective transfemoral aortic valve implantation; mean age 82 ± 5 years; 12m, 17f	CNAP, software version 9.94	CNSystems Medizintechnik AG, Graz, Austria
Smolle, 2015	SBP	7200	40	-3,2	10,1	-23,1	16,6	9,12	ICU	ICU: sedated patients on vasopressor therapy; mean age 66 (56-72) years; 27m, 13f	CNAP®	CNSystems Medizintechnik AG, Graz, Austria
Wang, 2019	SBP	7990	48	-6,3	15,9	-38,1	25,4		general surgery	surgery under general anaesthesia; mean age 48 (19-68) years; 26m, 22f	CNAP™ Monitor 500	CNSystems Medizintechnik AG, Graz, Austria
Flick, 2021	SBP	276907	49	2,5	15	-26,8	31,9		neurosugery	neurosurgery; mean age 55 (19 to 80) years;	CNAP™ Monitor 501	CNSystems Medizintechnik AG, Graz, Austria
		**1103107**	**839**									
	**Volume clamp (CNAP®)**											
Araz, 2015	DBP	5433	40	-0,4	*10,60*	-21,6	20,8		abdominal surgery	hepatic lobectomy for liver transplant in living-liver donors; mean age 34.9 ± 7.9 (24-53) years; 19m, 21f	Infinity CNAP™	Draeger Medical Systems, Drager Medical GmbH, Lübeck, Germany
Biais, 2019	DBP	1452	25	-7,5	10,1	-27,3	12,4	86	vascular surgery	major vascular surgery; age 69 ± 11 years, 14m, 11f	Infinity CNAP™ SmartPod	Dräger Medical AG & Co. KG, Lübeck, Germany
Gayat, 2013	DBP	5174	52	-11	*11,50*	-34	12	37	general surgery	surgery under general anaesthesia; mean age 67 (59-76) years; 33m	CNAP®	CNSystems Medizintechnik AG, Graz, Austria
Hahn, 2012, T1	DBP	249508	50	-4,4	10,8	-25,5	16,8	*37,2*	general surgery	elective surgery under general anaesthesia; mean age 65,1 (33-87); 27m, 23f; Software V3.0	CNAP™ Monitor 500	CNSystems Medizintechnik AG, Graz, Austria
Hahn, 2012, T2	DBP	237562	50	-2,8	8,6	-19,7	14,1	*29,7*	general surgery	elective surgery under general anaesthesia; mean age 62.9 (30-96); 30m, 20f; Software V3.5	CNAP™ Monitor 500	CNSystems Medizintechnik AG, Graz, Austria
Ilies, 2012, T1	DBP	4093	85	-10,78	10,7	-35,44	13,88	24,8	general surgery	surgery under general anaesthesia; mean age 65 (31-85) years; 52m, 33f; induction of anaesthesia	CNAP™ Monitor 500	CNSystems Medizintechnik AG, Graz, Austria

Ilies, 2012, T2	DBP	9353	85	-5,81	6	-23,38	11,75	18	general surgery	surgery under general anaesthesia; mean age 65 (31-85) years; 52m, 33f; maintenance of anaesthesia	CNAP™ Monitor 500	CNSystems Medizintechnik AG, Graz, Austria
Ilies, 2015	DBP	11222	104	-9,4	8	-26,4	7,5	25,6	cardiosurgical ICU	cardiosurgical ICU with ventilated patients; mean age 68,8 years; 20m, 84f	CNAP™ Monitor 500 (version 3.5)	CNSystems Medizintechnik AG, Graz, Austria
Jeleazcov, 2010	DBP	156000	78	-5,6	11,4	-17	5,8		abdominal surgery	elective abdominal surgery, cardio- oder neurosurgery; mean age 60 (27-85) years; 44m, 34f	CNAP®	CNSystems Medizintechnik AG, Graz, Austria
Kumar, 2015	DBP	1200	60	-3,72	6,1	-5,68	8,23		cardiac surgery	induction of general anaesthesia in cardiac surgery; 36m, 24f	Infinity® (CNAP™) SmartPod®	Draeger Medical Systems, Drager Medical GmbH, Lübeck, Germany
Rogge, 2018	DBP	6864	29	9,5	10,3	-10,9	29,9		bariatric surgery	laprascopic bariatric surgery; mean age 43 (24-67) years; 9m, 22f	CNAP™ system	CNSystems Medizintechnik AG, Graz, Austria
Sackl-Pietsch, 2008	DBP	75485	15	12,36	10,91	*-9,46*	*34,18*		cardiac/vascular/ orthopaedic surgery	cardiac, vascular or orthopaedic surgery; mean age 71 (33-82) years; 8m, 7f	CNAP™ Monitor 500i	CNSystems Medizintechnik AG, Graz, Austria
Schramm, 2010	DBP	48642	29	6	16	-26	38	*55,2*	cardiac surgery	elective transfemoral aortic valve implantation; mean age 82 ± 5 years; 12m, 17f	CNAP, software version 9.94	CNSystems Medizintechnik AG, Graz, Austria
Smolle, 2015	DBP	7200	40	7	6,7	-6,1	20,2	9,7	ICU	ICU: sedated patients on vasopressor therapy; mean age 66 (56-72) years; 27m, 13f	CNAP®	CNSystems Medizintechnik AG, Graz, Austria
Wang, 2019	DBP	7990	48	9,9	7,5	-5,1	24,9		general surgery	surgery under general anaesthesia; mean age 48 (19-68) years; 26m, 22f	CNAP™ Monitor 500	CNSystems Medizintechnik AG, Graz, Austria
Flick, 2021	DBP	276907	49	10,1	10,8	-11	31,2		neurosugery	neurosurgery; mean age 55 (19 to 80) years;	CNAP™ Monitor 501	CNSystems Medizintechnik AG, Graz, Austria
		**1104085**	**839**									

Table S3: Overview of all included studies - Applanation tonometry

literature	reference	n (measurements)	n (patients)	bias	SD	LLOA	ULOA	PE	setting	clinical data and patient characteristics	brand	manufacturer
	**Applanation tonometry (T-Line®)**											
Dueck, 2012	SBP	4747	19	2,3	7,8	-13	17,6	*26*	general surgery	general anesthesia for a wide range of surgical procedures; mean age 60,7 years; 11m, 8f	Tensys TL-200™	Tensys Medical, San Diego, CA, USA
Greiwe, 2016	SBP	201907	28	3,45	*12,73*	*-22*	28,9	23,4	bariatric surgery	bariatric surgery; mean age 44,8 (20-68) years; 10m, 18f	T-Line 200pro™	Tensys Medical®, USA
Janelle, 2006	SBP	17009	25	1,7	7	*-12,3*	*15,7*	*12,8*	general surgery	surgical patients; mean age 61 ± 15 years; 15m, 10f	T-Line® Tensymeter, TL-100	Tensys Medical, Inc., San Diego, CA
Langwieser, 2015	SBP	7304	30	-6	11	-28	15	*20*	cardiosurgical ICU	cardiosurgical ICU; mean age 69 (60-77) years; 21m, 9f	T-Line 200pro™	Tensys Medical Inc., San Diego, CA, USA
Lin, 2017	SBP	4381	23	1,3	5,87	-10,2	12,8	*10,4*	neurosurgery	elective neurosurgery; mean age 53.9±7 years; 14m, 9f	TL-300™	Tensys Medical Inc., San Diego, CA
Meidert, 2014	SBP	2993	24	-3	15	-33	27	*23*	ICU	ICU; mean age 67 (54-77) years; 15m, 9f	TL-200™ or TL-200pro™	Tensys Medical Inc., San Diego, CA, USA
Sun, 2017	SBP	1538	30	-0,9	7,6	-15,7	13,9	*13,1*	abdominal surgery	colon carcinoma surgery; mean age 61,3 (26-83) years; 15m, 15f	TL-300™	Tensys Medical Inc., San Diego, CA, USA
Szmuk, 2008	SBP	5425	22	0	7,9	-17	15	*16,1*	spine surgery	multilevel spine surgery under general anaesthesia with induced hypotension; mean age 48,6 (18-70) years; 10m, 15f	TL100™	Tensys Medical Inc., San Diego, CA, USA
		**245304**	**201**									
	**Applanation tonometry (T-Line®)**											
Dueck, 2012	DBP	4747	19	1,7	6,2	-10,6	14	*20*	general surgery	general anesthesia for a wide range of surgical procedures; mean age 60,7 years; 11m, 8f	Tensys TL-200™	Tensys Medical, San Diego, CA, USA
Greiwe, 2016	DBP	201907	28	3,66	*9,69*	-15,7	23,07	*30,3*	bariatric surgery	bariatric surgery; mean age 44,8 (20-68) years; 10m, 18f	T-Line 200pro™	Tensys Medical®, USA
Janelle, 2006	DBP	17009	25	2,3	6,9	*-11,5*	*16,1*	*23,9*	general surgery	surgical patients; mean age 61 ± 15 years; 15m, 10f	T-Line® Tensymeter, TL-100	Tensys Medical, Inc., San Diego, CA
Langwieser, 2015	DBP	7304	30	4	7	-9	17	*23*	cardiosurgical ICU	cardiosurgical ICU; mean age 69 (60-77) years; 21m, 9f	T-Line 200pro™	Tensys Medical Inc., San Diego, CA, USA
Lin, 2017	DBP	4381	23	2,8	6,4	-9,8	15,3	*18,5*	neurosurgery	elective neurosurgery; mean age 53.9±7 years; 14m, 9f	TL-300™	Tensys Medical Inc., San Diego, CA
Meidert, 2014	DBP	2993	24	5	7	-9	19	*22*	ICU	ICU; mean age 67 (54-77) years; 15m, 9f	TL-200™ or TL-200pro™	Tensys Medical Inc., San Diego, CA, USA
Sun, 2017	DBP	1538	30	4,3	7,4	-10,3	18,8	*21,9*	abdominal surgery	colon carcinoma surgery; mean age 61,3 (26-83) years; 15m, 15f	TL-300™	Tensys Medical Inc., San Diego, CA, USA
Szmuk, 2008	DBP	5425	22	1,6	5,6	-8	12	*19,4*	spine surgery	multilevel spine surgery under general anaesthesia with induced hypotension; mean age 48,6 (18-70) years; 10m, 15f	TL100™	Tensys Medical Inc., San Diego, CA, USA
		**245304**	**201**									

	**Applanation tonometry (Vasotrac**®)											
Belani, 1999	MAP	17468	80	-0,2	3	*-6,21*	*5,79*	7	ICU	ICU (critically ill and surgical patients); mean age 55,5 (± 16) years; 47m, 33f	Vasotrac™	Medwave, Arden Hills, MN
Findlay, 2006	MAP	6468	14	5,4	*9,30*	-13,2	24		abdominal surgery	liver transplantation; mean age 48 ± 16 (35-76) years; 10m, 4f	Vasotrac™	Medwave Inc., Arden Hills, MN
Hager, 2009	MAP	40411	22	0,25	*10,43*	-20,6	21,2		bariatric surgery	bariatric surgery; mean age 44,3 ± 9,5 years	Vasotrac™	Medwave, Arden Hills, MN, USA
Thomas, 2004	MAP	307	15	0,1	*4,05*	-8	8,2		ED	patients with arterial pressure measurement; mean age 62 (28-89) years, 9m, 6f	Vasotrac™ APM 205A	Medwave, Danvers, MA
		**64654**	**131**									
	**Applanation tonometry (Vasotrac**®)											
Belani,y 1999	SBP	17468	80	0,02	5,4	*-10,78*	*10,82*	*9,1*	ICU	ICU (critically ill and surgical patients); mean age 55,5 (± 16) years; 47m, 33f	Vasotrac™	Medwave, Arden Hills, MN
Findlay, 2006	SBP	6468	14	7,6	*12,50*	-17,4	32,6		abdominal surgery	liver transplantation; mean age 48 ± 16 (35-76) years; 10m, 4f	Vasotrac™	Medwave Inc., Arden Hills, MN
Hager, 2009	SBP	40411	22	-2,8	*15,25*	-33,3	27,7		bariatric surgery	bariatric surgery; mean age 44,3 ± 9,5 years	Vasotrac™	Medwave, Arden Hills, MN, USA
		**64347**	**116**									
	**Applanation tonometry (Vasotrac**®)											
Belani, 1999	DBP	17468	80	-0,4	3,9	*-8,19*	*7,41*	*11,9*	ICU	ICU (critically ill and surgical patients); mean age 55,5 (± 16) years; 47m, 33f	Vasotrac™	Medwave, Arden Hills, MN
Findlay, 2006	DBP	6468	14	3,3	*7,50*	-11,7	18,3		abdominal surgery	liver transplantation; mean age 48 ± 16 (35-76) years; 10m, 4f	Vasotrac™	Medwave Inc., Arden Hills, MN
Hager, 2009	DBP	40411	22	2,4	*11,30*	-20,2	25		bariatric surgery	bariatric surgery; mean age 44,3 ± 9,5 years	Vasotrac™	Medwave, Arden Hills, MN, USA
		**64347**	**116**									
	**Applanation tonometry (N-CAT**®)											
Searle, 1995, T1	MAP	550	11	-4,1	6,19	*-16,51*	*8,25*		cardiac surgery	elective coronary artery bypass surgery (CABG); mean age 57,1 ± 8,9 years; 10m, 1f; pre-CABG	N-CAT™ N-500	Nellcor, Hayward, CA
Searle, 1995, T2	MAP	550	11	1,5	7,39	*-13,28*	*16,28*		cardiac surgery	elective coronary artery bypass surgery (CABG); mean age 57,1 ± 8,9 years; 10m, 1f; post-CABG	N-CAT™ N-500	Nellcor, Hayward, CA
Searle, 1993	MAP	300	10	0,33	4,2	*-8,07*	*8,73*		ICU	postoperative cardiac patients with rapid atrial fibrilation; mean age 60,8 ± 11,7 years; 7m, 3f	N-CAT™ N-500	Nellcor, Hayward, CA
Siegel, 1994	MAP	88158	16	1,3	9,4	*-17,5*	*20,1*		general surgery	general anesthesia; mean age 54 ± 22 (22-87) years, 6m, 10f	N-CAT™, version 1.0	Nellcor, Hayward, CA
		**89558**	**48**									

	**Applanation tonometry (N-CAT®)**											
Searle, 1995, T1	SBP	550	11	-5,3	6,56	*-18,45*	*7,79*		cardiac surgery	elective coronary artery bypass surgery (CABG); mean age 57,1 ± 8,9 years; 10m, 1f; pre-CABG	N-CAT™ N-500	Nellcor, Hayward, CA
Searle, 1995, T2	SBP	550	11	-5,9	10,17	*-26,25*	*14,43*		cardiac surgery	elective coronary artery bypass surgery (CABG); mean age 57,1 ± 8,9 years; 10m, 1f; post-CABG	N-CAT™ N-501	Nellcor, Hayward, CA
Searle,1993	SBP	300	10	-1,7	4,6	*-10,91*	*8,73*		ICU	postoperative cardiac patients with rapid atrial fibrilation; mean age 60,8 ± 11,7 years; 7m, 3f	N-CAT™ N-500	Nellcor, Hayward, CA
		**1400**	**32**									
	**Applanation tonometry (N-CAT®)**											
Searle, 1995, T1	DBP	550	11	-3,5	8,04	*-19,62*	*12,54*		cardiac surgery	elective coronary artery bypass surgery (CABG); mean age 57,1 ± 8,9 years; 10m, 1f; pre-CABG	N-CAT™ N-500	Nellcor, Hayward, CA
Searle, 1995, T2	DBP	550	11	4,36	6,36	*-8,36*	*17,08*		cardiac surgery	elective coronary artery bypass surgery (CABG); mean age 57,1 ± 8,9 years; 10m, 1f; post-CABG	N-CAT™ N-501	Nellcor, Hayward, CA
Searle, 1993	DBP	300	10	0,99	4,6	*-8,21*	*10,91*		ICU	postoperative cardiac patients with rapid atrial fibrilation; mean age 60,8 ± 11,7 years; 7m, 3f	N-CAT™ N-500	Nellcor, Hayward, CA
		**1400**	**32**									

Table S4: Overview of all included studies - Photoplethysmography (PPG)-technology

literature	reference	n (measurements)	n (patients)	bias	SD	LLOA	ULOA	setting	clinical data and patient characteristics	brand	manufacturer
	**PPG**										
Kachel, 2021, T1	MAP	3970	10	0	*2,75*	-5,5	5,5	ICU	post-cardiac surgery patients at Cardiac Surgery Intensive Care Unit **(wristwatch)**; mean age 60 ± 15 years; 4 f, 6 m	wristwatch	Biobeat Technologies LTD, Petah Tikva, Israel
Kachel, 2021, T2	MAP	3970	10	0	*3,30*	-6,6	6,60	ICU	post-cardiac surgery patients at Cardiac Surgery Intensive Care Unit **(chest-patch)**; mean age 60 ± 15 years; 4 f, 6 m	chest-patch	Biobeat Technologies LTD, Petah Tikva, Israel
Desebbe, 2022	MAP	660	22	0,9	7,00	-13	15	ICU	ICU; mean age 61 (53-65) years; 15 f, 7 m	optical smartphone application for BP measurement (OptiBP™)	Biospectal Inc., Lausanne, Switzerland
Xu, 2022, T1	MAP	280	34	0,49	12,34	-24,5	25,5	elective surgeries	patients >65 undergoing elective surgeries requiring artery catheterizations, **during anesthesia induction**; mean age 72.29 ± 5.40 years; 15f, 26m	NICAP technology	Zhejiang Union Medical Equipment Co., Ltd.
Xu, 2022, T2	MAP	6751	41	2,78	14,17	-25,9	31,46	elective surgeries	patients >65 undergoing elective surgeries requiring artery catheterizations, **BP measurements from all patients**; mean age 72.29 ± 5.40 years; 15f, 26m	NICAP technology	Zhejiang Union Medical Equipment Co., Ltd.
		**15631**	**117**								
	**PPG**										
Chen, 2012	SBP	260	26	2,16	6,23	-10,05	14,4	general surgery	surgery with invasive monitoring; mean age 19 ± 1 years, and 60 ± 1 years; 15m, 11f		
Pellaton, 2019	SBP	326	16	0	7,1	-11,9	12,2	ICU	ICU, mean age 65,7 (16,1) years; 9 f, 14 m	Aktiia OBPM technology	no disclosures
Kachel, 2021, T1	SBP	3998	10	0	*5,05*	-10,1	10,1	ICU	post-cardiac surgery patients at Cardiac Surgery Intensive Care Unit **(wristwatch)**; mean age 60 ± 15 years; 4 f, 6 m	wristwatch	Biobeat Technologies LTD, Petah Tikva, Israel
Kachel, 2021, T2	SBP	3998	10	0	*4,95*	-9,9	9,9	ICU	post-cardiac surgery patients at Cardiac Surgery Intensive Care Unit **(chest-patch)**; mean age 60 ± 15 years; 4 f, 6 m	chest-patch	Biobeat Technologies LTD, Petah Tikva, Israel
Desebbe, 2022	SBP	660	22	0,2	14,00	-26	27	ICU	ICU; mean age 61 (53-65) years; 15 f, 7 m	optical smartphone application for BP measurement (OptiBP™)	Biospectal Inc., Lausanne, Switzerland
Xu, 2022, T1	SBP	280	34	1,72	18,31	-34,3	37,8	elective surgeries	patients >65 undergoing elective surgeries requiring artery catheterizations, **during anesthesia induction**; mean age 72.29 ± 5.40 years; 15f, 26m	NICAP technology	Zhejiang Union Medical Equipment Co., Ltd.
Xu, 2022, T2	SBP	6751	41	2,19	18,86	-34,36	38,74	elective surgeries	patients >65 undergoing elective surgeries requiring artery catheterizations, **BP measurements from all patients**; mean age 72.29 ± 5.40 years; 15f, 26m	NICAP technology	Zhejiang Union Medical Equipment Co., Ltd.

		**16273**	**159**								
	**PPG**										
Chen, 2012	DBP	260	26	-1,49	6,51	-14,26	11,28	general surgery	surgery with invasive monitoring; mean age 19 ± 1 years, and 60 ± 1 years; 15m, 11f		
Chen, 2009	DBP	200	20	1,4	7,5	-13,6	16,4	general surgery	surgical operation; mean age 19 years (5m, 5f), and 60 years (5m, 5f)		
Pellaton, 2019	DBP	326	16	0	2,9	-4,8	5,50	ICU	ICU, mean age 65,7 (16,1) years; 9 f, 14 m	Aktiia OBPM technology	no disclosures
Kachel, 2021, T1	DBP	3979	10	0	*3,30*	-6,6	6,6	ICU	post-cardiac surgery patients at Cardiac Surgery Intensive Care Unit **(wristwatch)**; mean age 60 ± 15 years; 4 f, 6 m	wristwatch	Biobeat Technologies LTD, Petah Tikva, Israel
Kachel, 2021, T2	DBP	3979	10	0	*3,85*	-7,7	7,70	ICU	post-cardiac surgery patients at Cardiac Surgery Intensive Care Unit **(chest-patch)**; mean age 60 ± 15 years; 4 f, 6 m	chest-patch	Biobeat Technologies LTD, Petah Tikva, Israel
Desebbe, 2022	DBP	660	22	1,1	6,00	-11	13	ICU	ICU; mean age 61 (53-65) years; 15 f, 7 m	optical smartphone application for BP measurement (OptiBP™)	Biospectal Inc., Lausanne, Switzerland
Xu, 2022, T1	DBP	280	34	-0,17	12,59	-26,1	25,7	elective surgeries	patients >65 undergoing elective surgeries requiring artery catheterizations, **during anesthesia induction**; mean age 72.29 ± 5.40 years; 15f, 26m	NICAP technology	Zhejiang Union Medical Equipment Co., Ltd.
Xu, 2022, T2	DBP	6751	41	3,27	13,42	-24,31	30,84	elective surgeries	patients >65 undergoing elective surgeries requiring artery catheterizations, **BP measurements from all patients**; mean age 72.29 ± 5.40 years; 15f, 26m	NICAP technology	Zhejiang Union Medical Equipment Co., Ltd.
		**16435**	**179**								

Table S5: Overview of all included studies - one study per technology

literature	reference	n (measurements)	n (patients)	bias	SD	LLOA	ULOA	setting	clinical data and patient characteristics	brand	manufacturer
	**one study per technology (no meta-analysis possible)**										
Li, 2017, T1	MAP	3000	60	0,27	3,64	-6,86	7,4	general surgery	elective surgery under general anesthesia; age 48,67 ± 10,80 (20-65) years; 34m, 26f; beat data	BP100A CNAP system™	Libang Medical Inc., Xi'an, China
Li, 2017, T1	SBP	3000	60	-2,09	5,39	-12,7	8,47	general surgery	elective surgery under general anesthesia; age 48,67 ± 10,80 (20-65) years; 34m, 26f; beat data	BP100A CNAP system™	Libang Medical Inc., Xi'an, China
Li, 2017, T1	DBP	3000	60	2,63	6,44	-9,99	15,26	general surgery	elective surgery under general anesthesia; age 48,67 ± 10,80 (20-65) years; 34m, 26f; beat data	BP100A CNAP system™	Libang Medical Inc., Xi'an, China
Li, 2017, T2	MAP	3600	60	0,5	4,36	-8,05	9,05	general surgery	elective surgery under general anesthesia; age 48,67 ± 10,80 (20-65) years; 34m, 26f; pulse wave data	BP100A CNAP system™	Libang Medical Inc., Xi'an, China
Li, 2017, T2	SBP	3600	60	-2,06	6,51	-14,8	10,7	general surgery	elective surgery under general anesthesia; age 48,67 ± 10,80 (20-65) years; 34m, 26f; pulse wave data	BP100A CNAP system™	Libang Medical Inc., Xi'an, China
Li, 2017, T2	DBP	3600	60	3,06	6,81	-10,3	16,41	general surgery	elective surgery under general anesthesia; age 48,67 ± 10,80 (20-65) years; 34m, 26f; pulse wave data	BP100A CNAP system™	Libang Medical Inc., Xi'an, China
Harju, 2018	MAP	1894	28	11,18	11,1	-12,1	34,2	post-operative care	post-operative setting; mean age 67,2 (39-87) years; 12m, 16f	BPro®	Healthstats, Singapore
Harju, 2018	SBP	1894	28	19,8	16,7	-20,1	59,6	post-operative care	post-operative setting; mean age 67,2 (39-87) years; 12m, 16f	BPro®	Healthstats, Singapore
Harju, 2018	DBP	1894	28	4,8	7,7	-14	23,6	post-operative care	post-operative setting; mean age 67,2 (39-87) years; 12m, 16f	BPro®	Healthstats, Singapore
Gratz, 2017, T1	MAP	3870	24	1,01	5,33	-9,65	11,67	abdominal surgery	major abdominal surgery; mean age 67 (46-83) years; 13m, 11f; 30 min time window comparison	CareTaker®	Empirical Technologies Corporation, Charlottesville, Virginia
Gratz, 2017, T1	SBP	3870	24	-0,57	7,34	-15,3	14,11	abdominal surgery	major abdominal surgery; mean age 67 (46-83) years; 13m, 11f; 30 min time window comparison	CareTaker®	Empirical Technologies Corporation, Charlottesville, Virginia
Gratz, 2017, T1	DBP	3870	24	-2,52	6,47	-15,5	10,42	abdominal surgery	major abdominal surgery; mean age 67 (46-83) years; 13m, 11f; 30 min time window comparison	CareTaker®	Empirical Technologies Corporation, Charlottesville, Virginia
Gratz, 2017, T2	MAP	58701	24	-0,57	5,37	-11,3	10,17	abdominal surgery	major abdominal surgery; mean age 67 (46-83) years; 13m, 11f; data set collected during the entire procedure	CareTaker®	Empirical Technologies Corporation, Charlottesville, Virginia
Gratz, 2017, T2	SBP	58701	24	-2,52	7,24	-17	11,96	abdominal surgery	major abdominal surgery; mean age 67 (46-83) years; 13m, 11f; data set collected during the entire procedure	CareTaker®	Empirical Technologies Corporation, Charlottesville, Virginia
Gratz, 2017, T2	DBP	58701	24	1,02	6,47	-11,9	13,96	abdominal surgery	major abdominal surgery; mean age 67 (46-83) years; 13m, 11f; data set collected during the entire procedure	CareTaker®	Empirical Technologies Corporation, Charlottesville, Virginia

Chou, 2021	SBP	14,674	17	1,92	12,55	-22,7	26,52	general surgery	Surgical patients; mean age 57.35 ± 18.72 years; 6m, 11f;	wireless soft capacitance sensor (CAP)	no disclosures
Chou, 2022	DBP	14,645	17	2,38	12,19	-21,5	26,28	general surgery	Surgical patients; mean age 57.35 ± 18.72 years; 6m, 11f;	wireless soft capacitance sensor (CAP)	no disclosures
Földi, 2019	MAP	12160	21	1,25	2,26	-3,74	6,23	postsurgical care	postsurgical patients; mean age 63,8 ± 11,2 (34-87) years; 15m, 6f;	OnRobot OMD-20-SE-40N 3-axis sensor	OptoForce, OnRobot A/S, Denmark
Földi, 2019	SBP	12160	21	-9,53	4,69	-18,9	-0,15	postsurgical care	postsurgical patients; mean age 63,8 ± 11,2 (34-87) years; 15m, 6f;	OnRobot OMD-20-SE-40N 3-axis sensor	OptoForce, OnRobot A/S, Denmark
Földi, 2019	DBP	12160	21	-0,26	3,06	-7,58	7,19	postsurgical care	postsurgical patients; mean age 63,8 ± 11,2 (34-87) years; 15m, 6f;	OnRobot OMD-20-SE-40N 3-axis sensor	OptoForce, OnRobot A/S, Denmark
Fortin, 2021	MAP	11803	46	-1	7	-14,8	12,7	neurosugery	mean age 56 (25–79) years; 19m, 27f	CNAP2GO	

Table S6: Risk of Bias

A

Volume Clamp (ClearSight™)	RISK OF BIAS						
	Patient selection	Index test	Reference standard	Flow and timing	Multiple measurements per patient taken into account correctly	Check for heterogeneity of variance and dependence of bias on average value of measurement performed	Overall
Balzer (2016)	+	-	-	+	?	?	-
Broch (2013)	+	?	-	?	?	?	-
Fischer (2012)	+	-	-	-	+	?	-
Heusdens (2016)	+	+	+	+	+	?	-
Hofhuizen (2014)	+	-	+	?	+	?	-
Martina (2012)	?	+	+	+	?	?	-
Noto (2019)	+	-	?	+	+	+	-
Rogge (2019)	+	+	+	+	+	?	-
Sakai, (2018)	?	-	?	?	?	?	-
Schramm (2017)	+	-	+	-	+	?	-
Vos (2014)	?	?	-	+	+	+	-
Weiss (2014)	+	+	+	+	+	?	-
Wilde (2016)	+	-	?	+	?	+	-
Yokose (2019	+	?	?	+	+	?	-
Berkelmans (2017)	+	+	+	+	?	+	-
Bugarini (2021)	+	+	+	?	?	?	-
Chachula (2020)	+	+	+	-	?	+	-
Eley (2021)	+	+	+	+	+	+	+
Fassaert (2022)	+	+	+	+	+	+	+
Frank (2021)	+	+	?	-	+	?	-
Hansen (2022)	+	+	+	+	+	?	-
Kho (2022)	?	?	?	+	+	?	-
Lee (2021)	-	+	+	?	?	?	-
Lu (2021)	-	+	+	+	?	?	-
Misugi (2022)	+	+	+	-	+	?	-
Omenas (2020)	+	+	+	+	+	+	+
Schumann (2021)	+	+	+	+	+	+	+
Tadashi (2020)	-	+	?	-	?	-	-

B

Volume Clamp (CNAP™)	RISK OF BIAS						
	Patient selection	Index test	Reference standard	Flow and timing	Multiple measurements per patient taken into account correctly	Check for heterogeneity of variance and dependence of bias on average value of measurement performed	Overall
Araz (2015)	+	?	?	+	?	?	-
Biais (2010)	+	?	+	+	?	?	-
Gayat (2013)	+	-	-	+	+	?	-
Hahn (2012)	+	+	+	+	+	?	-
Huhle (2016)	?	-	?	?	?	+	-
Ilies (2012)	+	+	+	+	+	?	-
Ilies (2015)	+	?	+	+	+	+	-
Jeleazcov (2010)	+	+	+	+	+	+	+
Kumar (2015)	+	-	?	?	?	?	-
Rogge (2018)	+	+	+	+	+	?	-
Sackl-Pietsch (2008)	?	-	-	?	?	+	-
Schramm (2011)	+	+	?	+	?	?	-
Smolle (2015)	+	+	+	+	+	+	+
Wang (2019)	+	-	?	+	+	?	-
Flick (2021)	+	+	+	+	+	?	-

C

Applanation Tonometry	RISK OF BIAS						
	Patient selection	Index test	Reference standard	Flow and timing	Multiple measurements per patient taken into account correctly	Check for heterogeneity of variance and dependence of bias on average value of measurement performed	Overall
Dueck (2012)	+	+	+	+	+	+	+
Greiwe (2018)	+	+	+	+	+	?	-
Greiwe (2016)	+	+	+	+	+	?	-
Janelle (2006)	+	+	?	?	?	?	-
Langwieser (2015)	+	+	+	-	+	+	-
Lin (2017)	+	+	+	-	+	?	-
Meidert (2014)	+	+	+	-	+	+	-
Sun (2017)	-	?	?	-	?	?	-
Szmuk (2008)	+	+	+	+	?	?	-
Searle (1995)	+	+	+	+	?	?	-
Searle (1993)	+	+	+	+	?	?	-
Siegel (1994)[1]	+	-	-	+	?	?	-
Belani (1999)	+	?	?	?	?	?	-
Findlay (2006)	+	+	?	-	?	?	-
Hager (2009)	+	+	+	+	+	-	-
Thomas (2004)	+	?	?	+	?	?	-

[1] This paper did not produce Bland-Altman analyses explicetly but provided relevant data.

D

PPG-technology	RISK OF BIAS						
	Patient selection	Index test	Reference standard	Flow and timing	Multiple measurements per patient taken into account correctly	Check for heterogeneity of variance and dependence of bias on average value of measurement performed	Overall
Chen (2009)	?	?	?	?	-	-	-
Chen (2012)	?	?	?	?	-	-	-
Desebbe (2022)	+	+	+	+	+	-	-
Kachel (2021)	+	?	?	-	?	?	-
Pellaton (2019)	+	?	?	-	?	?	-
Xu (2022)	+	+	+	+	?	?	-

+ low risk - high risk ? unclear risk

Table S7: Meta-analyses summary table

Summary parameters for meta-analysis, model based estimated

							95% CI (Bias)		Lower Limit of agreement with 95% CI					Upper Limit of agreement with 95% CI				
										Model based		Robust variance			Model based		Robust variance	
**Group**	**Group name**	**m**	**Bias**	**SD**	τ^2	I^2	**lower**	**upper**	**Estimate**	**lower**	**upper**	**lower**	**upper**	**Estimate**	**lower**	**upper**	**lower**	**upper**
1	ClearSight MAP	32	3.40	8.96	12.72	86.74	2.00	4.80	-15.89	-18.54	-13.24	-18.84	-12.94	22.69	20.05	25.34	19.74	25.64
2	ClearSight SBP	28	-1.14	13.23	47.65	91.16	-3.88	1.59	-30.99	-35.36	-26.62	-35.61	-26.37	28.71	24.34	33.08	24.09	33.33
3	ClearSight DBP	27	5.76	8.29	19.10	91.53	4.00	7.53	-12.98	-16.05	-9.92	-16.28	-9.69	24.51	21.45	27.57	21.21	27.80
4	CNAP MAP	17	3.89	9.65	9.53	85.85	2.23	5.55	-16.37	-19.61	-13.13	-19.63	-13.11	24.15	20.91	27.39	20.90	27.41
5	CNAP SBP	16	-1.95	14.20	12.18	78.17	-3.97	0.06	-31.19	-35.34	-27.05	-35.38	-27.01	27.29	23.15	31.44	23.11	31.47
6	CNAP DBP	16	6.34	9.48	12.32	89.59	4.46	8.22	-13.87	-17.33	-10.41	-17.60	-10.14	26.55	23.09	30.01	22.82	30.28
7	T-Line MAP	9	2.53	6.60	1.57	50.90	1.34	3.71	-10.92	-14.39	-7.44	-14.45	-7.38	15.97	12.49	19.45	12.44	19.50
8	T-Line SBP	8	0.07	9.15	3.86	57.35	-1.78	1.91	-18.65	-24.02	-13.27	-24.05	-13.24	18.78	13.41	24.15	13.38	24.18
9	T-Line DBP	8	3.09	7.17	0.00	0.00	2.14	4.05	-11.25	-13.51	-8.99	-13.50	-9.00	17.44	15.18	19.70	15.19	19.69
10	Vasotrac MAP	4	0.36	5.97	0.89	40.74	-1.10	1.82	-11.73	-25.28	1.83	-23.67	0.22	12.44	-1.11	26.00	0.50	24.39
11	Vasotrac SBP	3	1.20	10.22	10.95	65.77	-3.42	5.81	-20.28	-54.59	14.02	-50.43	9.86	22.68	-11.63	56.98	-7.47	52.82
12	Vasotrac DBP	3	1.09	7.01	3.00	54.39	-1.51	3.69	-13.35	-36.10	9.40	-33.11	6.41	15.53	-7.23	38.28	-4.24	35.29
13	N-CAT MAP	4	-0.37	6.97	2.99	45.91	-2.89	2.14	-14.73	-23.24	-6.23	-23.21	-6.26	13.98	5.48	22.49	5.51	22.46
14	N-CAT SBP	3	-3.68	7.14	2.04	32.66	-6.46	-0.89	-18.23	-33.42	-3.05	-33.47	-2.99	10.88	-4.31	26.06	-4.36	26.12
15	N-CAT DBP	3	0.84	6.55	8.17	69.50	-3.06	4.74	-13.45	-26.62	-0.27	-26.88	-0.02	15.13	1.95	28.30	1.69	28.56
16	PPG MAP	5	-0.09	6.86	0.00	0.00	-1.20	1.02	-13.80	-24.73	-2.88	-26.06	-1.55	13.63	2.70	24.56	1.38	25.88
17	PPG SBP	7	0.49	9.63	0.00	0.00	-0.85	1.84	-18.78	-29.28	-8.27	-29.28	-8.27	19.76	9.26	30.26	9.26	30.26
18	PPG DBP	8	-0.26	6.38	0.00	0.00	-1.10	0.57	-13.02	-19.14	-6.90	-19.11	-6.93	12.50	6.37	18.62	6.41	18.58

Legend:
m = number of studies included in meta-analysis
τ^2 = Tau squared, variance between studies, measure for heterogeneity
I^2 = I squared, measure for heterogeneity

Table S8: Summary parameters for meta-analysis focusing on bias difference, model based estimated

		Standard DerSimonian Laird							Reduced Model							
					95% CI (Bias)							95% CI (Bias)				
Group	GroupName	m	Bias	SE	Lower limit	Upper limit	τ^2	I^2	m	Bias	SE	Lower limit	Upper limit	τ^2	I^2	Bias difference
1	ClearSight MAP	32	3.40	0.71	2.00	4.80	12.72	86.74	30	2.52	0.58	1.39	3.66	6.97	78.73	-0.88
2	ClearSight SBP	28	-1.14	1.40	-3.88	1.59	47.65	91.16	27	-1.90	1.26	-4.38	0.57	36.27	88.75	-0.76
3	ClearSight DBP	27	5.76	0.90	4.00	7.53	19.10	91.53	26	5.14	0.83	3.51	6.77	15.29	89.90	-0.62
4	CNAP MAP	17	3.89	0.85	2.23	5.55	9.53	85.85								
5	CNAP SBP	16	-1.95	1.03	-3.97	0.06	12.18	78.17								
6	CNAP DBP	16	6.34	0.96	4.46	8.22	12.32	89.59	15	7.13	0.82	5.53	8.74	7.91	85.23	0.79
7	T-Line MAP	9	2.53	0.60	1.34	3.71	1.57	50.90								
8	T-Line SBP	8	0.07	0.94	-1.78	1.91	3.86	57.35	7	0.86	0.62	-0.35	2.06	0.00	0.00	0.79
9	T-Line DBP	8	3.09	0.49	2.14	4.05	0.00	0.00								
10	Vasotrac MAP	4	0.36	0.74	-1.10	1.82	0.89	40.74	1	0.25	2.22	-4.11	4.61	0.00	0.00	-0.11
11	Vasotrac SBP	3	1.20	2.36	-3.42	5.81	10.95	65.77	1	0.02	0.60	-1.16	1.20	0.00	0.00	-1.18
12	Vasotrac DBP	3	1.09	1.33	-1.51	3.69	3.00	54.39	1	2.40	2.41	-2.32	7.12	0.00	0.00	1.31
13	N-CAT MAP	4	-0.37	1.28	-2.89	2.14	2.99	45.91	3	0.76	1.03	-1.25	2.77	0.00	0.00	1.14
14	N-CAT SBP	3	-3.68	1.42	-6.46	-0.89	2.04	32.66	2	-5.50	1.66	-8.76	-2.24	0.00	0.00	-1.82
15	N-CAT DBP	3	0.84	1.99	-3.06	4.74	8.17	69.50	1	0.99	1.45	-1.86	3.84	0.00	0.00	0.15
16	PPG MAP	5	-0.09	0.57	-1.20	1.02	0.00	0.00								
17	PPG SBP	7	0.49	0.69	-0.85	1.84	0.00	0.00	6	-0.28	0.83	-1.91	1.35	0.00	0.00	-0.77
18	PPG DBP	8	-0.26	0.43	-1.10	0.57	0.00	0.00								

Legend:

m = number of studies included in meta-analysis

τ 2 = Tau squared, variance between studies, measure for heterogeneity

I 2 = I squared, measure for heterogeneity

Table S9: Comparison of summary parameters for bias focusing on ratio of CI length, standard versus robust

			Standard DerSimonian Laird						Robust estimation						
				95% CI (Bias)						95% CI (Bias)					
Group	GroupName	m	Bias	Lower limit	Upper limit	Length	τ^2	I^2	Bias	Lower limit	Upper limit	Length	τ^2	I^2	Ratio of CI length
1	ClearSight MAP	32	3.40	2.00	4.80	2.80	12.72	86.74	3.40	1.44	5.36	3.93	12.72	86.74	1.40
2	ClearSight SBP	28	-1.14	-3.88	1.59	5.47	47.65	91.16	-1.14	-4.80	2.52	7.32	47.65	91.16	1.34
3	ClearSight DBP	27	5.76	4.00	7.53	3.53	19.10	91.53	5.76	3.37	8.15	4.78	19.10	91.53	1.35
4	CNAP MAP	17	3.89	2.23	5.55	3.31	9.53	85.85	3.89	1.72	6.06	4.34	9.53	85.85	1.31
5	CNAP SBP	16	-1.95	-3.97	0.06	4.03	12.18	78.17	-1.95	-4.19	0.29	4.49	12.18	78.17	1.11
6	CNAP DBP	16	6.34	4.46	8.22	3.76	12.32	89.59	6.34	3.63	9.05	5.42	12.32	89.59	1.44
7	T-Line MAP	9	2.53	1.34	3.71	2.36	1.57	50.90	2.53	1.29	3.76	2.46	1.57	50.90	1.04
8	T-Line SBP	8	0.07	-1.78	1.91	3.69	3.86	57.35	0.07	-2.20	2.34	4.54	3.86	57.35	1.23
9	T-Line DBP	8	3.09	2.14	4.05	1.91	0.00	0.00	3.09	1.99	4.20	2.21	0.00	0.00	1.16
10	Vasotrac MAP	4	0.36	-1.10	1.82	2.91	0.89	40.74	0.36	-1.51	2.22	3.73	0.89	40.74	1.28
11	Vasotrac SBP	3	1.20	-3.42	5.81	9.24	10.95	65.77	1.20	-9.32	11.71	21.03	10.95	65.77	2.28
12	Vasotrac DBP	3	1.09	-1.51	3.69	5.19	3.00	54.39	1.09	-4.28	6.46	10.73	3.00	54.39	2.07
13	N-CAT MAP	4	-0.37	-2.89	2.14	5.03	2.99	45.91							
14	N-CAT SBP	3	-3.68	-6.46	-0.89	5.57	2.04	32.66							
15	N-CAT DBP	3	0.84	-3.06	4.74	7.79	8.17	69.50							
16	PPG MAP	5	-0.09	-1.20	1.02	2.22	0.00	0.00							
17	PPG SBP	7	0.49	-0.85	1.84	2.69	0.00	0.00	0.49	-1.43	2.41	3.84	0.00	0.00	1.42
18	PPG DBP	8	-0.26	-1.10	0.57	1.67	0.00	0.00	-0.26	-1.20	0.68	1.89	0.00	0.00	1.13

Legend:

m = number of studies included in meta-analysis

τ 2 = Tau squared, variance between studies, measure for heterogeneity

I 2 = I squared, measure for heterogeneity
