# Supplementary material for: Accuracy and precision of continuous, non‐invasive blood pressure devices: a systematic review and meta‐analyses
Source: Anaesthesia. 2026 Jun 15;81(11):1581–92. doi: 10.1111/anae.70250 (PMC13659784; doi:10.1111/anae.70250)
Supplement: Supplementary file 3 [file ANAE-81-1581-s004.pdf]

Appendix S2: Statistical Code

````
---
title: "Accuracy and precision of continuous, noninvasive blood pressure devices: A
systematic review and meta-analyses including six technologies."
author: Irene  Schmidtmann</br> Institut für Medizinische Biometrie, Epidemiologie
  und Informatik (IMBEI)</br> University Medical Center, Mainz</br>
Irene.Schmidtmann@uni-mainz.de</br>
date: "`r format(Sys.time(), '%d-%m-%Y %H:%M %Z')`"
output:
  html_document:
    code_folding: hide
    toc: yes
    toc_float: yes
    toc_depth: 3
    collapsed: cosmo
    number_sections: yes
    theme: lumen
subtitle: Meta analysis project Stefanie Lischker
editor_options:
  markdown:
    wrap: 72
---

```{r setup, message = FALSE}
# load necessary libraries
knitr::opts_chunk$set(echo = TRUE)
library("knitr")
library("haven")
library("kableExtra")
library("dplyr")
library("metafor")
library("grid")
library("forestplot")
library("arsenal")
library("ggplot2")
library("writexl")

# Determine date & time
system_time <- Sys.time()
Date <- paste0(substr(system_time, 1, 4),
               substr(system_time, 6, 7),
               substr(system_time, 9, 10))
# Set paths for all Code chunks
knitr::opts_knit$set(root.dir = "Z:/Beratung/Lischker/Programs")
path <- "Z:/Beratung/Lischker/Programs"
setwd(path)
output_directory <- "../Output/"
data_directory <- "../Data/"

fig_num <- 0
table_num <- 1

```
````

Purpose

This is a meta-analysis of Bland-Altman analyses comparing MAP, SBP and DBP using six technologies. The analysis is based on Tipton E, Shuster J. A framework for the meta-analysis of Bland--Altman studies based on a limits of agreement approach. Statistics in Medicine, 2017, 36, 3621-3635.

The main functions used are taken from the supplementary material accompanying this paper.

```
```{r Define_Functions, message = FALSE}
# Use functions provided by Tipton & Shuster
# ---------------------------------------------------------------
# Functions for calculating and comparing Bland-Altman Meta-analyses
# ---------------------------------------------------------------

meta <- function(Te, V_T) {
        #Te          # vector of estimates (either logS^2 (log variance) or bias)
        #V_T         # vector of conditional variances
        #method      # RE or RVE

        m <- length(Te)                    # number of studies
        wt_FE <- 1/V_T                     # initial weights (ignoring
                                  # heterogenity)
        T_FE <- sum(Te*wt_FE)/sum(wt_FE)      # preliminary pooled estimate for bias
                                  # or log(S^2)
        Q <- sum(wt_FE*(Te - T_FE)^2)        # initial Cochran's Q-statistic
        S1 <- sum(wt_FE)
        S2 <- sum(wt_FE^2)
        o2 <- max(0,(Q - (m - 1))/(S1 - S2/S1))
                                  # between-study heterogeneity in bias
                                        # or log(S^2)
        wt_RE <- 1/(V_T + o2)              # updated weights which now include
                                        # between-study variance
        T_RE <- sum(Te*wt_RE)/sum(wt_RE)      # pooled estimate for bias or log(S^2)
                                  # based on updated weights
        V_T_RE_mod <- 1/sum(wt_RE)           # model based variance of pooled estimate
        V_T_RE_rve <- (m/(m - 1))*sum(wt_RE^2*(Te - T_RE)^2)/(sum(wt_RE))^2
                                  # robust variance estimate for pooled
                                  # estimate of bias or log(S^2)

  result_list <- list(m, T_RE, o2, V_T_RE_mod, V_T_RE_rve, wt_RE)
  names(result_list) <- c("m", "T_RE", "o2", "V_T_RE_mod", "V_T_RE_rve", "wt_RE")

  return(result_list)

}

loa_maker <- function(bias, V_bias, logs2, V_logs2) {
  # bias      # vector of bias estimates
  # V_bias    # vector of variances of bias estimates
  # logs2     # vector of log(S^2) of bias - from studies
  # V_logs2   # vector of variance of log(S^2)

        bias_row <- meta(bias,V_bias)         # obtain pooled bias and corresponding
                                  # variance estimates
```
```

```
        logs2_row <- meta(logs2,V_logs2)       # obtain pooled log(S^2)and corresponding
                                  # variance estimates

        bias_mean <- bias_row[["T_RE"]]        # pooled bias
        sd2_est <- exp(logs2_row[["T_RE"]])    # pooled within study variance
        tau_est <- max(0, bias_row[["o2"]])    # between study variance
        V_T_RE_mod <- bias_row[["V_T_RE_mod"]] # variance of pooled bias
        V_T_RE_rve <- bias_row[["V_T_RE_rve"]] # robust variance of pooled bias

        LOA_L <- bias_mean - 2*sqrt(sd2_est + tau_est) # pooled lower limit of agreement
        LOA_U <- bias_mean + 2*sqrt(sd2_est + tau_est) # pooled upper limit of agreement

        m <- bias_row[["m"]]                   # number of patients in study
        tcrit <- qt(1 - .05/2, m - 1)          # percentile of the t distribution with m-1 df
        z <-  qnorm(1 - .05/2, 0, 1)           # percentile of the standard normal distribution

  CI_biasL_mod <- bias_mean - z * sqrt(bias_row[["V_T_RE_mod"]])
                              # 95% CI of bias, lower limit
  CI_biasU_mod <- bias_mean + z * sqrt(bias_row[["V_T_RE_mod"]])
                              # 95% CI of bias, upper limit

        B1 <- sd2_est^2/(sd2_est + tau_est)
        # B2 <- tau_est^2/(sd2_est + tau_est)  # This differs from the paper
        B2 <- 1/(sd2_est + tau_est)            # This is, what the paper requires

        wt <- 1/V_bias
#       S1 <- sum(wt)
#       S2 <- sum(wt^2)
#       S3 <- sum(wt^3)
#       A0 <- 2*(m-1)/(S1-S2/S1)^2
#       A1 <- 4/(S1 - S2/S1)
#       A2 <- 2*(S2-2*S3/S1+S2^2/S1^2)/(S1-S2/S1)^2
#       V_logT2 <- A0/tau_est^2 + A1/tau_est + A2

        V_logT2 <- 2/sum((V_bias + tau_est)^(-2))
                                  # variance of Tau^2 (formula 4.13)

        V_LOA_mod <- bias_row[["V_T_RE_mod"]] + B1*logs2_row[["V_T_RE_mod"]] +
B2*V_logT2
                                  # model based variance of pooled LOA
                                  # (using formula (4.16)
        V_LOA_rve <- bias_row[["V_T_RE_rve"]] + B1*logs2_row[["V_T_RE_rve"]] +
B2*V_logT2
                                  # robust  variance of pooled LOA
                                  # (using formula (4.16)

        CI_LL_mod <- LOA_L - tcrit*sqrt(V_LOA_mod)
        CI_LU_mod <- LOA_L + tcrit*sqrt(V_LOA_mod)
        CI_UL_mod <- LOA_U - tcrit*sqrt(V_LOA_mod)
        CI_UU_mod <- LOA_U + tcrit*sqrt(V_LOA_mod)

        CI_LL_rve <- LOA_L - tcrit*sqrt(V_LOA_rve)
        CI_LU_rve <- LOA_L + tcrit*sqrt(V_LOA_rve)
        CI_UL_rve <- LOA_U - tcrit*sqrt(V_LOA_rve)
        CI_UU_rve <- LOA_U + tcrit*sqrt(V_LOA_rve)
```

```
        result_list <- list(m, bias_mean, sqrt(sd2_est), V_T_RE_mod, V_T_RE_rve,
                            tau_est, LOA_L, LOA_U,
                            CI_biasL_mod, CI_biasU_mod,
                            CI_LL_mod, CI_LU_mod, CI_UL_mod, CI_UU_mod,
                            CI_LL_rve, CI_LU_rve, CI_UL_rve, CI_UU_rve,
                            bias_row[["wt_RE"]])
        names(result_list) <- c("m", "bias_mean", "SD", "V_T_RE_mod", "V_T_RE_rve",
                                "tau_est", "LOA_L", "LOA_U",
                                "CI_biasL_mod", "CI_biasU_mod",
                                "CI_LL_mod", "CI_LU_mod", "CI_UL_mod", "CI_UU_mod",
                                "CI_LL_rve", "CI_LU_rve", "CI_UL_rve", "CI_UU_rve",
                                "wt_RE")

        return(result_list)

}

# Function to perform meta-analysis and generate plots ----
make_meta_plots <- function(subdat, group_name,
                            idx,
                            save_forest = TRUE,
                            outdir = output_directory) {

  # Obtain Random effects metaanalysis using the DerSimonian Laird estimator
  # rma returns the same values for summary Bias and Variance as obtained
  # with loa_maker, but results can be used directly in function forest for
  # forest plots
  m_result_DL <- rma(Bias, V_bias, slab = Study, ni = n,
                     data = subdat, method = "DL")

  # Robust method fails for groups 13 to 18
  if (idx <= 12 | idx >= 17){
    m_result_robust <- robust(m_result_DL, cluster=author,
                              adjust=TRUE, clubSandwich=FALSE)
  }else{
    m_result_robust <- NA
  }
  # Obtain I^2
  I2[i] <- m_result_DL$I2

  # Capture influence measures
  my_inf <- influence(m_result_DL)

  # Save results to matrix
  result_list <- loa_maker(subdat$Bias, subdat$V_bias,
                           subdat$logs2, subdat$V_logs2)
  result_vector <- unlist(result_list)[1:18]

  # Axis boundaries
  boundary <- max(abs(subdat$CI_LL), abs(subdat$CI_UU))
  my_step <- dplyr::case_when(
    boundary <= 40 ~ 5,
    boundary <= 80 ~ 10,
    TRUE ~ 20
  )
  boundary <- my_step * ceiling(boundary / my_step)
```

```
my_at    <- seq(-boundary, boundary, my_step)
my_xlim  <- c(-4*boundary, 2*boundary)

# Weights and labels
weights <- paste0(my_inf$inf$inf, " ", fmtx(weights(m_result_DL), digits = 1), "%")
current_ilab <- data.frame(weights, subdat$n, subdat$n_Messungen)
my_psize  <- 1.2 * weights(m_result_DL) / max(weights(m_result_DL))

# Title
cat(c("\n\n##", group_name, "\n\n"))
cat("\n\n### Forest plot\n\n")

# Open device if saving

op <- par()
f_options <- forest(m_result_DL,
                    slab = Study,
                    ilab = current_ilab,
                    ilab.xpos = c(-2*boundary, -1.5*boundary, -boundary),
                    ilab.pos = 2,
                    xlab = "Bias and limits of agreement",
                    xlim = my_xlim,
                    at = my_at,
                    ylim = c(-1.5, m_result_DL$k + 3),
                    psize = my_psize, lty = "solid",
                    efac = 1.5,
                    header = FALSE, shade = "zebra")

par(xpd = NA)
par(cex = f_options$cex, font = 2)

# Add labels and heterogeneity text
text(
  f_options$ilab.xpos,
  m_result_DL$k + 2,
  c("Weight", parse(text = "n[subjects]"), parse(text = "n[measurements]")),
  col = "black",
  pos = 2
)
text(f_options$xlim,
     m_result_DL$k + 2,
     pos = c(4, 2),
     c(paste0("Study (", group_name, ")"), "Bias [95% CI]"))

# Add tau squared and other measures of heterogeneity
text(f_options$xlim[1], -3, pos = 4,
     bquote(paste("Heterogeneity: ",
                  "Tau"^2, " = ", .(fmtx(m_result_DL$tau2, digits = 2)), "; ",
                  "Chi"^2, " = ", .(fmtx(m_result_DL$QE, digits = 2)),
                  ", df = ", .(m_result_DL$k - m_result_DL$p),
                  " (", .(fmtp(m_result_DL$QEp, digits = 2, pname = "P",
                               add0 = TRUE, sep = TRUE, equal = TRUE)),
                  "); ", I^2, " = ", .(round(m_result_DL$I2)), "%")))


### superimpose forest plot for lower limits of agreement
```

```
par(new = TRUE)
with(current.subset, forest(LLOA, ci.lb = CI_LL, ci.ub = CI_LU, col = "darkgrey",
                            xlim = f_options$xlim, ylim = f_options$ylim,
                            alim = f_options$alim,
                            annotate = FALSE, refline = NA, slab = NA,
                            psize = my_psize, xaxt = "n", xlab = "", lty = "solid"))
poly_LOA_x <- result_vector[c("LOA_L", "CI_LL_rve", "LOA_L", "CI_LU_rve", "LOA_L")]
poly_LOA_y <- c(0.5, 0, -0.5, 0, 0.5) - 1
polygon(poly_LOA_x, poly_LOA_y, col = "darkgrey", border = "darkgrey")


### superimpose forest plot for upper limits of agreement
par(new = TRUE)
with(current.subset, forest(ULOA, ci.lb = CI_UL, ci.ub = CI_UU, col = "darkgrey",
                            xlim = f_options$xlim, ylim = f_options$ylim,
                            alim = f_options$alim,
                            annotate = FALSE, refline = NA, slab = NA,
                            psize = my_psize, xaxt = "n", xlab = "", lty = "solid"))
poly_LOA_x <- result_vector[c("LOA_U", "CI_UL_rve", "LOA_U", "CI_UU_rve", "LOA_U")]
poly_LOA_y <- c(0.5, 0, -0.5, 0, 0.5) - 1
polygon(poly_LOA_x, poly_LOA_y, col = "darkgrey", border = "darkgrey")


p <- recordPlot()
if (save_forest) {
  if (!dir.exists(outdir)) dir.create(outdir)
  my_height <- 6 + 0.75 * nrow(subdat)
  jpeg(file.path(outdir, paste0("forest_plot_", idx, "_", group_name, ".jpg")),
       width = 30, height = my_height, units = "cm", res = 300)

  replayPlot(p)

dev.off()
}

# reset plot parameters
par(op)


# Funnel plot
cat("\n\n### Funnel plot\n\n")
funnel(m_result_DL,
       xlim = c(-max(abs(m_result_DL$yi)), max(abs(m_result_DL$yi))),
       ylab = "Standard Error", xlab = "Observed Bias")

# Baujat plot
cat("\n\n### Baujat plot\n\n")
baujat(m_result_DL,  ylim=c(0,1), bty="l", las=1, symbol = "slab")

# Influence plot
cat("\n\n### Influence plot\n\n")
plot(my_inf)

flagged <- which(my_inf$is.infl)

# Sensitivity analysis
```

```
  if (length(flagged) > 0) {
    reduced_data <- subdat |>
      filter(row_number() %nin% flagged)

    # "Refitting model without influential studies
    m_result_DL_sens <- rma(Bias, V_bias, slab = Study, ni = n,
                            data = reduced_data, method = "DL")
    influential <- TRUE
  }else{
    m_result_DL_sens <- NA
    influential <- FALSE
  }

  invisible(list(model = m_result_DL,
                 robust_model = m_result_robust,
                 sens_model = m_result_DL_sens,
                 influential = influential,
                 influence = my_inf,
                 results = result_list))
}
```

Import data

Data were originally provided in an Excel file (Metaanalysenliste März 23.xlsx, corrected version obtained on October 25th, 2023 and further corrected on January 4th/5th, 2024) and have been pre-processed using SAS. The resulting SAS dataset (lischker_studies_only_20240105.sas7bdat) is imported.

Changes that have been made in the SAS data set relative to the original Excel file. These changes are only technical and don't have to be included in the methods section of the paper.

- Only data lines containing study data were kept. Lines with pooled data were removed.

- StudyNr was added - alphabetically sorted by first authors within each group

- Text for Groups was added.

- Depending on the sign of the original bias, upper and lower limits of agreement had to be multiplied by -1 and then exchanged.

```{r Import_Data, message = FALSE}
lischker_studies_only_20240104 <- read_sas(paste0(data_directory,
                                       "/lischker_studies_only_20240104.sas7bdat"),
                                       NULL)  |>
  mutate(author = sub(",.*", "", Study), .before = Study)

# Select columns that are relevant to analysis and obtain input vectors for meta
# analysis functions

# bias = vector of study specific bias estimates
# V_bias = vector of variances of study specific bias estimates (i.e., s2*/n)
```

```
# logs2 = vector of study specific estimates, where logs2 = log(s2*) + 1/(n-1) and s2* is bias adjusted
# V_logs2 = vector of variances (i.e., 2/(n-1))
studies <- lischker_studies_only_20240104 %>%
  select(., Group, GroupName, StudyNr, author, Study, Reference, n, n_Messungen, Bias, SD, LLOA, ULOA, lfd) %>%
  mutate(., t_LOA = qt(0.975, n - 1)*sqrt(3/n)*SD,
         SE = SD / sqrt(n),
         CI_LL = LLOA - t_LOA,
         CI_LU = LLOA + t_LOA,
         CI_UL = ULOA - t_LOA,
         CI_UU = ULOA + t_LOA,
         V_bias = SD^2 / n,
         raw_weight = n / SD^2,
         logs2 = log(SD^2) + 1 / (n - 1),
         V_logs2 = 2 / (n - 1))
```

Meta analysis

Using the method of Tipton and Shuster, sampling distributions of the population limits of agreement were obtained. Random effects meta analysis was performed, the DerSimonian-Laird estimator was used. Hetereogeneity is measured by τ^2 and I^2 - these relate to the mean difference between methods ("bias").

The results are diplayed in forest plots.

Funnel plots have been produced to allow visual assesement of heterogeneity and potential reporting (publication) bias.

- In Funnel plots the standard deviation is plotted versus the effect size (here: Bias (in "Bland Altman" language)). The lines depict 95% confidence intervals. If more than 5% of studies are outside the funnel lines, there is indication of heterogeneity.

- If Funnel plots have been produced to assess potential publication bias: if the studies are asymmetric, this may indicate reporting bias.

```{r Meta_analysis_prepare, message = FALSE, warning = FALSE, results="asis"}
# Prepare Groups of studies and objects for results ----
df.Groups <- studies %>%
  select(., Group, GroupName) %>%
  unique(.)

result_matrix <- matrix(data = NA,
                        nrow = nrow(df.Groups),
                        ncol = 18)
colnames(result_matrix) <- c("m", "Bias", "SD", "V_T_RE_mod", "V_T_RE_rve",
                             "tau^2","LOA_L","LOA_U",
                                   "CI_biasL_mod", "CI_biasU_mod",
                                   "CI_LL_mod", "CI_LU_mod", "CI_UL_mod", "CI_UU_mod",
                                   "CI_LL_rve", "CI_LU_rve", "CI_UL_rve", "CI_UU_rve")
model_se <- rep(NA, 18)
I2 <- rep(NA, 18)
```

```
result_matrix_robust <- matrix(data = NA,
                               nrow = nrow(df.Groups),
                               ncol = 7)
colnames(result_matrix_robust) <- c("m_r",
                                    "Bias_r",
                                    "SE_r",
                                    "tau^2_r",
                                    "I^2__r",
                                    "CI_biasL_rob_r",
                                    "CI_biasU_rob_r")

result_matrix_sens<- matrix(data = NA,
                            nrow = nrow(df.Groups),
                            ncol = 7)
colnames(result_matrix_sens) <- c("m_se",
                                  "Bias_se",
                                  "SE_se",
                                  "tau^2_se",
                                  "I^2__se",
                                  "ci.lb",
                                  "ci.ub")


out <- list()
```

```{r Meta_analysis_by_group_1_18, message = FALSE, warning = FALSE, results="asis", fig.width = 12, fig.height=12}

for (i in unique(studies$Group)[1:18]) {
  current.subset <- dplyr::filter(studies, Group == i)
  out[[i]] <- make_meta_plots(current.subset,
                              df.Groups$GroupName[i],
                              i,
                              save_forest = TRUE)
  result_matrix[i, ] <- unlist(out[[i]]$results)[1:18]
  I2[i] <- out[[i]][["model"]][["I2"]]
  model_se[i] <- out[[i]][["model"]][["se"]]

  if (i <= 12 | i >= 17) {
    result_matrix_robust[i,] <-  c(out[[i]][["robust_model"]][["k"]],
                                   out[[i]][["robust_model"]][["b"]][, 1],
                                   out[[i]][["robust_model"]][["se"]],
                                   out[[i]][["robust_model"]][["tau2"]],
                                   out[[i]][["robust_model"]][["I2"]],
                                   out[[i]][["robust_model"]][["ci.lb"]],
                                   out[[i]][["robust_model"]][["ci.ub"]]
    )}

  if(out[[i]][["influential"]]){
    result_matrix_sens[i,] <-  c(out[[i]][["sens_model"]][["k"]],
                                 out[[i]][["sens_model"]][["b"]][, 1],
                                 out[[i]][["sens_model"]][["se"]],
                                 out[[i]][["sens_model"]][["tau2"]],
```

````
                              out[[i]][["sens_model"]][["I2"]],
                              out[[i]][["sens_model"]][["ci.lb"]],
                              out[[i]][["sens_model"]][["ci.ub"]]
                              )
  }
}
```

# Meta analysis summary
## Table

```{r Meta_analysis_summary_table_1, message = FALSE, warning = FALSE, results="asis"}
# rownames(result_matrix) <- unique(studies$Group)

# agreement (LOA)
# LOA_L = LOA lower bound
# LOA_L = LOA upper bound
# model-based random-effects meta-analysis estimate of 95% CI for LOA
# CI_LL_mod = lower CI limit for Lower Limit of agreement
# CI_LU_mod = upper  CI limit for Lower Limit of agreement
# CI_UL_mod = lower CI limit for Upper Limit of agreement
# CI_UU_mod = upper  CI limit for Upper Limit of agreement
# robust variance estimation meta-analysis estimate of CI for LOA
# CI_LL_rve = lower CI limit for Lower Limit of agreement
# CI_LU_rve = upper  CI limit for Lower Limit of agreement
# CI_UL_rve = lower CI limit for Upper Limit of agreement
# CI_UU_rve = upper  CI limit for Upper Limit of agreement

result_df <- cbind(df.Groups, as.data.frame(result_matrix), I2) %>%
  select(!(c(V_T_RE_mod,     V_T_RE_rve))) %>%
  relocate(c(CI_biasL_mod, CI_biasU_mod), .before = LOA_L) %>%
  relocate(c(CI_LL_mod, CI_LU_mod, CI_LL_rve, CI_LU_rve), .after = LOA_L) %>%
  relocate(c(CI_UL_mod, CI_UU_mod, CI_UL_rve, CI_UU_rve), .after = LOA_U) %>%
  relocate(I2, .before = CI_biasL_mod)



# Display result as table
  table1 <- kbl(
    result_df,
    caption = "*Summary parameters for metaanalysis, model based estimated*",
    col.names = c("Group", "Group name", "m", "Bias", "SD", "$\\tau^2$", "$I^2$",
                  "lower", "upper",
                  "Estimate", "lower", "upper", "lower", "upper",
                  "Estimate", "lower", "upper", "lower", "upper"),
    row.names = FALSE,
    digits = 2,
    escape = F
  ) %>%
    kable_styling("striped", full_width = F, position = "left") %>%
    add_header_above(c(rep(" ", 10),
                       "Model based" = 2,
                       "Robust variance" = 2,
                       " ",
                       "Model based" = 2,
                       "Robust variance" = 2)) %>%
````

    add_header_above(c(rep(" ", 7),
                  "95% CI (Bias)" = 2,
                  "Lower Limit of agreement with 95% CI" = 5,
                  "Upper Limit of agreement with 95% CI" = 5))
  print(table1)

```

**Legend:**\
m = number of studies included in metaanalysis,\
$\tau^2$ = Tau squared, variance between studies, measure for heterogeneity,\
$I^2$ = I squared, measure for heterogeneity,\
LOA_L, LOA_U = LOA lower and upper limits of agreement\
*Limits of agreement (LOA)*\
LOA_L = LOA lower bound\
LOA_L = LOA upper bound\
*Model-based random-effects meta-analysis estimate of 95% CI for LOA*\
CI_LL_mod = lower CI limit for Lower Limit of agreement\
CI_LU_mod = upper CI limit for Lower Limit of agreement\
CI_UL_mod = lower CI limit for Upper Limit of agreement\
CI_UU_mod = upper CI limit for Upper Limit of agreement\
*Robust variance estimation meta-analysis estimate of CI for LOA*\
CI_LL_rve = lower CI limit for Lower Limit of agreement\
CI_LU_rve = upper CI limit for Lower Limit of agreement\
CI_UL_rve = lower CI limit for Upper Limit of agreement\
CI_UU_rve = upper CI limit for Upper Limit of agreement

CI_LL_mod, CI_UU_mod = model-based random-effects meta-analysis estimate
of 95% CI for LOA, given by lower limit of 95% CI for LOA_L and upper
limit of 95% CI for LOA_U\
CI_LL_rve, CI_UU_rve = robust variance estimation meta-analysis estimate
of 95% CI for LOA given by lower limit of 95% CI for LOA_L and upper
limit of 95% CI for LOA_U

## Comparison standard versus robust estimates
Robust estimates could not be computed for groups 13 to 16. In these
groups there were too few studies to perform the robust estimation.

Groups 13, 14, 15 address N-CAT. These include 3 studies by Searle et al,
2 of which are in the same paper. Only for MAP there is a study from a
different group.

Group 16 addresses MAP measurement with PPG technology. This analysis
contains only 5 Studies, among them are 2 by Kachel et al from a single paper
and 2 from Xu et al from a single
paper.

```{r Meta_analysis_summary_table_2, message = FALSE, warning = FALSE, results="asis"}
# Combine standard and robust estimates in table

result_df_robust <- cbind(df.Groups, as.data.frame(result_matrix_robust))

result_comparison <- result_df |>
  select(Group, GroupName, m, Bias, CI_biasL_mod, CI_biasU_mod, `tau^2`, I2) |>
  left_join(result_df_robust |>

~~~
            select(Group, GroupName, Bias_r, CI_biasL_rob_r, CI_biasU_rob_r, `tau^2_r`,
`I^2__r`)) |>
  mutate(CI_length = CI_biasU_mod - CI_biasL_mod, .after = CI_biasU_mod) |>
  mutate(CI_length_r = CI_biasU_rob_r - CI_biasL_rob_r, .after = CI_biasU_rob_r) |>
  mutate(ratio = CI_length_r/CI_length)


# Display result as table
  table2 <- kbl(
    result_comparison,
    caption = "*Comparison of summary parameters for bias, standard versus robust*",
    col.names = c("Group", "Group name", "m",
                  "Bias", "lower", "upper", "length", "$\\tau^2$", "$I^2$",
                  "Bias", "lower", "upper", "length", "$\\tau^2$", "$I^2$",
                  "Ratio of lengths"),
    row.names = FALSE,
    digits = 2,
    escape = F
  ) %>%
    kable_styling("striped", full_width = F, position = "left") %>%
    add_header_above(c(rep(" ", 4),
                       "95% CI (Bias)" = 3,
                       rep(" ", 3),
                       "95% CI (Bias)" = 3,
                       rep(" ", 3))) %>%
    add_header_above(c(rep(" ", 3),
                     "Standard DerSimonian Laird" = 6,
                     "Robust estimation" = 6,
                     "Ratio"))
  print(table2)

  write_xlsx(result_comparison, paste0(data_directory, Date, " Compare standard and
robust.xlsx"), format_headers = TRUE)
```
~~~

Comparison full versus reduced model
If influential studies were detected, the model is re-fit without the influential
studies as sensitivity analysis. Full and reduced models are shown side by side.

If no influential studies were detected, the corresponding line is filled with NA.

~~~
```{r Meta_analysis_summary_table_3, message = FALSE, warning = FALSE, results="asis"}
# Combine full and reduced estimates in table
compare_full_reduced <- cbind(df.Groups, as.data.frame(result_matrix), I2, model_se,
                              as.data.frame(result_matrix_sens)) |>
  select(Group, GroupName,
       m, Bias, model_se, CI_biasL_mod, CI_biasU_mod, `tau^2`, I2,
       m_se, Bias_se, SE_se, ci.lb, ci.ub, `tau^2_se`, `I^2__se`) |>
  mutate(diff_Bias = Bias_se - Bias)


table3 <- kbl(
    compare_full_reduced,
    caption = "*Summary parameters for metaanalysis, model based estimated*",
    col.names = c("Group", "Group name",
                  "m", "Bias", "SE", "lower", "upper", "$\\tau^2$", "$I^2$",
~~~

```
              "m", "Bias", "SE", "lower", "upper", "$\\tau^2$", "$I^2$",
              "Bias difference"),
    row.names = FALSE,
    digits = 2,
    escape = F
  ) %>%
    kable_styling("striped", full_width = F, position = "left") %>%
    add_header_above(c(rep(" ", 5),
                    "95% CI (Bias)" = 2,
                    rep(" ", 5),
                    "95% CI (Bias)" = 2,
                    rep(" ", 3))) %>%
    add_header_above(c(rep(" ", 2),
                  "Standard DerSimonian Laird" = 7,
                  "Reduced Model" = 7,
                   rep(" ", 1)))

print(table3)

 write_xlsx(compare_full_reduced, paste0(data_directory, Date, " Compare Full vs
reduced.xlsx"), format_headers = TRUE)
```

Figure

```{r Meta_analysis_summary_figure, echo = FALSE, message = FALSE, warning = FALSE,
results="asis", fig.width = 12, fig.height=12}
result_tbl <- as_tibble(result_df)

p2 <- ggplot(result_tbl, aes(y = GroupName)) +
  # Lower LOA intervals
  geom_errorbar(
    aes(xmin = CI_LL_rve, xmax = CI_LU_rve),
    color = "darkgrey",
    width = 0.2,
    size = 0.7
  ) +
  geom_point(
    aes(x = LOA_L),
    color = "darkgrey",
    shape = 23,
    fill = "darkgrey",
    size = 3
  ) +

  # Upper LOA intervals
  geom_errorbar(
    aes(xmin = CI_UL_rve, xmax = CI_UU_rve),
    color = "darkgrey",
    width = 0.2,
    size = 0.7
  ) +
  geom_point(
    aes(x = LOA_U),
    color = "darkgrey",
    shape = 23,
```

```
    fill = "darkgrey",
    size = 3
  ) +

  # Bias intervals
  geom_errorbar(
    aes(xmin = CI_biasL_mod, xmax = CI_biasU_mod),
    color = "black",
    width = 0.2,
    size = 0.7
  ) +
  geom_point(
    aes(x = Bias),
    color = "black",
    shape = 23,
    fill = "black",
    size = 3
  ) +

  geom_vline(xintercept = 0, linetype = "dashed") +
  scale_y_discrete(name = "", limits = rev(result_tbl$GroupName)) +
  scale_x_continuous(limits = c(-60, 60), breaks = seq(-60, 60, 10)) +
  labs(x = "Bias and Limits of Agreement (with 95% CI)") +
  theme_minimal(base_size = 12) +
  theme(
    panel.grid = element_blank(),
    axis.line = element_line(color = "black"),
    axis.ticks = element_line(color = "black"),
    axis.ticks.length = unit(0.25, "cm"),
    axis.text = element_text(family = "Avenir Next"),
    axis.title = element_text(family = "Avenir Next")
  )


jpeg(file.path(output_directory, "summary_forest_plot.jpg"),
        width = 20, height = 20, units = "cm", res = 300)
p2
dev.off()

p2

```

Program Info

Reference for R and used packages

```{r Packages}
citation()

citation("knitr")
citation("haven")
citation("kableExtra")
citation("dplyr")
citation("metafor")
citation("forestplot")
```

````
```

## SessionInfo

```{r sessionInfo}
sessionInfo()
```

-----


ods html close; ods html;
%let filename=Lischker Daten einlesen und aufbereiten; /* Hier den Dateinamen angeben */
dm wpgm  "file '&date &filename..sas'";
dm log "clear text"; /* löscht Log-Fenster */
dm output "clear text"; /* löscht Output-Fenster */


/* Daten für Meta-Analyse Lischker einlesen und aufbereiten
   Einige Korrekturen gegenüber Version vom Oktober 2023 vorgenommen.

   Autor: Irene Schmidtmann, IMBEI
   Stand: 05.01.2024 14:15
*/

ods rtf file="&o\&date &filename..rtf"
        style=statistical fontscale=85
        nogfootnote nogtitle
        contents toc_data
        ;
proc format library=library;
value Methode (max=56)
       1 = "ClearSight MAP"
       2 = "ClearSight SBP"
       3 = "ClearSight DBP"
       4 = "CNAP MAP"
       5 = "CNAP SBP"
       6 = "CNAP DBP"
       7 = "T-Line MAP"
       8 = "T-Line SBP"
       9 = "T-Line DBP"
      10 = "Vasotrac MAP"
      11 = "Vasotrac SBP"
      12 = "Vasotrac DBP"
      13 = "N-CAT MAP"
      14 = "N-CAT SBP"
      15 = "N-CAT DBP"
      16 = "PPG MAP"
      17 = "PPG SBP"
      18 = "PPG DBP"
      ;
value Meta
      1 = "single study"
      2 = "pooled"
      ;
````
````

```
value mypercent
   low - high = [percent7.1]
            . = " "
            ;
run;

/* Korrigierte Daten einlesen */
PROC IMPORT OUT= d.Lischker_&date
            DATAFILE= "&d\Metaanalysenliste März 23 Korrekturen Jan 24.xlsx"
            DBMS=EXCEL REPLACE;
     RANGE="Tabelle1$";
     GETNAMES=YES;
     MIXED=NO;
     SCANTEXT=YES;
     USEDATE=YES;
     SCANTIME=YES;
RUN;

data d.Lischker_&date;
set d.Lischker_&date;
if Meta = 1 then do;
  Study2 = strip(study);
  pos1 = find(Study2, ",");
  pos2 = find(substr(Study2, 1, pos1 + 1), ",");
  Authorname = substr(Study2, 1, pos1 - 1);
  Year = input(substr(Study2, pos1 + 2, 4), 4.0);
  LitReference = cat(strip(Authorname), ", ", put(Year, 4.0));
end;
select;
  when (Group =  1) GroupName = "ClearSight MAP";
  when (Group =  2) GroupName = "ClearSight SBP";
  when (Group =  3) GroupName = "ClearSight DBP";
  when (Group =  4) GroupName = "CNAP MAP";
  when (Group =  5) GroupName = "CNAP SBP";
  when (Group =  6) GroupName = "CNAP DBP";
  when (Group =  7) GroupName = "T-Line MAP";
  when (Group =  8) GroupName = "T-Line SBP";
  when (Group =  9) GroupName = "T-Line DBP";
  when (Group = 10) GroupName = "Vasotrac MAP";
  when (Group = 11) GroupName = "Vasotrac SBP";
  when (Group = 12) GroupName = "Vasotrac DBP";
  when (Group = 13) GroupName = "N-CAT MAP";
  when (Group = 14) GroupName = "N-CAT SBP";
  when (Group = 15) GroupName = "N-CAT DBP";
  when (Group = 16) GroupName = "PPG MAP";
  when (Group = 17) GroupName = "PPG SBP";
  when (Group = 18) GroupName = "PPG DBP";
  otherwise;
end;
drop Study2 pos1 pos2;
lfd + 1;
run;

/* Leere Zeilen und Spalten löschen */
data d.Lischker_&date;
set d.Lischker_&date (where=(group^=. and Meta ^=. and weight^=0));
```

```
run;
data d.Lischker_Studies_only_&date;
set d.Lischker_&date;
where meta = 1;
run;
proc sort data=d.Lischker_Studies_only_&date;
by Group Study;
run;

/* Korrekturen anbringen - vertauschte Limits of agreement (wegen umgekehrten Vorzeichen
des Bias) */
data d.Lischker_Studies_only_&date;
set d.Lischker_Studies_only_&date;
by Group Study;
if first.Group then StudyNr = 1;
else StudyNr + 1;
delta1 = Bias - mean(LLOA, ULOA);
delta2 = Bias_roh - mean(LLOA, ULOA);
if (abs(delta1) > abs(delta2) & ReferenzMinusNeu = "nein") then do;
 x = LLOA;
 LLOA = -1*ULOA;
 ULOA = -1*x;
end;
delta1 = Bias - mean(LLOA, ULOA);
delta2 = Bias_roh - mean(LLOA, ULOA);
drop delta1 delta2 x;
run;

proc contents data=d.Lischker_&Date varnum; run;
proc contents data=d.Lischker_studies_only_&Date varnum; run;

title;
footnote;
options orientation=portrait;
ods rtf close;

dm log "file '&date &filename..log'";
```
