# Supplementary material for: Accuracy and precision of continuous, non‐invasive blood pressure devices: a systematic review and meta‐analyses
Source: Anaesthesia. 2026 Jun 15;81(11):1581–92. doi: 10.1111/anae.70250 (PMC13659784; doi:10.1111/anae.70250)
Supplement: Supplementary file 4 [file ANAE-81-1581-s002.docx]

**Appendix S1:** Search process according to PRISMA‑guidelines:

Following search strategy was applied:
Single-word search („blood pressure“, „arterial pressure“, „non-invasive“, „continuous*“, „finger blood pressure“, „Nexfin“, „CNAP“, „CareTaker medical“, „T-Line“, Vasotrac“, „Penaz“, „volume-clamp“, „pulse transit time“, „pulse wave velocity“, „pulse decomposition analysis“, „Bland-Altman“, „limits of agreement“, „bias“, „applanation tonometry“, „vascular unload*“)
and also using the PICO-tool (patient, intervention, comparison, outcome):

- Patient – blood pressure OR BP OR blood-pressure OR arterial pressure OR AP
- Intervention – Nexfin OR ClearSight OR CNAP OR T-Line OR TL-300 OR TL-400 OR TL-300pro OR TL-200 OR applanation tonometry OR AP OR Tensys OR Vasotrac OR NCAT OR Tonometry OR BPro OR Omron OR volume clamp OR Wesseling OR finger cuff OR vascular unloading OR PPG OR PWV OR PTT OR „pulse transit time“ OR „pulse wave velocity“ OR plethysmograph* OR „pulse decompositions analysis“ OR PDA
- Comparison – arterial line OR AL OR radial OR catheter OR invasive Outcome – comparison OR Bland Altman OR bias OR LOA OR „limits of agreement“ OR Bland OR mean difference.
